# Paralogs in the PKA Regulon Traveled Different Evolutionary Routes to Divergent Expression in Budding Yeast

**DOI:** 10.3389/ffunb.2021.642336

**Published:** 2021-04-27

**Authors:** Benjamin M. Heineike, Hana El-Samad

**Affiliations:** ^1^Bioinformatics Graduate Program, University of California, San Francisco, San Francisco, CA, United States; ^2^Department of Biochemistry and Biophysics, California Institute for Quantitative Biosciences, University of California, San Francisco, San Francisco, CA, United States; ^3^Chan Zuckerberg Biohub, San Francisco, CA, United States

**Keywords:** yeast, evolution, PKA, comparative transcriptomics, allopolyploidization, environmental stress response, genome duplication

## Abstract

Functional divergence of duplicate genes, or paralogs, is an important driver of novelty in evolution. In the model yeast *Saccharomyces cerevisiae*, there are 547 paralog gene pairs that survive from an interspecies Whole Genome Hybridization (WGH) that occurred ~100MYA. In this work, we report that ~1/6th (110) of these WGH paralogs pairs (or ohnologs) are differentially expressed with a striking pattern upon Protein Kinase A (PKA) inhibition. One member of each pair in this group has low basal expression that increases upon PKA inhibition, while the other has moderate and unchanging expression. For these genes, expression of orthologs upon PKA inhibition in the non-WGH species *Kluyveromyces lactis* and for PKA-related stresses in other budding yeasts shows unchanging expression, suggesting that lack of responsiveness to PKA was likely the typical ancestral phenotype prior to duplication. Promoter sequence analysis across related budding yeast species further revealed that the subsequent emergence of PKA-dependence took different evolutionary routes. In some examples, regulation by PKA and differential expression appears to have arisen following the WGH, while in others, regulation by PKA appears to have arisen in one of the two parental lineages prior to the WGH. More broadly, our results illustrate the unique opportunities presented by a WGH event for generating functional divergence by bringing together two parental lineages with separately evolved regulation into one species. We propose that functional divergence of two ohnologs can be facilitated through such regulatory divergence.

## Introduction

Gene duplication is considered an important source of novelty and adaptation in evolution (Ohno, 1970; Taylor and Raes, 2004; Hittinger and Carroll, 2007; Conant and Wolfe, 2008; Des Marais and Rausher, 2008). In particular, the duplication of entire genomes, or Whole Genome Duplications (WGD), have been hypothesized to offer a unique avenue to generate evolutionary diversity by allowing members of complexes and metabolic pathways to be retained as paralogs that are not often retained in Small Scale Duplications (Conant and Wolfe, 2007; Guan et al., 2007; Wapinski et al., 2007; van Hoek and Hogeweg, 2009).

**Figure 1:**
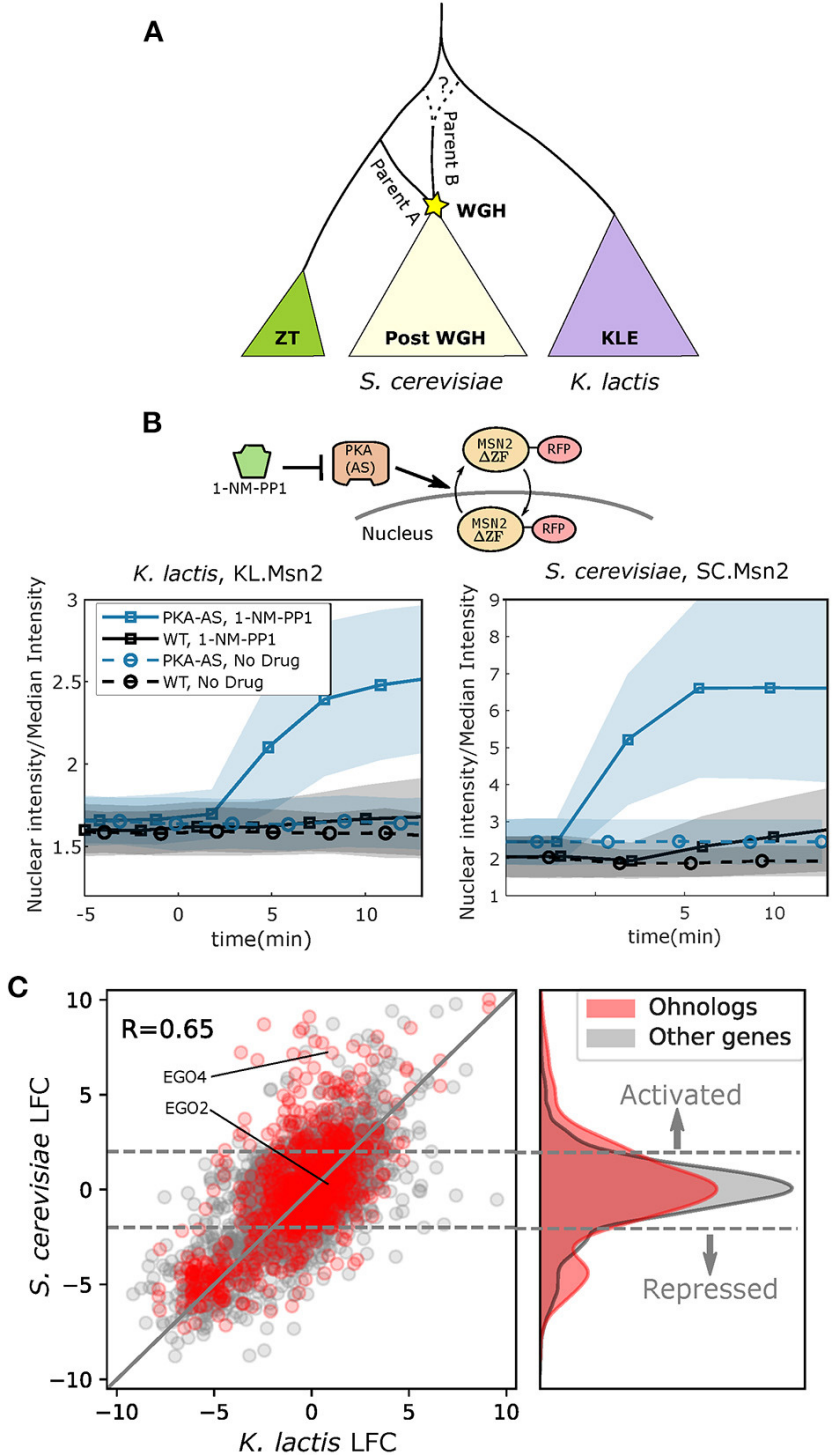
Ohnologs are enriched in genes affected by PKA inhibition in *S. cerevisiae*. **(A)** Simplified schematic depicting the budding yeast Whole Genome Hybridization (WGH). The *Kluyveromyces/Lachancea/Eremothecium* (KLE) branch is shaded purple, and the *Zygosaccharomyces/Torulaspora* (ZT) branch is shaded green. Parent A is more closely related to the ZT branch than parent B. The dashed line illustrates the fact that the phylogenetic branch point of parent B has not been fully resolved and may be early on the ZT branch or on the KLE branch. **(B)** Msn2 nuclear localization in *K. lactis* (left) and *S. cerevisiae* (right) for WT and PKA Analog Sensitive (PKA-AS) strains following the addition of control media or 4uM 1-NM-PP1. Solid line represents the mean and the shaded area represents the standard deviation for at least 27 single cell measurements in *K. lactis* and at least 84 single cell measurements in *S. cerevisiae*. A diagram of the experimental system is shown. PKA-AS drives nuclear export of Msn2(ΔZF)-RFP (denoted by SC.Msn2 in *S. cerevisiae* or KL.Msn2 in *K. lactis*) and is inhibited by 1-NM-PP1. **(C)** Log Fold Change (LFC) comparing RNA sequencing data collected from strains in which PKA-AS was inhibited with 3uM 1-NMPP1 vs. DMSO controls. Data was collected after 50 min in both *S. cerevisiae* (*y*-axis) and *K. lactis* (*x*-axis) following administration of the drug. LFC values are only shown for genes that had orthologs in both species. Genes that are ohnologs in *S. cerevisiae* are colored red, and all other genes are colored gray. The *y* = *x* line is shown in gray. The differentially expressed ohnologs EGO2 and EGO4, which share a common ortholog in *K. lactis*, are highlighted. Horizontal dashed gray lines indicate LFC values of 2.0 and −2.0 which are thresholds used for identifying activated and repressed genes. The kernel density estimate of the distribution of LFC for ohnologs in *S. cerevisiae* is shown to the right of the scatter plot. The distribution of LFC for all other genes is shown in gray for comparison.

Much of the understanding we have of WGD comes from work done on the model organism *Saccharomyces cerevisiae*, the first eukaryote to have its genome fully sequenced (Goffeau et al., 1996). Not long after the *S. cerevisiae* genome was published, researchers discovered that its lineage had undergone a WGD approximately 100Mya (Wolfe and Shields, 1997; Kellis et al., 2004). Paralogs resulting from a WGD are known as “ohnologs,” and today *S. cerevisiae* retains 547 ohnologs from its WGD, comprising nearly 18% of its annotated protein coding genes (Byrne and Wolfe, 2005). As more budding yeast genomes have been sequenced, a greater understanding has developed surrounding the budding yeast WGD. One particularly surprising discovery was that the WGD event was most likely an allopolyploidization, or hybridization, between two species that had separated between, by one estimate, 15–25 million years prior (Marcet-Houben and Gabaldón, 2015). As a result, a more apt description for the event would perhaps be a Whole Genome Hybridization (WGH). Of the two species that hybridized during the WGH, one was closely related to the ancestor of the *Zygosaccharomyces/Torulospora* (ZT) branch of non-WGH species (parent A in Figure 1A). The other likely separated from the ancestral lineage closer to the time when the ancestor of the *Kluyveromyces/Lachancea/Eremothecium* (KLE) branch diverged (parent B in Figure 1A).

Several characteristic phenotypes underwent striking changes following the WGH, including a drastic change in metabolic lifestyle between Crabtree positive yeast (post-WGH species) that use fermentation to metabolize glucose when oxygen is present and Crabtree negative yeast (most non-WGH yeast species) that use respiration (Merico et al., 2007; Hagman and Piškur, 2015). There were also major changes in the Environmental Stress Response (ESR) following the WGH (Roy et al., 2013; Thompson et al., 2013). The ESR is a common gene expression program consisting of hundreds of genes that are either activated or repressed in a variety of stressful conditions such as glucose depletion, osmotic shock, and oxidative stress (Gasch et al., 2000; Causton et al., 2001). Previous studies have demonstrated that much of the ESR is conserved across budding yeast species, and that the level of conservation depends on whether the genes are activated or repressed in the ESR. The set of genes repressed in the ESR, which contains genes related to ribosomal biogenesis and protein production, is more conserved across different yeast lineages. By contrast, membership in the activated ESR is more variable (Gasch et al., 2000; Thompson et al., 2013). Several of the evolutionary changes that occurred in the activated ESR following the WGH are consistent with changes in metabolic lifestyle between Crabtree and non-Crabtree yeast. Such changes include induction of more amino acid biosynthesis, purine biosynthesis, and oxidative phosphorylation genes as well as repression of fewer mitochondrial genes in post-WGH species compared to non-WGH species (Thompson et al., 2013).

The Protein Kinase A (PKA) pathway is one of the primary master regulators of the ESR (Gasch et al., 2000; Zaman et al., 2009; Mace et al., 2020). PKA is a negative regulator of the ESR, in the sense that when it is active, the ESR is inactive, and vice versa. Cells with a hyperactive PKA pathway are unable to induce the ESR when required, causing them to become sensitive to stress and fail to grow on carbon sources that require respiration (Toda et al., 1987; Jiang et al., 1998). While comparative analyses of gene expression between budding yeast species have described broad patterns of conservation and divergence of the genes in the ESR that coincide with the emergence of the respiro-fermentative lifestyle (Roy et al., 2013; Thompson et al., 2013; Brion et al., 2016), it is not clear whether those differences result from an evolutionary rewiring of the ESR downstream of PKA or whether they are a result of rewiring upstream of PKA. Since the PKA pathway is able to integrate a variety of stimuli to trigger the ESR, understanding how it has been rewired could give us insight into how yeast species have evolved to cope with the unique stresses of their particular ecological niches.

In this work, we probed the specific evolutionary rewiring of the PKA program following the WGH by directly inhibiting PKA in both *K. lactis*, a species from a non-WGH lineage, and *S. cerevisiae*, the canonical post-WGH species, and compared the ensuing gene expression profiles. To do this we generated strains in each species containing ATP analog sensitive (AS) allelles for PKA, a strategy that has been used extensively to study PKA signaling in *S. cerevisiae* (Zaman et al., 2009; Hao and O'Shea, 2011; Hansen and O'Shea, 2015a,b, 2016; Hansen et al., 2015; Mace et al., 2020). We identified 110 WGH ohnologs in *S. cerevisiae* that showed differential expression under PKA inhibition, with one ohnolog induced and the other with constant or decreased expression. Furthermore, on average within this set, the ohnolog that was induced by PKA inhibition had lower basal expression than the uninduced ohnolog. The phenotype for the shared ortholog in *K. lactis* largely resembled that of the uninduced ohnolog—moderate basal expression and no induction following PKA inhibition, leading us to hypothesize that that was the ancestral phenotype. We probed this hypothesis using publicly available gene expression data for a range of budding yeast species spanning the WGH (Tsankov et al., 2010; Roy et al., 2013; Thompson et al., 2013). Our investigations supported our hypothesis that the ancestral transcriptional response to PKA inhibition for these differentially expressed ohnologs generally featured moderate basal expression and low induction similar to the *K. lactis* ortholog. We therefore explored the evolutionary trajectories of PKA dependence in the regulation of these genes. To do so, we carried out phylogenetic analysis of binding sites in gene promoter sequences from a large set of recently published budding yeast genomes (Shen et al., 2018), focusing on the DNA binding motif for Msn2 and Msn4, the canonical transcription factors downstream of PKA. These analyses revealed that for some genes, divergence in regulation by PKA between ohnologs occurred after the WGH, while for others, differential expression arose when the ancestors of the paralogs were in different species prior to the WGH.

Allopolyploidization (WGH) events have occurred at key evolutionary junctions in plants (del Pozo and Ramirez-Parra, 2015) and vertebrates (Matos et al., 2015). Although there have been relatively few ancient WGD and WGH events observed so far in fungi [the budding yeast WGH being one of two (Ma et al., 2009; Corrochano et al., 2016)], there is reason to believe that they are more widespread in fungi but have remained undetected due to difficulties in obtaining karyotype data from fungi, higher chromosome plasticity and more pressure for reduced genome size (Campbell et al., 2015; Naranjo-Ortiz and Gabaldón, 2020). The examples we describe could therefore illustrate principles for generating novelty in evolution that might be a general feature in evolution across the spectrum of life.

## Results

### A PKA Analog Sensitive Allele in *K. lactis* Allows for Comparing the Transcriptional Response to PKA Inhibition With That of *S. cerevisiae*

Information about stress and carbon source availability is transduced within budding yeast cells through the second messenger cyclic adenosine monophosphate (cAMP). During exponential growth in glucose, cAMP levels are high and the abundant cAMP binds to the regulatory subunit of the PKA hetero-tetramer, releasing the active subunit to phosphorylate PKA targets. Conversely, when cAMP levels drop, its dissociation from PKA inhibits PKA activity, allowing dephosphorylation of PKA targets. This causes global changes to the transcriptional and metabolic state of the cell and results in a severe slowdown of growth which largely corresponds with the ESR (Jiang et al., 1998).

To compare the response to PKA inhibition between *K. lactis*, a species that diverged prior to the WGH (non-WGH), and the model species *S. cerevisiae* whose ancestor underwent the WGH (post-WGH), we used a gatekeeper mutation strategy to allow precise chemical control of PKA's kinase activity (Bishop et al., 2000; Blethrow et al., 2004; Islam, 2018). For PKA in *S. cerevisiae*, this strategy consists of mutating a “gatekeeper” methionine residue to a less bulky glycine residue for all three PKA catalytic subunit isoforms (M164G, M147G, and M165G for TPK1, TPK2, and TPK3, respectively). The resulting expanded active site still accepts ATP and allows the kinase to phosphorylate its targets with close to wild-type efficiency. However, when a bulky ATP analog (1-NM-PP1) is provided in the media, it effectively inhibits any PKA activity. This PKA analog sensitive (AS) allele strategy was previously used to investigate many properties of PKA signaling in *S. cerevisiae* (Zaman et al., 2009; Hao and O'Shea, 2011; Hansen and O'Shea, 2015a,b, 2016; Hansen et al., 2015; Mace et al., 2020).

While strains with gatekeeper mutations to PKA in *S. cerevisiae* were available, there were no such strains for *K. lactis*. To inhibit PKA in *K. lactis*, we constructed a strain containing gatekeeper mutations in each of the two PKA catalytic subunit genes present in that species (M139G for *KLLA0D03190* (*KL.TPK2*) and M222G for *KLLA0B07205* (*KL.TPK3*). We used a single plasmid CRISPR/Cas9 gene editing strategy (Ryan and Cate, 2014), and delivered the Cas9 expression cassette and sgRNA on a universal ARS plasmid that allows for stable expression in a number of budding yeast species (Liachko and Dunham, 2014). Addition of 1-NM-PP1 to both *S. cerevisiae* and *K. lactis* strains containing PKA-AS alleles stalled growth in both species (Supplementary Figure 1A) but had only a modest effect on strains bearing the WT alleles, suggesting successful inhibition of PKA in *K. lactis* given the known role of PKA in growth control.

To further validate our ability to inhibit PKA, we sought to measure the nuclear translocation of Msn2. In *S. cerevisiae*, the paralogous transcription factors Msn2 and Msn4 are direct targets of PKA. When they are phosphorylated by active PKA they remain in the cytoplasm, and when dephosphorylated, they translocate into the nucleus and activate a majority of the genes that make up the activated portion of the ESR (Gasch et al., 2000; Görner et al., 2002; Hao and O'Shea, 2011; Stewart-Ornstein et al., 2013). The *K. lactis* ortholog of Msn2 and Msn4, KLLA0F26961g (KL.Msn2), has been previously shown to be required for PKA dependent regulation of mating pathway genes (Barsoum et al., 2011). To directly investigate whether nuclear localization of KL.Msn2 is regulated by PKA as its orthologs (Msn2/4) are in *S. cerevisiae*, we constitutively overexpressed a fluorescently tagged KL.Msn2(C623S)-RFP in the PKA-AS strain. This Msn2 allele contains a mutation in a conserved cysteine in the zinc-finger domain that abolishes binding to DNA, therefore minimizing the effects of overexpressing the transcription factor (Stewart-Ornstein et al., 2013). For comparison, we also constructed a similar Msn2 reporter in a *S. cerevisiae* PKA-AS strain [SC.Msn2(C649S)-RFP]. Following addition of 1-NM-PP1 to *K. lactis* PKA-AS cells, KL.Msn2-RFP became enriched in the nucleus, just as SC.Msn2-RFP did in *S. cerevisiae* (Figure 1B, Supplementary Figure 1B). These data indicate that the nuclear localization of Msn2, and therefore its ability to promote transcription, is regulated by PKA in both species.

### The Transcriptional Responses to PKA Inhibition in *S. cerevisiae* and *K. lactis* Share Many Similarities but Also Exhibit Clear Functional Differences

To gain a broader understanding of the similarities and differences in the global transcriptional response to PKA inhibition between *S. cerevisiae* and *K. lactis*, we carried out mRNA sequencing for both species. We prepared sequencing libraries from exponentially growing PKA-AS cultures collected 50 min after providing saturating amounts of 1-NM-PP1 (3 μM) or a DMSO control. We computed the Log Fold Change (LFC) in gene expression using the DESEQ2 computational framework by comparing read counts from yeast treated with drug to read counts from control cultures (Love et al., 2014). We also calculated regularized logarithm estimates (rlog) within the DESEQ2 framework to evaluate expression levels in each condition. The transcriptional responses we observed were broadly correlated between the two species (Pearson Correlation = 0.65) (Figure 1C scatter plot). This was consistent with the observation that the ESR, and particularly the repressed ESR, is conserved in budding yeasts (Thompson et al., 2013).

To identify shared and species-specific functional enrichment, we first defined sets of genes that were induced or repressed by PKA inhibition in each species. We looked for genes that had LFC >2.0 for activated genes and < -2.0 for repressed genes, and which were significant with a False Discovery Rate (FDR) of 1% (Supplementary Figure 2). Focusing only on genes that had orthologs in both species, we further subdivided these target sets into subsets that were either (i) activated in both species, (ii) repressed in both species, (iii) activated in one species but not the other, or (iv) repressed in one species but not the other. We then quantified enrichment for GO-slim terms (*SGD Project*, 2018) for the different gene subsets using Fisher's exact test (Supplementary Table 1, Supplementary Figure 3). For functional enrichment of *K. lactis* genes, we only analyzed genes with orthologs in *S. cerevisiae* and assigned go terms based on the *S. cerevisiae* orthologs (see Methods).

A few broad themes emerged from these analyses. First, genes related to growth and protein production in the ribosome were repressed in both species (“rRNA processing,” *p* = 7.3e-86, and “cytoplasmic translation,” *p* = 8.1e-72), consistent with previous work relating PKA inhibition to a slowdown in growth (Brauer et al., 2008; Airoldi et al., 2009) and the slowdown in growth we observed following PKA inhibition in both species (Supplementary Figure 1A).

Additionally, while PKA inhibition triggered a set of conserved gene expression changes related to metabolic processes in both species, there was evidence in *S. cerevisiae* specifically of PKA's previously observed role in the shift from fermentative to respiratory metabolism (Ihmels et al., 2005; Field et al., 2008; Tsankov et al., 2010; Thompson et al., 2013). Firstly, ‘mitochondrial translation' genes were repressed only in *K. lactis* following PKA inhibition (*p* = 6.3e-42). Also, there was evidence of a broad metabolic reconfiguration in *S. cerevisiae* that was not shared with *K. lactis*. In the set of genes that were specifically induced in *S. cerevisiae* but not in *K. lactis* following PKA inhibition there was enrichment for terms related to respiratory metabolism (“cellular respiration,” *p* = 1.4e-6 and “response to oxidative stress,” *p* = 6.1e-5), as well as other metabolic terms (e.g., “generation of precursor metabolites and energy,” *p* = 9.4e-10). This metabolic shift was also evident in the set of genes that was repressed specifically in *S. cerevisiae*, which were enriched for the term “cellular amino acid metabolic process” (*p* = 8.38e-7).

### Many Ohnolog Pairs Are Differentially Activated by PKA Inhibition in *S. cerevisiae*

Since *S. cerevisiae* has undergone a WGH, we were curious whether its surviving ohnologs were equally represented in the PKA regulon compared to other genes that didn't maintain their paralogous copy. Interestingly, we found that in the context of PKA inhibition, ohnologs were enriched both in the set of genes that were activated (*p* = 5.2e-11) and those that were repressed (*p* = 2.6e-6), while being de-enriched in the set of genes whose expression didn't change (*p* = 9.2e-5) (Figure 1C right panel, Supplementary Table 2).

**Figure 2:**
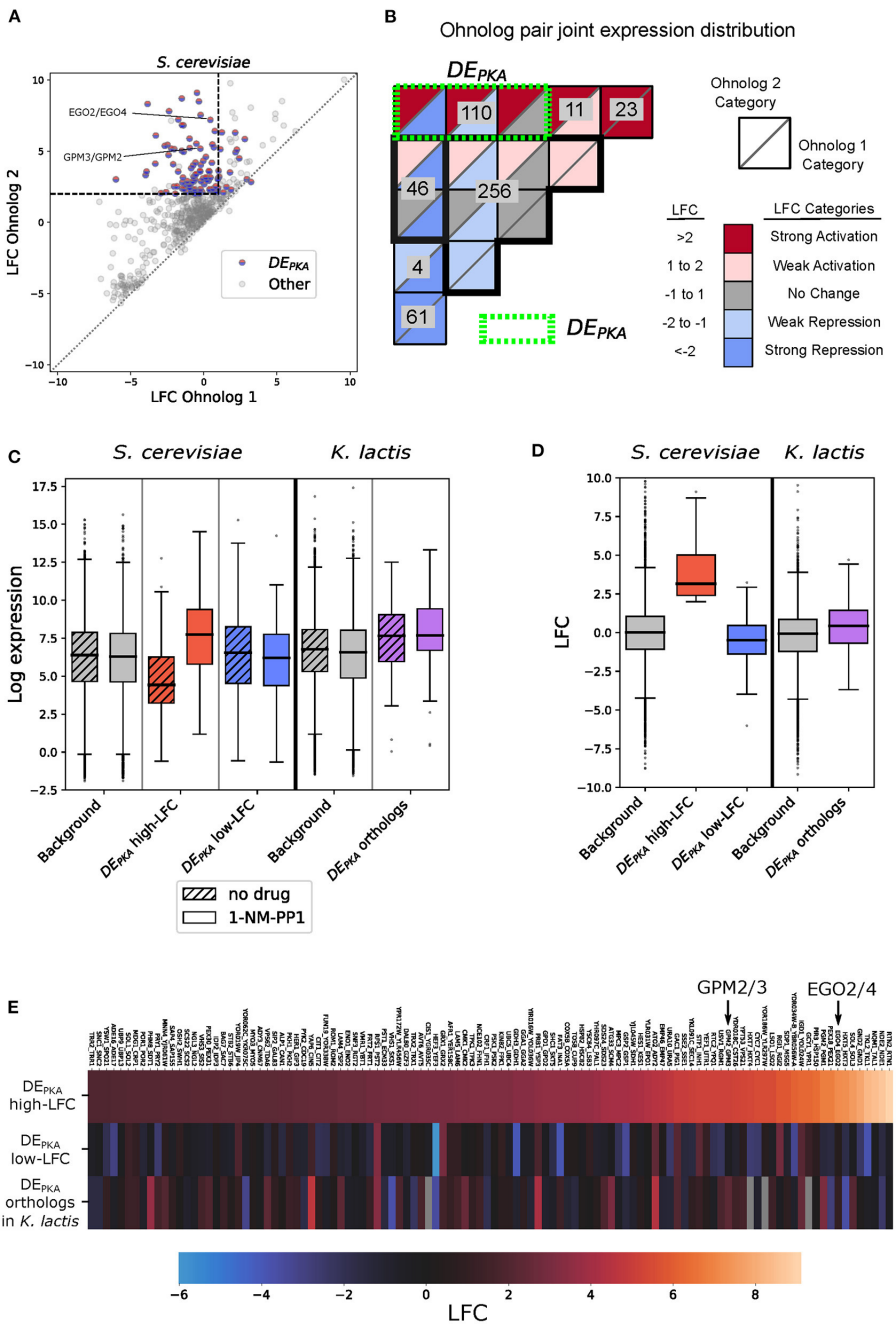
One hundred ten ohnolog pairs show differential expression in response to PKA. One member of the pair is activated while the other is repressed or unaffected. The phenotypes of the shared orthologs from *K. lactis* on average resemble the ohnologs that are not activated by PKA inhibition. **(A)** Comparison of Log Fold Change (LFC) from mRNA sequencing data following PKA inhibition for ohnolog pairs in *S. cerevisiae*. Each pair of ohnologs is sorted so that Ohnolog 1 (*x*-axis) has lower LFC than Ohnolog 2 (*y*-axis). In cases where one ohnolog did not meet the 1% FDR threshold while the other ohnolog did, the LFC value for the ohnolog which did not meet the threshold was replaced with 0 for sorting, but the original value is plotted. Dashed lines indicate the LFC criteria for selecting differentially expressed ohnologs, in which one member of the pair is induced by PKA inhibition (DE_PKA_). DE_PKA_ genes do not correspond exactly with ohnolog pairs in that region because genes that do not meet the 1% FDR threshold are classified as having no change even if their LFC is in the appropriate range. **(B)** Joint distribution of ohnolog pairs in various categories based on the LFC of each ohnolog in response to PKA inhibition. The bins are arranged based on the LFC categories for Ohnolog 1 and Ohnolog 2. For each category, the shading of the top triangle represents the LFC of Ohnolog 2, and the bottom triangle represents the LFC of Ohnolog 1. The green dotted line defines DE_PKA_ as ohnolog pairs that are differentially expressed under PKA inhibition and in which one ohnolog is strongly activated. **(C)** Estimated regularized log expression (rlog) for DE_PKA_ ohnologs (sorted by high and low activation) and their orthologs in *K. lactis*. The background set for *S. cerevisiae* includes all annotated genes except those designated as dubious orfs in SGD. The background set for *K. lactis* includes all annotated genes with orthologs in *S. cerevisiae*. **(D)** LFC for DE_PKA_ ohnologs (sorted by high and low activation) and their orthologs in *K. lactis*. Background sets as in **(C)**. **(E)** LFC for high-LFC ohnologs (top row) and low-LFC ohnologs (middle row) from the DE_PKA_ ohnolog set in *S. cerevisiae* as well as their *K. lactis* orthologs (bottom row). Columns are sorted from lowest to highest LFC for the high-LFC ohnolog. Example ohnolog pairs referred to later in the text are highlighted.

To further explore this observation and determine whether ohnolog pairs behaved similarly upon PKA inhibition, we ordered them by LFC and plotted the LFC of each ohnolog against the other for all pairs (Figure 2A). Of the 184 ohnolog pairs that had at least one member repressed, 71 pairs had both members repressed. Previous work has observed that cytoplasmic ribosomal proteins are enriched in retained *S. cerevisiae* ohnologs (Seoighe and Wolfe, 1999), and we saw that the set of genes for which both ohnologs were strongly repressed by PKA inhibition was enriched for ribosomal genes. In our dataset we had 56 ohnolog pairs associated with the GO-slim term ‘cytoplasmic translation', including many ribosomal proteins, and 52 of those pairs had both members repressed (Supplementary Figure 4).

Many ohnolog pairs, in contrast, had different patterns of expression in response to PKA inhibition. To analyze these, we focused on ohnolog pairs in which one member was activated by PKA inhibition and one member was not. There were 110 such ohnolog pairs, which we will simply refer to as DE_PKA_ in the following (Figure 2B, Supplementary Table 3). The DE_PKA_ genes were enriched for metabolic go terms such as “nucleobase-containing small molecule metabolic process,” “generation of precursor metabolites and energy,” and “cofactor metabolic process” (Supplementary Figure 5, Supplementary Table 4, Supplementary analysis of DE_PKA_ genes). The group also included several pairs of regulators related to metabolic processes, including TPK1/3 themselves. A total of 18 DE_PKA_ ohnolog pairs coded for enzymes included in the iPath3 database (Darzi et al., 2018). Projecting these onto the metabolic map of yeast revealed that they were concentrated around central carbon metabolism including key enzymes in glycolysis/gluconeogenesis, the Pentose Phosphate Pathway (PPP), the Tricarboxylic Acid (TCA) cycle, and oxidative phosphorylation (Supplementary Figure 6). This is consistent with previous work that observed several of the same ohnolog pairs expressed differentially in response to growth to saturation in batch culture, a condition which causes PKA inhibition (Thompson et al., 2013). In particular, a large proportion (8 of 12) of the enzymes in the Pentose Phosphate pathway were members of DE_PKA_ ohnolog pairs. Clustering the DE_PKA_ ohnolog pairs based on a curated list of GO-slim terms revealed other groups of paralog pairs with terms in common. This included groups of between 3 and 8 ohnolog pairs sharing the Biological Processes terms “response to oxidative stress,” “peroxisome activity,” “lipid transport,” “endocytosis,” and “golgi vesicle transport,” and the Molecular Function terms “transmembrane transport,” “kinase activity,” “enzyme regulator activity,” “nucleic acid binding transcription factor activity,” and “lipid binding” (Supplementary Figure 4). Respectively, 23 and 37 of the DE_PKA_ ohnolog pairs did not have a Biological Process or Molecular Function GO-slim term assigned for at least one ohnolog. This was reflective of the overall level of genes without Biological Process or Molecular Function terms assigned in all genes posessing RNA-seq data in the study.

We then explored the properties of the DE_PKA_ ohnolog in more depth beginning with an analysis of their basal expression levels. The basal expression levels for the ohnologs with higher LFC values (which we refer to as the DE_PKA_ high-LFC ohnologs) were, on average, low compared to all genes in the genome (median rlog 4.4 vs. 6.4, Figure 2C, Supplementary Figure 7, Supplementary Table 4). Upon PKA inhibition, the distribution of expression of these DE_PKA_ high-LFC ohnologs shifted clearly higher than that of all genes in the genome (median rlog of 7.7), reflecting the induction for which they were defined (Figures 2C–E). By contrast, the distribution of basal expression values for the DE_PKA_ genes that were unchanged or repressed following PKA inhibition (DE_PKA_ low-LFC ohnologs) was generally close to background levels whether or not PKA was inhibited (median rlog of 6.5 and 6.2 without and with 1-NM-PP1, respectively) (Figure 2C). Importantly, the distribution of basal expression of the DE_PKA_ high-LFC ohnologs had a clear lower median than that of the DE_PKA_ low-LFC ohnologs (Figure 2C). This indicates that the difference in PKA responsiveness between these two sets is the result of low basal expression and strong induction for the DE_PKA_ high-LFC ohnologs, and higher basal expression that was either slightly reduced or unchanged by PKA inhibition for DE_PKA_ low-LFC ohnologs. *K. lactis* contains orthologs for 106 of the 110 ohnolog pairs in the DE_PKA_ set. As a group, these orthologs had a similar phenotype to that of the DE_PKA_ low-LFC ohnologs, displaying higher basal expression (Figure 2C, Supplementary Figure 7) and little change in expression under PKA inhibition (Figures 2C–E). This observation led us to hypothesize that the ancestral phenotype for many of these ohnolog pairs was one with moderate basal expression and no regulation by PKA.

### Analysis of Stress-Response Data Across Species Suggests That Insensitivity to PKA Related Stresses and Moderate Basal Expression Was the Ancestral State for Most DE_PKA_ Genes

**Figure 3:**
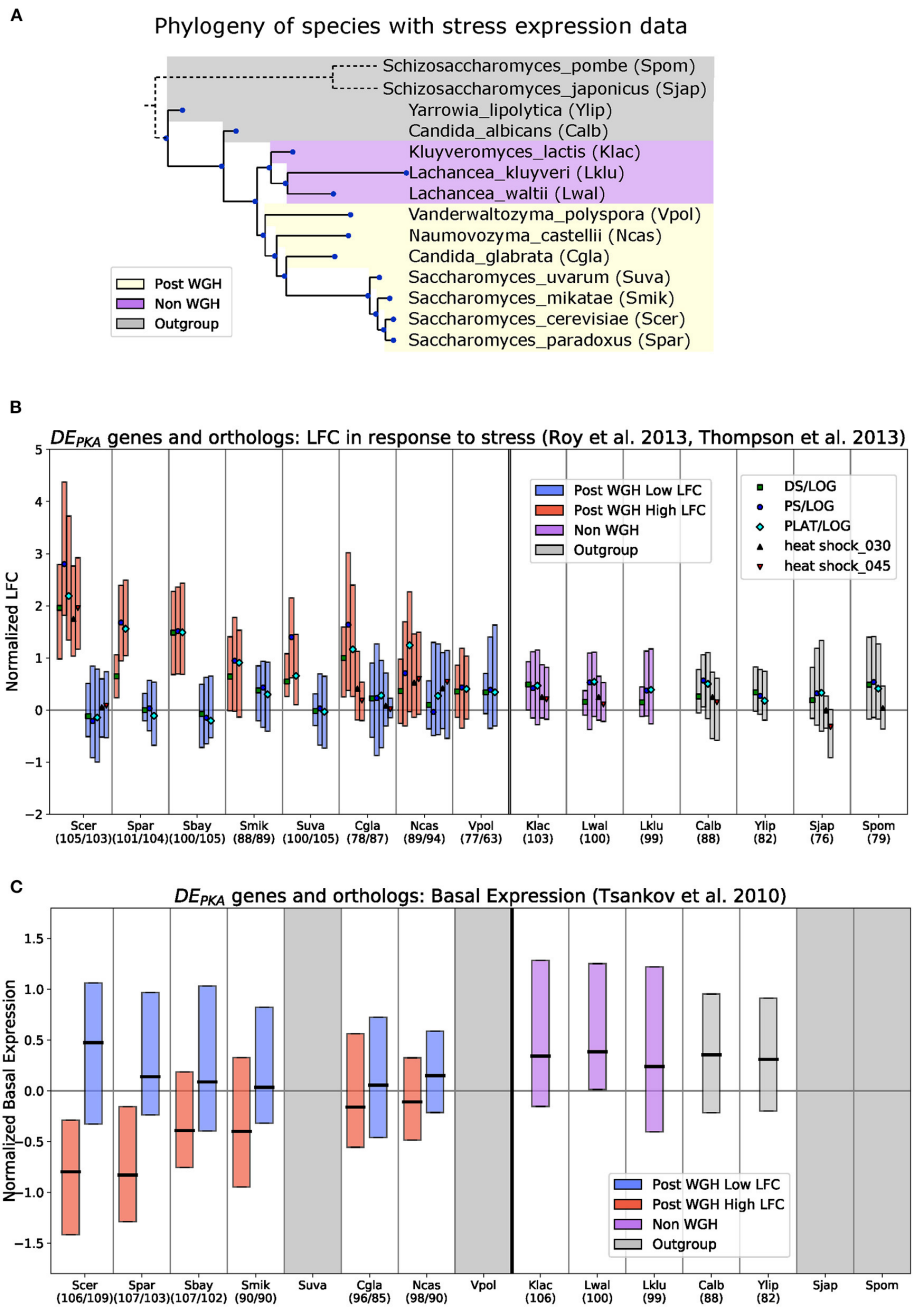
For DE_PKA_ genes, low LFC in response to PKA related stress and moderate basal expression is the ancestral phenotype. **(A)** Phylogeny of species with stress expression data used in **(B)** based on the time calibrated tree generated in Shen et al. (2018). *S. bayanus* is not shown because it is a hybrid between *S. eubayanus* and *S. uvarum* with some genes from *S. cerevisiae* (Libkind et al., 2011). *S. pombe* and *S. japonicus* branches are not drawn to scale. Boxplots in **(B,C)** show median Q1–Q3 range for the datasets described below for DE_PKA_ genes in *S. cerevisiae* and their orthologs in each indicated species (when present). Blue and red bars indicate low-LFC and high-LFC ohnologs (respectively), and their syntenic orthologs in Post-WGH species. Purple and gray bars are for the shared orthologs in Non-WGH *Saccharomycetaceae* species and outgroups, respectively. Numbers in parentheses indicate the number of retained orthologs. Syntenic ortholog assignment for Post-WGH species is based on the YGOB database (Byrne and Wolfe, 2005). Boxplots in **(B)** show normalized gene expression (see Materials and Methods) for the conditions most closely related to PKA inhibition in *S. cerevisiae* and *K. lactis* from Roy et al. (2013), Thompson et al. (2013) (Supplementary Figure 8). Medians are marked with symbols that indicate the conditions. “DS/LOG,” “PS/LOG,” and “PLAT/LOG” are from Thompson et al. (2013) and “heat shock_030” and “heat shock_045” are from Roy et al. (2013). Boxplots in **(C)** show normalized raw expression data (see Materials and Methods) from microarray experiments comparing mRNA under exponential growth conditions to genomic DNA from Tsankov et al. (2010). Gray regions indicate species for which no data exist. Species: Scer, *Saccharomyces cerevisiae*; Spar, *Saccharomyces paradoxus*; Sbay, *Saccharomyces bayanus;* Smik, *Saccharomyces mikatae*; Suva, *Saccharomyces uvarum*; Cgla, *Candida glabrata*; Ncas, *Naumovozyma castellii;* Vpol, *Vanderwaltozyma polyspora*; Klac, *Kluyveromyces lactis*; Lwal, *Lachancea waltii*; Lklu, *Lachancea kluyveri*; Calb, *Candida albicans*; Ylip, *Yarrowia lipolytica*; Sjap, *Schizosaccharomyces japonicas*; Spom, *Schizosaccharomyces pombe*.

Without gene expression measurements following PKA inhibition in a wider phylogenetic range of extant species, testing whether low LFC in response to PKA inhibition was the ancestral state of the DE_PKA_ genes cannot be done directly. However, we can explore it meaningfully across a range of 15 budding yeast species spanning the WGH (Figure 3A) by capitalizing on available gene expression datasets collected in response to growth to saturation and various stress conditions known to repress the PKA pathway in *S. cerevisiae* (Roy et al., 2013; Thompson et al., 2013). To extract the conditions that most resemble PKA inhibition, we compared expression changes in *S. cerevisiae* and *K. lactis* in these datasets to our PKA inhibition data. This comparison identified five conditions (heat shock at 30 and 45 min, Diauxic Shift, Post Diauxic Shift, and Plateau) that were strongly correlated with PKA inhibition in both species (Pearson correlation *R* > 0.65 with *K. lactis* PKA inhibition and *R* > 0.75 with *S. cerevisiae* PKA inhibition) (Supplementary Figure 8). We adopted the LFC reported for these 5 conditions (normalized per Materials and Methods) as surrogates for PKA inhibition across the species present in this dataset.

We curated the orthologs of the 110 DE_PKA_ ohnolog pairs in the various species and compared their gene expression values in the PKA-related stress conditions. The ohnolog pairs of the DE_PKA_ set generally showed the same phenotypes in *S. cerevisiae* under these stress conditions as in our PKA inhibition dataset—high LFC for one member of the pair and low LFC in the other (Figure 3B, *S. cerevisiae*). This is expected given our criteria for selecting these stress conditions. In the post-WGH species closely related to *S. cerevisiae*, the phenotype was also conserved. Syntenic orthologs (or in other words orthologs with shared genomic context) for the high-LFC ohnologs had higher LFC, and syntenic orthologs of the low-LFC ohnologs had lower LFC on average. The separation in LFC between the syntenic orthologs of the high-LFC and low-LFC ohnologs declined in more distantly related post-WGH species. In *V. polyspora*, the most distantly related post-WGH species, the orthologs of the low and high LFC ohnologs both had low LFC in stress conditions (Figure 3B, Supplementary Figure 9), although confident assignment of syntenic orthologs becomes difficult at that evolutionary distance. Importantly, for non-WGH species, the shared orthologs of the DE_PKA_ ohnolog pairs have low LFC under these PKA-related stress conditions (Figure 3B right of thick black line), consistent with our hypothesis that low LFC is generally the ancestral response to PKA inhibition for DE_PKA_ ohnolog pairs.

To explore basal expression of orthologs of the DE_PKA_ genes in these species, we turned to a different dataset that measured gene expression during exponential growth on rich media using microarray data for a range of budding yeasts (Tsankov et al., 2010). After appropriate normalization (see Materials and Methods), we checked consistency of these data to rlog estimates from our RNA seq count data for *S. cerevisiae* and *K. lactis* strains under control conditions. We observed high correlation despite the different methods by which gene expression data was collected (Pearson Correlation R = 0.69 and 0.60 for *S. cerevisiae* and *K. lactis*, respectively) (Supplementary Figure 10A). Reassuringly, there was high correlation between both datasets for the DE_PKA_ genes (R = 0.70 for the low-LFC ohnologs and 0.70 for the high-LFC ohnologs) as well as for their orthologs in *K. lactis* (R = 0.53) (Supplementary Figure 10B), instilling confidence that the various sets are coherent in their content and can be used for cross-comparison.

With these data in hand, we could compare basal expression of DE_PKA_ genes and their syntenic orthologs across species. For the post-WGH species, the syntenic orthologs of the low-LFC DE_PKA_ ohnologs generally had a higher basal expression while those of the high-LFC DE_PKA_ ohnologs generally had lower basal expression for species more closely related to *S. cerevisiae* and higher basal expression for species more distantly related. For the non-WGH species, the basal expression of the shared ortholog of a given DE_PKA_ ohnolog pair was at moderate levels on average, similar to that of the low-LFC DE_PKA_ ohnolog in *S. cerevisiae* (Figure 3C, Supplementary Figure 11).

These results were robust to redefining the ohnolog pairs of interest based on the PKA-related stress conditions rather than on the PKA-inhibition dataset (Supplementary Figures 12 13A 14A). The observation that the ancestral state of ohnolog pairs that are now differentially expressed in response to PKA-related stresses was moderate and unresponsive to these stresses was also robust to the species used as a reference. When we defined sets of differentially expressed ohnologs in *N. castellii* and *V. polyspora* using gene expression for the five PKA-related conditions (Materials and Methods for details), they had little overlap with each other and with the set defined in *S. cerevisiae* (Supplementary Figure 15). However, their orthologs in non-WGH species also had low LFC values (for ohnologs defined using *N. castellii* and *V. polyspora*) (Supplementary Figure 13) and moderate basal expression (for ohnologs defined using *N. castellii*) (Supplementary Figure 14).

Taken together, these data strongly support the notion that the ancestral state of ohnologs that are differentially expressed in response to PKA was characterized by moderate basal expression and insensitivity to PKA. The derived phenotype would therefore be characterized by high LFC in response to PKA inhibition and low basal expression.

### The STRE Is Enriched in the Promoters of the Genes Induced by PKA Inhibition in *S. cerevisiae*, and *K. lactis*

While our analysis indicated that responsiveness to PKA was typically the derived phenotype in differentially expressed ohnologs, it could not tell us whether this derived phenotype for any particular ohnolog arose before the WGH in the lineage leading to the ZT branch, or after the WGH. That is because the non-WGH species for which stress response data was present all diverged from the *S. cerevisiae* lineage at the same time or earlier than *K. lactis*. To be made with certainty, this inference would require knowledge of gene expression in the *Zygosaccharomyces/Torulospora* (ZT) branch in response to PKA related stresses. Since no such data exist, we next turned to exploring whether the promoters of the DE_PKA_ genes have a bioinformatic signal that might correlate with these phenotypes, capitalizing on the wealth of sequenced genomes in the budding yeast subphylum to formulate hypotheses about their evolutionary trajectory (Shen et al., 2018).

**Figure 4:**
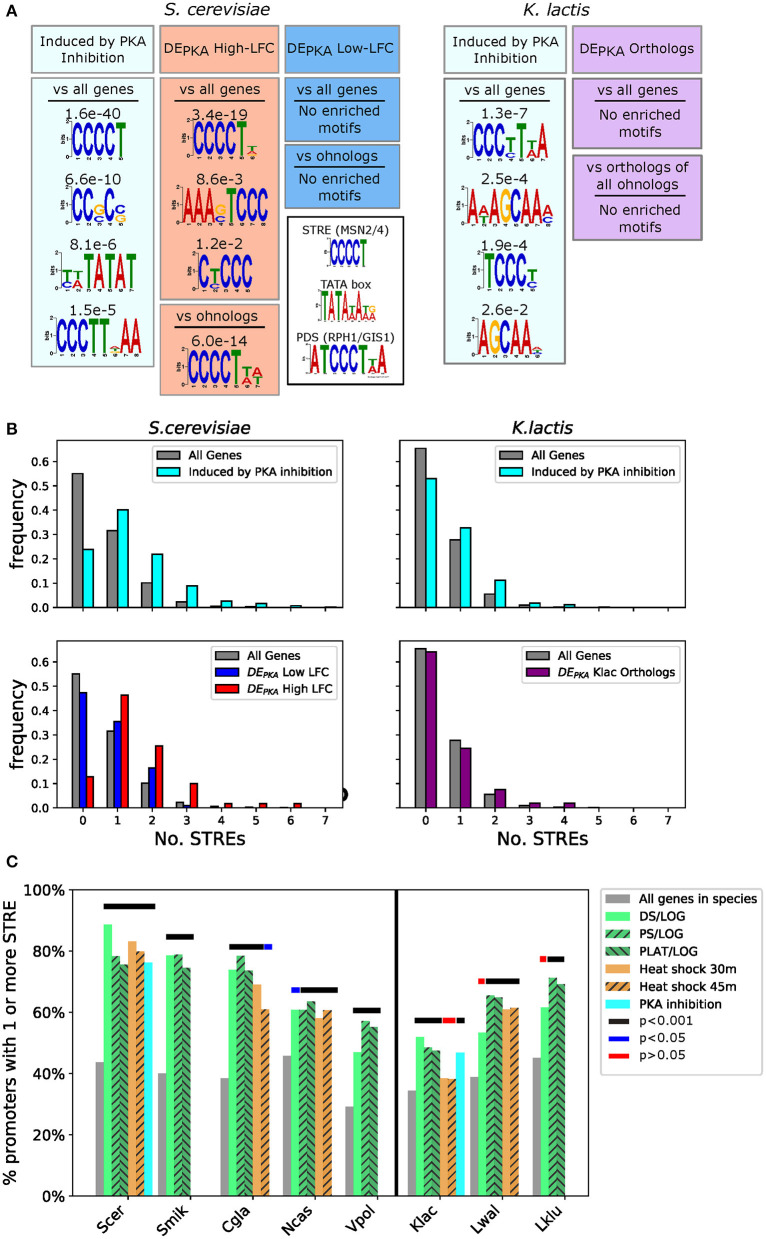
The STRE is enriched in the promoters of DE_PKA_ high-LFC ohnologs, as well as in the promoters of genes induced by PKA inhibition and PKA-related stress in various species. **(A)** Motifs identified (if any with *E* < 0.05) using the DREME algorithm from the MEME suite (Bailey, 2011) as enriched in the promoters of all *S. cerevisiae* (left) and *K.lactis* (right) genes activated by PKA inhibition (light blue box). *S. cerevisiae* enrichment for DE_PKA_ high-LFC ohnologs vs. a background of either all *S. cerevisiae* genes or all ohnologs in *S. cerevisiae* is shown in the red boxes. No motifs were identified below the threshold for the low-LFC ohnologs against either background (blue boxes). No motifs were identified above the threshold for the orthologs of the DE_PKA_ ohnolog pairs (purple boxes) vs. either a background of all genes or a background of the *K. lactis* orthologs of all ohnologs in *S. cerevisiae*. We define promoters as 700 bp upstream of the start codon for this analysis. *E*-values are indicated above each motif. The inset shows motifs for the STRE, TATA box, and PDS (Martínez-Pastor et al., 1996; Pedruzzi et al., 2000; Basehoar et al., 2004). **(B)** Histograms of the numbers of STREs in the promoters of indicated sets of genes for *S. cerevisiae* (left two plots) or *K. lactis* (right two plots). **(C)** Percentage of promoters with 1 or more STRE for all genes in a given species, or for genes that have LFC values above 2.5 for stresses related to PKA inhibition [data from Roy et al. (2013), Thompson et al. (2013)]. The vertical black line separates non-WGH from post-WGH species. Thick lines above the bars mark conditions for which number of STREs >1 was statistically different than all genes in that species using Fisher's exact test. Black: *p* < 1.0e-3, blue: *p* < 5.0e-2, red: *p* > 5.0e-2 (not significant).

To generate a framework for evaluating the characteristics of DE_PKA_ gene promoters, we first investigated bioinformatic signals associated with the promoters of all genes activated by PKA inhibition. Comparing the promoters of all genes activated under PKA inhibition in *S. cerevisiae* against the promoters of all *S. cerevisiae* genes, we identified the Stress Response Element (STRE, CCCCT), the canonical binding sequence for Msn2 and Msn4 (Martínez-Pastor et al., 1996), as the most heavily enriched motif (*E*-value 1.6e-40) (Figure 4A) (for more detail on the promoter analysis see Supplementary Materials). A search for exact matches of the STRE binding site confirmed that, relative to all promoters in *S. cerevisiae*, PKA targets in *S. cerevisiae* were enriched for promoters that contained one or more STREs (76.2 vs. 44.8%, *p*-value 1.8e-17). They also had a notable increase in the average number of STREs per promoter (1.35 vs. 0.63) (Figure 4B, upper left). This is consistent with previous findings, implicating Msn2 and Msn4 as prominent targets downstream of PKA in *S. cerevisiae* (Smith, 1998; Görner et al., 2002).

Probing the bioinformatic characteristics of promoters of genes that are responsive to PKA inhibition in *K. lactis* revealed that those also had more STREs on average compared to the promoters of all genes in that species, however, the strength of the signal was lower than in *S. cerevisiae*. First, the top motif we identified in an unbiased search for short motifs resembled a Post Diauxic Shift (PDS) motif [ATCCCT(T/A)A] (Pedruzzi et al., 2000) as well as an STRE (Figure 4A). Furthermore, a search for exact matches to the STRE in PKA targets in *K. lactis* revealed a weaker enrichment than in *S. cerevisiae* (46.8% of promoters with 1 or more STRE in the promoter in PKA activated genes vs. 34.5% in all genes, *p* = 1.5e-4) (Figure 4B, upper right).

Taken together, these results indicate that the presence of STRE motifs in promoters might be a useful proxy for activation by PKA inhibition across budding yeast species. We therefore looked for these features in the promoters of DE_PKA_ ohnologs and their *K. lactis* orthologs to see whether the presence of STRE motifs correlated with the response to PKA we observed in our experiments. The distributions for the number and location of STREs for the DE_PKA_ high-LFC ohnologs closely resembled those of the promoters for genes activated by PKA inhibition genes (Figure 4B lower left, Supplementary Figure 13A). However, the STRE distribution for the DE_PKA_ low-LFC ohnologs resembled that of promoters for all genes in *S. cerevisiae*, consistent with the fact that they were not activated in response to PKA inhibition. Furthermore, the distribution of the number of STREs in the promoters of *K. lactis* orthologs of the DE_PKA_ genes was close to that of all genes in *K. lactis* (35.8 vs. 34.5% had one or more STRE in the promoter), consistent with the fact that these genes also had low LFC in response to PKA inhibition (Figure 4B lower right).

Because a motif resembling the TATA box [TATA(A/T)A(A/T)(A/G)] was present in genes induced by PKA inhibition in S. cerevisiae (Figure 4A), and because TATA boxes are known to be enriched in the promoters of stress responsive genes (Basehoar et al., 2004), we conducted a similar analysis in promoters of DE_PKA_ ohnologs and their *K. lactis* orthologs (Supplementary Figures 16–18). As in the case of the STRE, the number of TATA-boxes in the promoters of the DE_PKA_ high-LFC ohnologs were increased relative to those of the promoters of all genes (Supplementary Figure 17A). However, unlike with STREs, this enrichment was also present for DE_PKA_ low-LFC ohnologs and the *K. lactis* orthologs of the DE_PKA_ genes (Supplementary Figures 17A,B). Based on that observation, we reasoned that, at least in the context of the DE_PKA_ genes and their orthologs, the TATA box was not linked strongly enough to induction following PKA inhibition and was likely to be an ambiguous evolutionary signal. We therefore focused on the presence of STREs as a bioinformatic proxy for gene induction in response to PKA inhibition.

### Analysis of STREs in the Promoters of DE_PKA_ Orthologs Across Species Revealed Enrichment of STREs in the ZT Branch

To investigate whether the relationship between activation by PKA inhibition and STRE presence in the promoter holds in other budding yeast species, we revisited the cross-species gene expression datasets (Roy et al., 2013; Thompson et al., 2013) and scored STRE enrichment in the promoters of genes activated under conditions correlated with PKA inhibition in *S. cerevisiae* and *K. lactis* (Figure 4C). With the exception of heat stress in *K. lactis* and diauxic shift in *L. waltii* and *L. kluyverii*, the promoters of the genes activated under these stresses had more STREs than all genes in that species (*p* < 0.05 using Fisher's Exact test) although the overall background level of STREs and the level of enrichment varied between species.

These results suggest that there is a relationship between the presence of STREs in the promoter and gene induction under PKA inhibition that can be detected bioinformatically across budding yeast. We therefore quantified STREs in the promoters for orthologs of DE_PKA_ genes to search for examples in which the STRE conservation patterns might provide some insight into the evolution of gene responsiveness under conditions of PKA inhibition. We analyzed promoters in 32 non-WGH and 12 post WGH species, including species for which no stress or PKA inhibition gene expression data exists (e.g., species in the ZT branch). For this analysis, we only focused on the DE_PKA_ ohnolog pairs in *S. cerevisiae* that had at least one STRE in the promoter of the high-LFC member. We discarded genes with high-LFC but no STRE in the promoter, reasoning that they are induced through a mechanism that doesn't require the STRE (for example, not via Msn2/4), and thus the conservation of the STRE would not be the most relevant bioinformatic signal. We also removed pairs that had more than one ortholog in more than 8 non-WGH species which included ohnolog pairs that were present as duplicates before the WGH (such as the hexose transporters) and genes that may have undergone a separate ancient duplication which would complicate our analysis (such as *SNF3*/*RGT2*). Finally, we removed pairs that had no orthologs in non-WGH species (such as *USV1*/*RGM1*) (see Materials and Methods).

**Figure 5:**
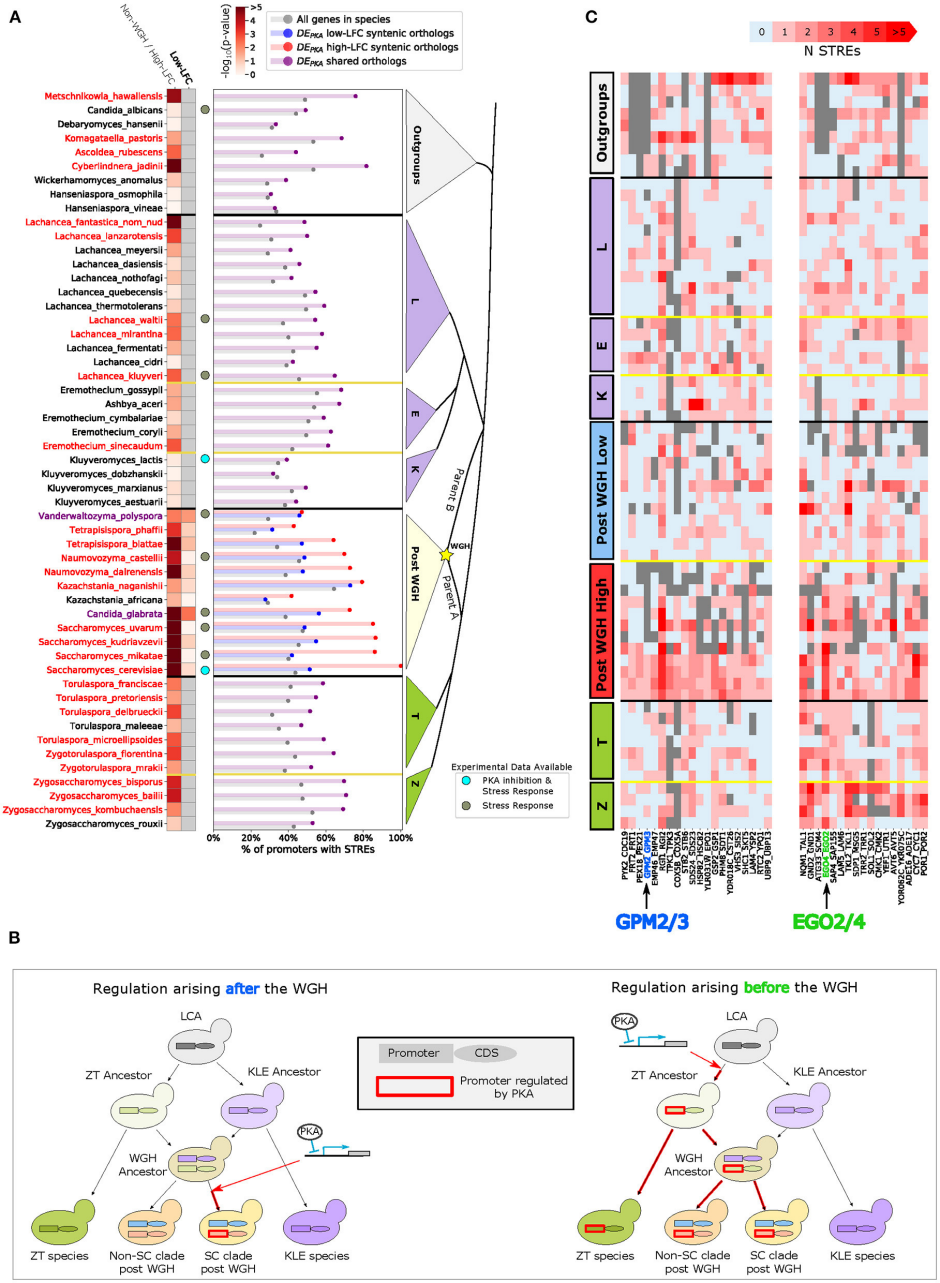
There is enrichment for STREs in the promoters of the syntenic orthologs of the DE_PKA_ high-LFC orthologs, as well as in the ZT branch, indicating that STREs arose in the promoters of DE_PKA_ high-LFC orthologs both after and prior to the WGH. **(A)** The barplot shows the percentage of promoters with 1 or more STREs in all genes in a species (gray) or in a subset consisting of the orthologs of DE_PKA_ genes (purple for non-WGH species and blue and red for syntenic orthologs of the low and high-LFC ohnologs, respectively). For this analysis we only include ohnolog pairs which had at least one ortholog in the non-WGH species analyzed, had no more than 8 species with duplicates in non-WGH species, and had at least one STRE in the high-LFC ohnolog in *S. cerevisiae* (74 ohnolog pairs). The heatmap shows the *p*-value (Fisher's exact test) of the hypothesis that the percentage of promoters with at least one STRE in a given set is different from that percentage for all genes in that species. For non-WGH species, species names are colored red when they meet the False Discovery Rate threshold of 5%. For post-WGH species, species names are colored red when the syntenic orthologs of the DE_PKA_ high-LFC ohnologs meets the FDR threshold. *C. glabrata* and *V. polyspora* have a purple label indicating that the syntenic orthologs of both the DE_PKA_ high and low-LFC ohnologs meet the FDR threshold. Gray dots next to a species indicate that gene expression data is available for stress conditions, and light blue dots are beside *S. cerevisiae* and *K. lactis* which have data for PKA inhibition as well as for stress conditions. **(B)** Schematic depicting scenarios for the appearance of induction in response to PKA inhibition both before (left) and after (right) the WGH. Orthologous promoter regions and coding sequences are depicted with rectangles and ovals, respectively. A red outline on a promoter indicates that it is induced by PKA inhibition. Red arrows indicate the point at which at least one orthologous protein in a species began being induced by PKA inhibition. **(C)** Number of STREs in the promoter for two clusters of ohnolog pairs in various species. Gray indicates that no ortholog was found. Data from non-WGH species and outgroups were used as input to the hierarchical clustering algorithm (see Materials and Methods).

This amounted to a total of 74 remaining ohnolog pairs. Exploration of the promoters of the orthologs of the high-LFC ohnologs in this set showed an enrichment of STREs in most post WGH species (Figure 5A barplot, red bars). This enrichment was not present in the low-LFC ohnologs (Figure 5A barplot, blue bars). Enrichment in high-LFC ohnologs was most prominent for species more closely related to *S. cerevisiae*. *C. glabrata* and *V. polyspora* were exceptions to this pattern with enrichment for STREs in both the syntenic orthologs of the low-LFC and high-LFC ohnologs. This pattern is consistent with STREs arising in the promoter of the ancestor of several high-LFC ohnologs following the WGH (Figure 5B, left).

Further analysis of the promoters of the non-WGH orthologs of this subset of DE_PKA_ ohnolog pairs revealed more enrichment for STREs in the ZT branch (9 out of 11 species) than in the KLE branch (only 6 out of 21 species) (Figure 5 heatmap). In the outgroups, there was a mixed picture, with 4 out of 9 species showing enrichment for STREs. Despite the fact that these data reflected the average of 74 separate sets of orthologs with their own idiosyncratic evolutionary histories, the evidence hints that some of the differential expression we see in DE_PKA_ ohnolog pairs may have arisen in the promoters of non-WGH orthologs in the ZT branch prior to the WGH (Figure 5B, right).

Taken together this evidence paints a picture in which the STRE, which drives gene expression in response to PKA inhibition via Msn2, seems to have arisen either before or after the WGH in the promoters of the ancestors of differentially expressed ohnologs (Figure 5B).

### *GPM2* and *GPM3* Illustrate a Case in Which STREs Might Have Been Added Following the WGH in the *Saccharomyces* Genus

We next explored potential specific examples in which the STRE arose either before or after the WGH in differentially expressed ohnologs. To identify specific promoters with a clear evolutionary signal, we clustered the subset of 74 DE_PKA_ ohnolog pairs described above based on the numbers of STREs in the promoters of their orthologs across 32 ZT and KLE branch species and 9 outgroups (Supplementary Figure 19). As expected, there is much variation given that the STRE is only a partial surrogate of PKA dependence. However, a few clusters highlight possible examples of WGH paralogs acquiring differential induction under PKA inhibition via distinct evolutionary trajectories. Specifically, one cluster of 20 ohnolog pairs was characterized by low numbers of STREs in the promoters of orthologs from both the ZT branch and the KLE branch (Figure 5C, left). This pattern is expected if the STRE and ensuing differential expression arose in the ancestor of the high-LFC ohnolog following the WGH (Figure 5B, left).

**Figure 6:**
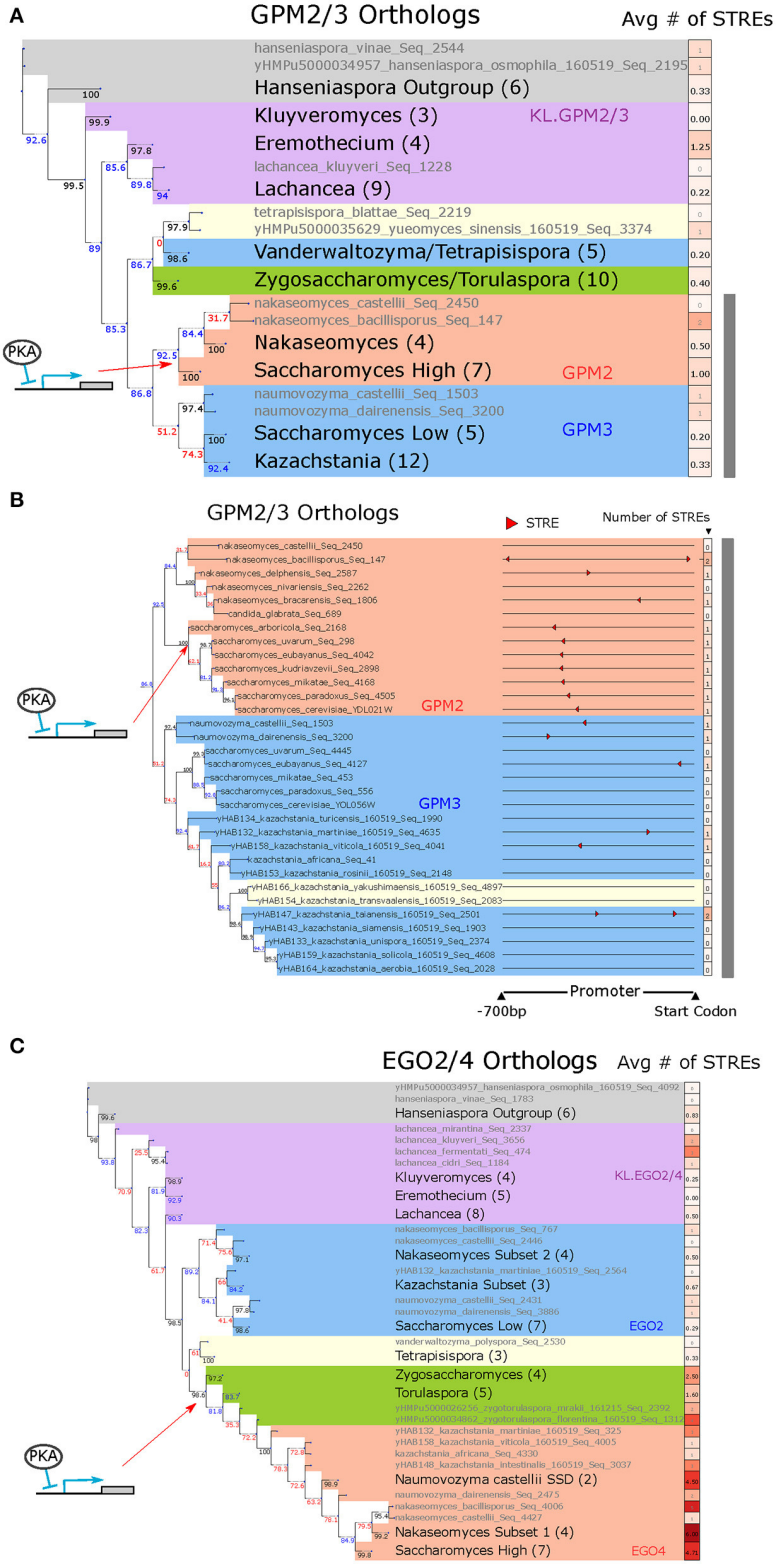
GPM2/3 are an example of a differentially induced ohnologs pair in which the STRE arose in the promoter of the DE_PKA_ high-LFC ohnolog following the WGH. EGO2/4 are an example of a differentially induced ohnolog pair in which the STRE may have arisen in the ZT branch prior to the WGH. **(A)** Phylogeny of GPM2/3 orthologs alongside average number of STREs in the promoter. Gray text indicates a single ortholog, and black text indicates a group of orthologs with the number of orthologs in that group in parentheses. The putative point at which the STRE was gained is indicated by the red arrow. Bootstrap support values (see Materials and Methods) for each branch point are written to the left of the branch point below the branch and colored black if the support value was above 95, blue if it was between 80 and 95, and red if it was below 80. Promoters are defined as 700 base pairs upstream of the start codon. Shading represents different groups of species; blue = post-WGH, syntenic ortholog to low-LFC ohnolog; red = post-WGH, syntenic orthologs to high-LFC ohnolog; yellow = post-WGH, synteny not determined; green = non-WGH ZT species; purple = non-WGH KLE species; gray = outgroups. The gray bar indicates the set of species detailed in **(B)**. **(B)** Phylogeny of GPM2/3 orthologs in the species most closely related to *S. cerevisiae* and visualizing their promoters (700 bp upstream of the start codon) with STRE motifs highlighted (red triangles). The red arrow indicates the putative point at which the STRE was gained. The first column of boxes after each promoter represents the number of STREs. Phylogeny and shading as in **(B)**. **(C)** Phylogeny of EGO2/4 orthologs alongside average number of STREs in the promoter. All conventions and nomenclature are as in **(A)**.

*GPM2/3*, which was present in that cluster, provides an example of this situation (Figures 6A,B, Supplementary Figures 19, 20A). *GPM2* and *GPM3* were identified in yeast as homologs to the phosphoglycerate mutase enzyme, *GPM1*, which is part of the glycolysis and gluconeogenesis pathways. *GPM2* has been shown to be important for growth under the respiratory carbon sources glycerol and sorbitol, and seems to play a role in the maintenance of cellular membrane stability (Gsell et al., 2013). A specific function for *GPM3* has not yet been identified. In our dataset, *GPM2* mRNA levels increase substantially under PKA inhibition (5.18 ± 0.47 LFC), while *GPM3* maintains a stable (albeit low) expression level (−0.24 ± 0.87 LFC) (Supplementary Figure 20B).

To explore the evolutionary history of STRE binding sites in *GPM2* and *GPM3*, we used protein sequence similarity to construct a phylogenetic tree for orthologous yeast proteins and traced binding sites in promoters associated with their coding sequences. We also used syntenic arrangement of the genes surrounding each paralog to identify whether the genomic context for each of the post-WGH orthologs was more similar to *GPM2* or *GPM3* (red or blue background, respectively, in Figures 6A,B, Supplementary Figure 20A, assigned as per Supplementary Table 8). While most non-WGH species contained a shared ortholog for *GPM2/3*, following the WGH, two orthologs (ohnologs) were only retained in the *Saccharomyces* genus. *GPM3* was more closely related syntenically and in terms of protein similarity with orthologs from the *Kazachstania* and *Naumovozyma* genus, while *GPM2* was more closely related to orthologs from the *Nakaseomyces* genus (including the ortholog of *Candida glabrata*).

In every syntenic ortholog of *GPM2* analyzed from the *Saccharomyces* genus there is a single STRE between 480 and 510 bases upstream of the gene's start codon (Figure 6B, Supplementary Figure 20B). While there are STREs in 3/6 of the closely related *Nakaseomyces* genus orthologs, their locations are not conserved. Thus, a single STRE binding site appears to have arisen in the promoter sequence of the common ancestor of the *GPM2* orthologs from the *Saccharomyces* genus, and this STRE binding site appears to have been conserved in the promoters of each of the modern *GPM2* orthologs in those species. This motif was presumably part of what gave rise to differential expression between the two paralogs in that clade. Although we cannot be sure that the presence of this STRE causes responsiveness to PKA in all these species without experimental data, the fact that the presence of the STRE corresponds to increased normalized LFC in the PKA related stress conditions in the species for which there was experimental data increases our confidence in this interpretation of the data (Supplementary Figure 21A).

### EGO2/4 Provides an Example Where Gain of STREs in the ZT Branch May Have Given Rise to PKA Dependence That Was Conserved in Syntenic Orthologs Following the WGH

Another cluster of 17 ohnolog pairs is characterized by a high number of STREs in the ZT branch and relatively fewer STREs in the KLE branch (Figure 5C, right). This pattern would be expected if the motif arose in the ZT branch after the two branches split but before the WGH (Figure 5B, right). EGO2 and EGO4 provide a clear illustrative example of this evolutionary trajectory (Figure 6C, Supplementary Figure 22A).

EGO2 and EGO4 in *S. cerevisiae* are short proteins (75 and 98 residues, respectively), that have no clear orthologs outside of the *Saccharomycetacea*, a group which includes the KLE, ZT and post-WGH yeast species. EGO2 was recently shown to be part of the EGO complex which is important for recruiting Gtr GTPases to the vacuolar membrane for TORC1 signaling (Powis et al., 2015). The biological function of EGO4 and its possible role in TORC1 signaling remain unclear. EGO2 is expressed at moderate levels (6.10 ± 0.13 rlog counts) during exponential growth and does not appreciably change under PKA inhibition (0.44 ± 0.58 LFC) (Supplementary Figure 22B). EGO4 has low expression during exponential growth (4.09 ± 0.04 rlog) and is highly induced (7.20 ± 0.45 LFC) under PKA inhibition. EGO4 is also an Msn2/4 target because its activation following PKA inhibition in an Msn2/4 deletion strain decreases substantially (to 1.47 ± 0.47 LFC) (Supplementary Figure 23).

We constructed a phylogenetic tree for EGO2 and EGO4, as we did for GPM2/3, using protein sequence similarity to define the structure of the tree and indicated syntenic relationships for post-WGH orthologs with shading (Figure 6C, Supplementary Figure 22A, assigned as per Supplementary Table 8). The structure of the tree suggested that the protein sequence likely diverged after the ZT and KLE branch split and prior to the WGH, because one branch of post-WGH ohnologs (the one containing EGO4) was more closely related to orthologs in the ZT branch than it was to the other branch of post-WGH ohnologs (the one containing EGO2) (Figure 6C).

There were more STREs on average in the promoters of ZT branch orthologs of EGO2/4 (Figure 6C), and all promoters for ZT branch orthologs had at least 1 STRE within 700 bases of the transcription start site, and a TATA box within 300 bp of the transcription start site (Supplementary Figure 22). The location of these motifs was in some cases conserved across species. This pattern was preserved in the promoters of the post-WGH syntenic orthologs of EGO4 and was not generally present in the promoters of the post-WGH syntenic orthologs of EGO2. In the promoters of the KLE orthologs, there were fewer STRE and TATA box motifs and those that were present were scattered more sporadically (Figure 6C, Supplementary Figure 22). Again, we cannot be certain that the presence of STREs in these promoters corresponds to increased responsiveness without experimental data, but the fact that normalized LFC in the PKA related stress conditions is higher when more STREs are present indicates that the STRE at least plays an important role (Supplementary Figure 21B). The phylogenetic distribution of this pattern paints a picture in which STREs arose in the promoter of the ancestral EGO2/4 ortholog in the ZT branch before the WGH parental strain (parent A in Figure 1A) separated and were then conserved. The promoter of EGO4, which descended from this lineage, contains some of these conserved STREs. This presumably rendered EGO4 inducible by PKA inhibition (Figure 5B right). In this scenario, the promoter for EGO2 would have descended from the WGH parental strain more closely related to the KLE branch (parent B in Figure 1A), which would have had no STREs and would not have been induced by PKA inhibition.

## Discussion

Gene duplication is a major driver of innovation in evolution, and our findings from this study help to better understand the mechanisms that can drive this evolutionary innovation in the special case of an allopolyploidization or Whole Genome Hybridization (WGH). One of the most well-studied examples of a WGH occurred in budding yeast in the branch leading to the canonical single-celled eukaryote, *S. cerevisiae*. This WGH has been implicated as a key factor that enabled the change in metabolic lifestyle from respiratory (or Crabtree negative) to respiro-fermentative (Crabtree positive) that occurred at the same time as the WGH in that lineage (Merico et al., 2007). It is known that the paralogs generated from the yeast WGH (ohnologs) are often differentially expressed under stress conditions (Gasch et al., 2000; Gasch, 2002; Thompson et al., 2013). However, the pleiotropic nature of environmental stress makes it difficult to parse the contribution of different interacting stress response pathways to differential expression. By narrowing our initial experiments to the PKA pathway, a conserved master regulator of the Environmental Stress Response (ESR), and, taking advantage of ATP analog-sensitive alleles to specifically perturb the pathway, we could meaningfully explore how the signal from PKA was delivered to ohnologs that are differentially expressed. Additionally, in comparing the transcriptional response to PKA inhibition between the non-WGH species *K. lactis* and the post-WGH species *S. cerevisiae*, direct control of PKA ensured that changes we observed were the result of changes downstream of this particular pathway rather than upstream changes in the stress sensing machinery or crosstalk between pathways.

Our data established a strong enrichment for ohnologs from the yeast WGH in the set of genes activated by PKA inhibition in *S. cerevisiae* and revealed that many of these ohnologs had differential expression, with one member of the pair showing high induction by PKA inhibition and the other remaining insensitive to PKA. This set amounts to a large proportion (almost 1/6th) of the retained ohnologs in *S. cerevisiae*. Our comparison with the transcriptome of *K. lactis* in response to PKA inhibition, and our further analysis of publicly available gene expression data from a number of budding yeast species (Tsankov et al., 2010; Roy et al., 2013; Thompson et al., 2013) in response to stress conditions that approximated PKA inhibition revealed that the orthologs of these differentially expressed ohnologs in various non-WGH species had low induction and moderate basal expression. These data suggested that insensitivity to PKA is likely to be the ancestral state for many of these differentially expressed ohnologs. The fact that activation in response to PKA inhibition is likely to be the derived state in many differentially expressed ohnolog pairs, and that the differentially expressed ohnolog pairs were enriched for metabolic enzymes implies that the WGH provided an opportunity for various metabolic pathways to incorporate signals from the PKA pathway, and to develop paralogous enzymes that were specialized for conditions under which PKA is inhibited. It is tempting to speculate that the differential expression of this specific set of paralogs in response to PKA inhibition in *S. cerevisiae* has contributed to the evolution of the respiro-fermentative lifestyle of *S. cerevisiae* and other post-WGH species. A largely non-overlapping subset of paralogs are differentially expressed under PKA-related stresses in the post-WGH species *V. polyspora* (Supplementary Figures 13C, 15B), which, unlike *S. cerevisiae* and its closest relatives, does not perform glucose repression for metabolism of other carbon sources which is part of why *S. cerevisiae* is such a strong fermenter (Hagman and Piškur, 2015). Perhaps the differential expression of those paralogs contributes instead to other metabolic phenotypes in *V. polyspora*.

To explore the evolutionary timeline for the emergence of PKA dependence for the ohnologs that were induced by PKA inhibition, we looked for bioinformatic signals in the promoters of differentially expressed ohnologs and their orthologs across over 70 sequenced budding yeast genomes. We found compelling evidence that the STRE binding site, which is strongly linked to activation by Msn2/4 under PKA inhibition, was gained in the ZT branch prior to the WGH in some cases and arose following the WGH in others. The STRE motif is a bioinformatic signal with clear weaknesses, including a relatively low information content (just 5 bases) and in imperfect correlation between the signal (the STRE) and the biological phenotype it was meant to detect (induction in response to PKA inhibition). Despite this, we were still able to leverage the high density of sequenced species in budding yeast to draw evolutionary conclusions (Shen et al., 2018), illustrating the usefulness of the budding yeast as a model phylum for studying evolution (Botstein and Fink, 2011). The examples of gene promoters we further analyzed as examples of these scenarios highlighted additional interesting evolutionary issues.

Given the immense challenge faced by natural selection to maintain redundancy despite mutations, understanding how duplicated genes persist is one of the important questions in understanding evolution. Many hypotheses have been put forward, including the “balance hypothesis” (Papp et al., 2003), which posits that paralogous genes that are dosage dependent (i.e., members of essential complexes) are likely to be retained following a WGD or WGH, since the loss of one copy would cause a substantial fitness defect. This is likely to be the case for the large contingent of ribosomal ohnolog pairs that are simultaneously repressed under PKA inhibition in our data. Other evolutionary forces that work to generate functional divergence in paralogs include escape from adaptive conflict, sub-functionalization, and neofunctionalization (Hittinger and Carroll, 2007; Des Marais and Rausher, 2008). In an autopolyploidization event, or WGD, however, one still must explain how such mechanisms are deployed. Because the two copies of a gene are initially identical in a WGD, one must invoke either very strong selection for or easily accessible mutations which provide advantageous functional divergence for the divergence to arise before deleterious mutations have a chance to eliminate one of the two identical paralogs.

An allopolyploidization, or WGH, on the other hand provides a simpler explanation for the origin of functional divergence in paralogous genes. Two paralogous genes might have functionally diverged during the time they were separated in different lineages, each adapting to its own specific environment. We envision this to be the case for EGO2/4 genes, which arrived in the hybrid WGH ancestor with differential regulation (one controlled by PKA and one not) and divergent sequences as reflected by a protein phylogeny in which the ohnologs are more similar to their descendants of their respective ancestor from the WGH (KLE or ZT branch) than to one another. GPM2/3 may also have experienced functional divergence prior to the WGH, but it appears that differential regulation in response to PKA (in the form of STREs in the promoter) did not arise until afterwards. The idea that genes from interspecies hybrids in yeast might maintain preexisting regulatory differences present in parental species is supported by recent work showing that most transcriptional differences between homologous genes in a newly formed *S. cerevisiae* and *S. uvarum* hybrid preexisted in the parental species (Hovhannisyan et al., 2020).

This simple story, however, is often greatly complicated by the occurrence of gene conversion following WGH events when homologous genes overwrite all or part of their paralogs either soon after the polyploidization event or in the following millennia (Louis et al., 2012). In our example of EGO2/4 the syntenic relationships of post-WGH orthologs corresponded to their phylogenetic relationships based on protein sequence similarity, but such correspondence is not the case when a gene conversion has occurred. Even when a gene conversion has occurred, differential expression and functional differentiation between ohnologs can occur as is the case for the functionally divergent ohnolog pair GDH1 and GDH3 (Campero-Basaldua et al., 2017). Because gene conversions are driven by homology, and the tract length of gene conversions in yeast is typically between 50 and several hundred base pairs long (Mansai et al., 2011), it is possible that cis-regulatory regions, whose sequences are less conserved than protein coding regions of the genome, would not be involved in a gene conversion. It is therefore likely that following a WGH, the protein coding region of an ohnolog pair could have been homogenized through gene conversion while the regulatory regions retained the properties of the respective parent species. In this way, identical proteins could be expressed in different conditions and, over time, evolve specialization for those conditions. Identifying such an event will require sophisticated bioinformatic analysis that leverages a more nuanced understanding of the relationship between cis-regulatory sequence and gene expression.

Recent advances which illuminate this genotype to regulatory phenotype relationship in *S. cerevisiae* (Bashor et al., 2019; Brodsky et al., 2020; de Boer et al., 2020), combined with the high phylogenetic density of sequenced genomes make the budding yeast clade a fertile ground for developing our understanding of how gene regulation evolves. Our results, demonstrating different ways that differential gene expression can arise in paralogs in response to signals from the PKA master regulatory pathway, serve as a useful contribution to that important quest.

## Materials and Methods

### Plasmid and Strain Construction

All plasmids and strains used in this study are listed in Supplementary Tables 6, 7.

The *S. cerevisiae* PKA-AS base strain (yBMH33) was obtained from from of Nan Hao and Erin O'Shea (Hao and O'Shea, 2011). In addition to the gatekeeper point mutations (M164G, M147G, and M165G for TPK1, TPK2, and TPK3, respectively), the strain contained an NHP6A-IRFP nuclear marker which was not used in this study.

The *K. lactis* PKA-AS strains (yBMH132, yBMH078), containing the M139G and M222G mutations for KLLA0D03190 (KL.TPK2) and KLLA0B07205 (KL.TPK3) respectively, were constructed using a single plasmid CRISPR strategy based on (Ryan and Cate, 2014). Cas9 and sgRNA expression constructs were combined using Gibson Assembly on a backbone with a *K. lactis* autonomously replicating sequence that allows plasmid replication in a variety of budding yeast species (Liachko and Dunham, 2014). The guide targeting sequence was changed using Gibson assembly to combine PCR products containing a new guide sequence with the digested backbone. Donor constructs had at least 300 bp of homology upstream and downstream from the point mutation, as well as a heterology block consisting of synonymous mutations in the location of the sgRNA target to prevent re-cutting by the Cas9/sgRNA complex as described in Horwitz et al. (2015). The donor cassette was printed by SGI-DNA, inc. and integrated into a PUCGA 1.0 backbone.

Lithium acetate protocols were used for *K. lactis* CRISPR/Cas9 gene editing and for strain construction in *S. cerevisiae*. Electroporation protocols were used to generate *K. lactis* strains containing KL.Msn2 nuclear localization markers. Details on transformation protocols for each strain used in the study are contained in the Supplemental Material.

### Growth Experiments

Yeast were picked from freshly streaked plates and grown overnight in YPD to saturation. In the morning they were diluted to OD 0.2 in deep well-plates shaking at 900 rpm and 30°C in an INFORS-HT Multitron shaker for 1.5 h. One hundred microliters of cells were then combined with 100 μl 2× perturbation media containing 1-NM-PP1 (for a final concentration of 3 μM) or DMSO in a 96 well-black polystyrene microplate (Corning 3904). OD600 measurements were collected using 10 flashes and a settle time of 100 ms on a TECAN SPARK10M plate reader every 20 min using Spark Control V2.2 software. A humidity cassette was used with no lid on the plate. Temperature control was set to 30°C, and the plate was set for continuous double orbital shaking (2.5 mm, 108 rpm). Growth data was analyzed using custom python scripts located at https://github.com/heineike02/PlateReaderTools.

### Microscopy

Yeast were grown overnight to saturation in 4 ml SDC and diluted to approx. OD 0.05 in the morning to get OD 0.2 after 5 h (6.5 h for K. Lactis) assuming a lag time of 90 min for both species and a doubling time of 115 for *S. cerevisiae* and 130 min for *K. lactis*. Ninety six well glass-bottom plates (Brooks Life Science Systems MGB096-1-2-LG-L) were prepared for imaging by coating with 0.25 mg/ml concanavalin A (Sigma-Aldrich C2010, resuspended in distilled water) (ConA) for 30 min and washed twice with SDC prior to adding cells. Cells were sonicated gently (amplitude 1 for 3s using a Misonix S-4000 sonicator with a 1/16in microtip P/N #418) to separate clumps and incubated in appropriate wells for 30 min. Following incubation, cells were washed twice with 100 μl SDC leaving 100 μl of SDC in the well-above immobilized yeast cells. After 2–3 initial images 100 μl of 2× perturbation media (SDC plus 1-NM-PP1 for a final concentration of 4 μM, or a DMSO control) was added to the plate. Cells were then imaged every 2–3 min until the end of the experiment.

Widefield microscopy images were taken on a Nikon Ti-E inverted scope, with an incubation enclosure set to 30°C, and mercury arc-lamp illumination using RFP filters and dichroic mirror from the Chroma 89006 ET-ECFP/EYFP/mCherry filter set. The microscope's perfect focus system was used to maintain focus on the samples throughout the experiment. The microscope was controlled with micro-manager software version 1.4.17 using the High Content Screening Site Generator Plugin (Edelstein et al., 2014). Images were taken with an Andor 512 pixel EMCCD camera (897 iAxone DU-897E) using a 40× objective (Nikon Planfluor 40× /0.75) and 1.5× optical zoom.

Image analysis was conducted using custom MATLAB scripts located at https://github.com/heineike02/image_analysis. Briefly, RFP images are background subtracted, smoothed, and cells are identified using the Lucy-Richardson deblurring algorithm (MATLAB function deconvlucy) with 5 iterations based on an image of a single typical cell of the appropriate species. For each identified cell, nuclear localization is calculated as the mean intensity of the top 5 brightest pixels to the median pixel intensity.

### RNA Sequencing

Cells were grown overnight at 30°C in YPD to saturation, and then diluted to obtain a 30 ml of cells with an OD600 of 0.5 in 4–6 h assuming a lag time of 90 min and a doubling time of 90 min for *S. cerevisiae* and 110 min for *K. lactis*. The culture was then divided into two 12 ml treatment and control cultures and 3.6 μl of 10 mM 1-NM-PP1 (for a final concentration of 3 μM) or DMSO was added, respectively. Each culture was incubated while shaking at 250RPM and 30°C and split into two 5 ml aliquots prior to spinning down at 3850RPM (331rcf) on an Eppendorf 5810R benchtop centerfuge for 3 min. After spinning down, supernatants were poured out and remaining supernatant was aspirated off with a P1000 pipette. Cells were then flash frozen in liquid nitrogen and stored at −80°C.

RNA extraction was performed using the hot acid-phenol extraction protocol of Solís et al. (2016) with the following changes. Initial cell volume was 5 ml instead of 1.5 ml and thus the initial spin for collecting the cells was done for 3 min at 3850RPM (331rcf) instead of 30s at 13000RPM (15871rcf). Acid-phenol:chloroform:isoamyl alcohol (IAA) (125:24:1), pH 4.5 (AM9722) was used instead of pure Acid-Phenol because the chloroform aids in the separation of nucleic acid from proteins and lipids, and the IAA prevents foaming. The spin following heat incubation was performed at room temperature instead of at 4°C. A second 400 μl chloroform wash was included prior to removing the aqueous phase from the phase lock tubes. Twenty two microliter of 3 M NaOAc, pH5.2 was added instead of 30 μl to precipitate the DNA. Ethanol Precipitation was done overnight instead of for 30 min.

Total RNA was aliquoted and stored at −80°C. Prior to library preparation, total RNA concentration was measured estimated using a Nanodrop 2000c spectrophotometer and screened for quality by electrophoresis. For electrophoresis, a formamide based loading dye was used to run samples on a 1.2% agarose gel in TBE buffer. Select samples were also checked for quality using an Agilent 2100 Bioanalyzer with an RNA 6000 Pico chip. 3′ Sequencing Libraries were prepared using the Lexogen QuantSeq 3′mRNA-Seq Library Prep kit FWD using dual indices for each sample. Select libraries were checked for quality on the Bioanalyzer using a High Sensitivity DNA chip. Library concentrations were calculated using a Qbit 2.0 Fluorometer (Invitrogen), and 2.65 ng per sample were pooled and sequenced on an Illumina Hiseq 4000 sequencer to an average depth of between 245,000 and 5.6 million reads per sample (median 2.53 million). At least 3 replicates were collected for each sample and condition.

Sequencing data was trimmed, aligned, and checked for quality using the Bluebee genomics analysis pipeline. *S. cerevisiae* samples were run using the “FWD S. cerevisiae (R64) Lexogen QuantSeq 2.2.3” protocol, and *K. lactis* samples were run using the “FWD K. lactis (ASM241v1) Lexogen Quantseq2.2.3” protocol. The counts generated with these pipelines used the saccharomyces_cerevisiae_R64-2-2_20170117 GFF for *S. cerevisiae*, and many of the 3′ sequencing reads fell into unannotated 3′UTR regions. We therefore created a modified GFF file which included 3′ UTRs from (Nagalakshmi et al., 2008). We had a similar issue for the *K. lactis* reads, but as there are no definitive studies annotating the *K. lactis* 3′UTR, we created a *K. lactis* GFF with 400 bp extensions for each annotated gene serving as estimated 3′UTR regions. We then recalculated gene counts for each species using htseq in intersection-non-empty mode. Custom scripts and the updated GFF files used for these calculations are available at https://github.com/heineike02/UTR_annotation and https://github.com/heineike02/rna_seq_processing.

Gene count data was processed to yield estimates for raw expression (rlog values) for all experiments as well as Log Fold Change and adjusted *p-*Value estimates for differential expression between conditions using the DESeq2 package in R (Love et al., 2014).

After filtering samples that failed quality control, our RNA-seq dataset contained 59 *S. cerevisiae* samples and 35 *K. lactis* samples which were sequenced in the same run. These samples included the WT +/– drug and AS +/– drug experiments as well as experiments with Msn2/4 deletion and Rph1/Gis1 deletion mutants. In each species, we kept only genes that had 2 or more counts in at least 3 samples for that species. The rlog was calculated using the rlog function with blind=FALSE, and an experimental design which combined the presence of the AS mutation, transcription factor deletion status and presence of drug into a single factor. Mean rlog data for each condition was calculated by averaging rlog values across replicates. To calculate distributions of rlog values for all genes in *S. cerevisiae*, 443 orfs classified as dubious in the saccharomyces_cerevisiae_R64-2-2_20170117 GFF were filtered out.

Log Fold Change and adjusted *p*-values for differential expression between samples was calculated from deseq using a False Discovery Rate of 1% for the relevant contrast (PKA-AS strains +/– drug at 50 min, or ΔMsn2/4 or ΔKL.Msn2 PKA-AS strains +/– drug at 50 min). Custom scripts for further RNA sequencing analysis and enrichment are located at https://github.com/heineike02/yeast_esr_expression_analysis

### GO Term Enrichment Analysis

The GO-slim database used for analysis was downloaded from SGD on 20180412 (SGD Project, 2018). For GO analysis of genes induced or repressed by PKA inhibition in both species, only Biological Process terms were analyzed. Go terms for *K. lactis* genes were assigned based on their orthologs in *S. cerevisiae*. If a *K. lactis* gene had two *S. cerevisiae* orthologs, both of those genes were included for the calculation. Fisher's exact test was used to test the hypothesis that the proportion of genes with a particular GO-slim term in a given set is greater than would be expected given the distribution of that term in a background set. After calculating *p*-values for all GO-slim terms, significance was assessed using a FDR of 5% based on the Benjamini/Hochberg (BH) procedure. The background set for the genes only activated or repressed in *S. cerevisiae* was all genes in *S. cerevisiae*. The background set for the sets of genes involving activation or repression in *K. lactis* was the set of all genes in *S. cerevisiae* which contain orthologs in *K.lactis*.

For GO analysis of DE_PKA_ genes, we analyzed Biological Process, Molecular Function, and Cellular Component terms for both enrichement and de-enrichment vs. either all genes for which we had RNA-seq data or against all ohnolog pairs for which we had data for both ohnologs (Supplementary Table 4). We analyzed the DE_PKA_ low and DE_PKA_ high genes separately. As a basis for comparison, we also calculated enrichment and de-enrichment of ohnologs vs. all genes with data. To cluster DE_PKA_ ohnolog pairs according to their GO annotation for Supplementary Figure 5, we used the python scipy.cluster.heirarchy.linkage function using the “ward” method. Each ohnolog pair was assigned a vector of ones or zeros indicating whether or not the GO-slim term was present in each ohnolog, and if the ohnolog pair was present in the yeast metabolic map from iPath 3.0, we also included an indicator with a value of 4. The higher value was used to force all ohnolog pairs on the metabolic map to cluster together. Euclidean distances between these vectors were used as input to the hierarchical clustering. For the GO-analysis, we picked terms that were enriched in DE_PKA_, an additional curated subset of terms present in multiple ohnolog pairs, and the terms “biological_process,” “molecular_function,” and “cellular_component” which represent genes with unknown annotations for the three aspects of the GO ontology.

To visualize genes on the metabolic map of yeast, we used iPath v3 via its web interface on 25 Feb, 2021 (https://pathways.embl.de/about.cgi) (Darzi et al., 2018).

### Ohnolog and Ortholog Assignment

The ohnolog dataset for *S. cerevisiae* was downloaded from the Yeast Genome Order Browser (YGOB) website on 10 October, 2017. Fisher's exact test was used to test the hypothesis that the proportion of ohnologs in a given set is greater than would be expected given the distribution of ohnologs in the background set. The same test sets and background sets were used as for GO term enrichment analysis.

Ohnolog sets for other species were identified using the “pillars” database downloaded from the Yeast Genome Order Browser on 08 September, 2016.

Orthology assignments between *S. cerevisiae* and *K. lactis* were generated using the YGOB pillars database.

We used orthology assignments from the fungal orthogroups (Wapinski et al., 2007)for comparisons of microarray data from Roy et al. (2013), Thompson et al. (2013) when present. That database contained no orthology assignments for *V. polymorpha*, so orthology assignments from *S. cerevisiae* to *V. polymorpha* were generated using YGOB pillars.

The gene names used for *K. lactis* and *N. castellii* on the fungal orthogroups website and in those microarrays was inconsistent with the standard gene names which are used by YGOB. We therefore generated a mapping for mismatched gene names between the two databases using protein similarity based on the genome sequences provided by each study. To generate a similarity score between candidate proteins, we used the pairwise2.align.globalms function from biopython with the following parameters: match_points=1, mismatch_points=-1, gap_open=-0.5, gap_extension = −0.1. We then took as candidates the top 5 genes above a threshold (*K. lactis*: 138, *N. castellii:* 115). We kept the top candidate and any lower scoring candidates as long as there was not a drop in 10 points between that candidate and the next highest scoring gene. We generated similar mappings going the opposite direction from the YGOB genename to the fungal orthogroup genename for *N. castellii, K. lactis*, and *S. mikatae* (threshold scores of 120, 138, and 130, respectively), and a mapping from the *K. lactis* standard gene name to the gene identification numbers assigned in Shen et al. (2018) using a threshold score of 110.

To identify syntenic orthologs of DE_PKA_ genes within post-WGH species to analyze their promoters, we used the YGOB webtool with a window of +/– 8 genes.

To determine orthology relationships for genes from Shen et al. (2018), we used orthogroups as defined by that paper's orthomcl.clusters.txt file.

To identify syntenic orthologs within the post-WGH species for our example ohnolog pairs (EGO2/4, and GPM2/3) we used YGOB to generate a syntenic alignment with a window of +/– 8 genes around each example gene. We extracted the surrounding genes from the sequenced genomes for all species and assigned orthology for the surrounding genes based on presence in the same orthogroup as *S. cerevisiae* and *K. lactis* genes. We then manually curated synteny based on the pattern of orthologs present (Supplementary Table 8). Linking promoter information from Shen et al. (2018) to gene expression data from Roy et al. (2013), Thompson et al. (2013) for Supplementary Figure 19 required some manual curation of orthology assignments, including a small scale duplication of the ortholog of EGO2/4 in *N. castellii* that was not captured in YGOB or the fungal orthogroups on which the data was based.

### Microarray Data Processing

Gene expression datasets from microarrays measuring the response to stress conditions in various budding yeast species from Thompson et al. (2013) (GSE36253) and (Roy et al., 2013) (GSE38478) were downloaded from the NCBI Gene Expression Omnibus (https://www.ncbi.nlm.nih.gov/geo). The data represented Log Fold Change of intensity for the experimental condition divided by the control condition. Where there were multiple datapoints for the same gene name, the median value was used. We took the median value of technical replicates. To compare data across species, we combined all data for all conditions for a given species into a single dataset, and then used the mean and standard deviation to center and normalize all data for that species.

Microarray data estimating raw expression by comparing mRNA from cells collected during exponential growth on rich media to genomic DNA from Tsankov et al. (2010) (GSE22193) were downloaded from the NCBI Gene Expression Omnibus (https://www.ncbi.nlm.nih.gov/geo). The median of all data that had more than one spot per gene name was used for each gene name. Data for replicates were quantile normalized (Bolstad et al., 2003) using the implementation of (https://github.com/ShawnLYU/Quantile_Normalize), and then the median of the replicates was used for each gene name. Before comparing data across species, the data were mean-centered and normalized by the standard deviation.

### Identification of PKA Targets and Differentially Expressed Ohnologs

Genes were classified as activated and repressed by PKA inhibition for GO term and Ohnolog enrichment analysis in both *S. cerevisiae* and *K. lactis* when LFC was >2.0 with a 1% FDR based on the Benjamini/Hochberg procedure.

In *S. cerevisiae* we identified differentially expressed ohnolog pairs by first classifying all ohnologs based on their LFC as either strongly repressed (LFC <-2.0 at 1% FDR), weakly repressed (−2.0 <LFC <-1.0 at 1% FDR), weakly activated (1.0 <LFC <2.0 at 1% FDR), strongly activated (LFC>2.0 at 1% FDR), or no change (all other ohnologs). Each ohnolog pair was then sorted to identify the ohnolog with the lower LFC and that with the higher LFC, except that when one ohnolog was significant according to the FDR threshold and the other was not, the significant ohnolog was assigned as the higher or lower ohnolog depending on whether the significant ohnolog had positive or negative LFC. For those ohnolog pairs in which both ohnologs did not meet the FDR threshold, lower and higher LFC ohnologs were also assigned. DE_PKA_, the set of ohnolog pairs that are differentially expressed under PKA inhibition in which one ohnolog is activated was defined by the set of ohnolog pairs in which the higher LFC ohnolog was strongly activated and the lower LFC ohnolog was repressed or had no change.

In other species, and for the set of genes differentially expressed under PKA related stress conditions, we identified differentially expressed genes by first defining an estimated Log Fold Change by averaging across the 3–5 (depending on the species) PKA inhibition related conditions (LFC_est_). As in the *S. cerevisiae* RNA-seq data, we first ordered the ohnologs so that Ohnolog 1 had a lower LFC than Ohnolog 2. We then identified ohnolog pairs in which Ohnolog 2 was activated (LFC_est_ > 1.5) and Ohnolog 1 was not (LFC_est_ <0.9). We also required that the difference in LFC_est_ between the ohnologs was greater than 0.9 (Supplementary Figure 12B). The maximum LFC_est_ cutoff of 0.9 for Ohnolog 1 was chosen because the size of the overlap between $DE_{Stress}^{Scer}$ and DE_PKA_ did not increase any further when it was increased (Supplementary Figure 12C right), and the minimum LFC_est_ cutoff was chosen because it yielded a $DE_{Stress}^{Scer}$ whose size was similar to DE_PKA_ (Supplementary Figure 12C left).

### Promoter Extraction

Promoter sequences were taken to be the 700 bp upstream of the start codon (except when the scaffold contained <700 bp in which case all bases were used). *S. cerevisiae* promoter sequences were taken from SGD. Promoter sequences for *K. lactis* were extracted gene by gene using NCBI E-utilities. Promoters for YGOB species used in motif analysis for genes that were targets of PKA related stress conditions (Figure 4C), as well as for counting STRE motifs in post-WGH species in Figures 5A,C were extracted from the genomes for those species on the YGOB website. Promoters for genes in non-WGH species in Figures 5A,C, and for all species in figure 6 were extracted from the genomes published in Shen et al. (2018) using custom scripts available at https://github.com/heineike02/y1000plus_tools.

To analyze promoters of the orthologs of DE_PKA_ genes in 32 non-WGH *Saccharomycetaceae* species and 9 outgroups, we first identified orthologs using orthogroups calculated in Shen et al. (2018). We removed any ohnolog pairs that did not contain any STREs in the high-LFC ohnolog reasoning that the high-LFC ohnolog in those pairs were induced through a mechanism that did not require the STRE. We also removed pairs that had that had no orthologs in non-WGH species (such as USV1/RGM1). Finally, we removed pairs that had more than one ortholog in more than 8 pre WGH species. This was to avoid ohnolog pairs that were present as duplicates before the WGH (such as the hexose transporters) and ohnolog pairs whose non-WGH orthologs may have undergone a duplication after the WGH, but still ancient enough to be present in several species (such as SNF3/RGT2). This left 74 of 110 DE_PKA_ ohnolog pairs.

### Motif Enrichment

*De novo* motif identification was conducted on indicated sets of promoters using the DREME algorithm from the MEME suite v5.1.1 (Bailey, 2011) with default promoters and a background set of all promoters from either *S. cerevisiae* or *K. lactis* that contained expression data in our dataset as appropriate.

STRE (CCCCT) and TATA box [TATA(A/T)A(A/T)(A/G)] motifs were identified by looking for exact matches within the promoters, and enrichment was calculated using Fisher's exact test.

### Clustering

In order to identify evolutionary trends in STRE appearance before the WGH, we clustered DE_PKA_ ohnolog pairs based on the number of STREs in the promoters of their orthologs in 32 non-WGH *Saccharomycetaceae* species and 9 outgroups. We created a distance matrix for the 60 ohnolog pairs by using the correlation distance between two rows with missing values removed. Rows were then hierarchically clustered (scipy.cluster.hierarchy) using the “average/UPGMA” linkage method. Separate clusters were defined using the fcluster method with the “inconsistent” parameter set to 1.1.

### Phylogenetic Trees

Orthologs for GPM2/3 and EGO2/4 were identified based on orthomcl clusters from Shen et al. (2018). For EGO2/4 there were three separate clusters so protein sequences for the clusters were combined for the analysis. A multiple sequence alignment was created using MAAFT version 7.407 (Katoh and Toh, 2008) using the E-INS-I algorithm (–genafpair) with –maxiterate 1000. These multiple sequence alignments were then trimmed with trimAL v1.4.rev22 (Capella-Gutiérrez et al., 2009) with the -gappyout option. Evolutionary models were identified as LG+F+I+G4 (for GPM2/3) and LG+I+G4 (for EGO2/4) using IQTREE model finder (Kalyaanamoorthy et al., 2017). The same model was selected in three independent runs of the algorithm with w-BIC values above 0.96 in each run. Trees were constructed using IQTREE v1.6.12 (Nguyen et al., 2015). Ten independent runs of IQTREE were conducted on the selected model for each trimmed multiple sequence alignment model using -bb 1,000 which calls the UFboot2 algorithm (Hoang et al., 2018) as well as -alrt 1000 to find support values. The tree with the best likelihood by BIC was chosen except when BIC values were equal in which case AIC and likelihood values were also used. For EGO2/4, because there were less variant alignment positions compared with the number of model parameters (77 for both E-INS-I and L-INS-I with 161 parameters to determine branch length and tree structure for 81 sequences), we also built trees using a multiple sequence alignment created with the mafft L-INS-I algorithm (–localpair) to see if the results were robust to variations in the multiple sequence alignment. The chosen model did not change depending on the alignment. The ordering of the clades for that tree did not change depending on the alignment, although the BIC was lower for the original E-INS-I alignment. Support values for EGO2/4 were calculated with the -bnni option to expand the search space for bootstrap trees.

## Data Availability Statement

The RNA sequencing data presented in this study can be found at NCBI GEO [accession: GSE163741]. Information about plasmids is at https://www.addgene.org/plasmids/articles/28210974/. Scripts can be found at https://github.com/heineike02 in the repositories listed in the Methods section. Other data can be found at: https://doi.org/10.6084/m9.figshare.14428763.v1.

## Author Contributions

BH and HE-S study conceptualization, experiment design, and wrote the paper. BH: experiments and bioinformatic analysis. HE-S supervision and funding. Both authors contributed to the article and approved the submitted version.

## Conflict of Interest

The authors declare that the research was conducted in the absence of any commercial or financial relationships that could be construed as a potential conflict of interest.
