# Supplementary material for: Paralogs in the PKA Regulon Traveled Different Evolutionary Routes to Divergent Expression in Budding Yeast
Source: Front Fungal Biol. 2021 Apr 27;2:642336. doi: 10.3389/ffunb.2021.642336 (PMC10512328; doi:10.3389/ffunb.2021.642336)
Supplement: Supplementary file 1 [file Data_Sheet_1.pdf]

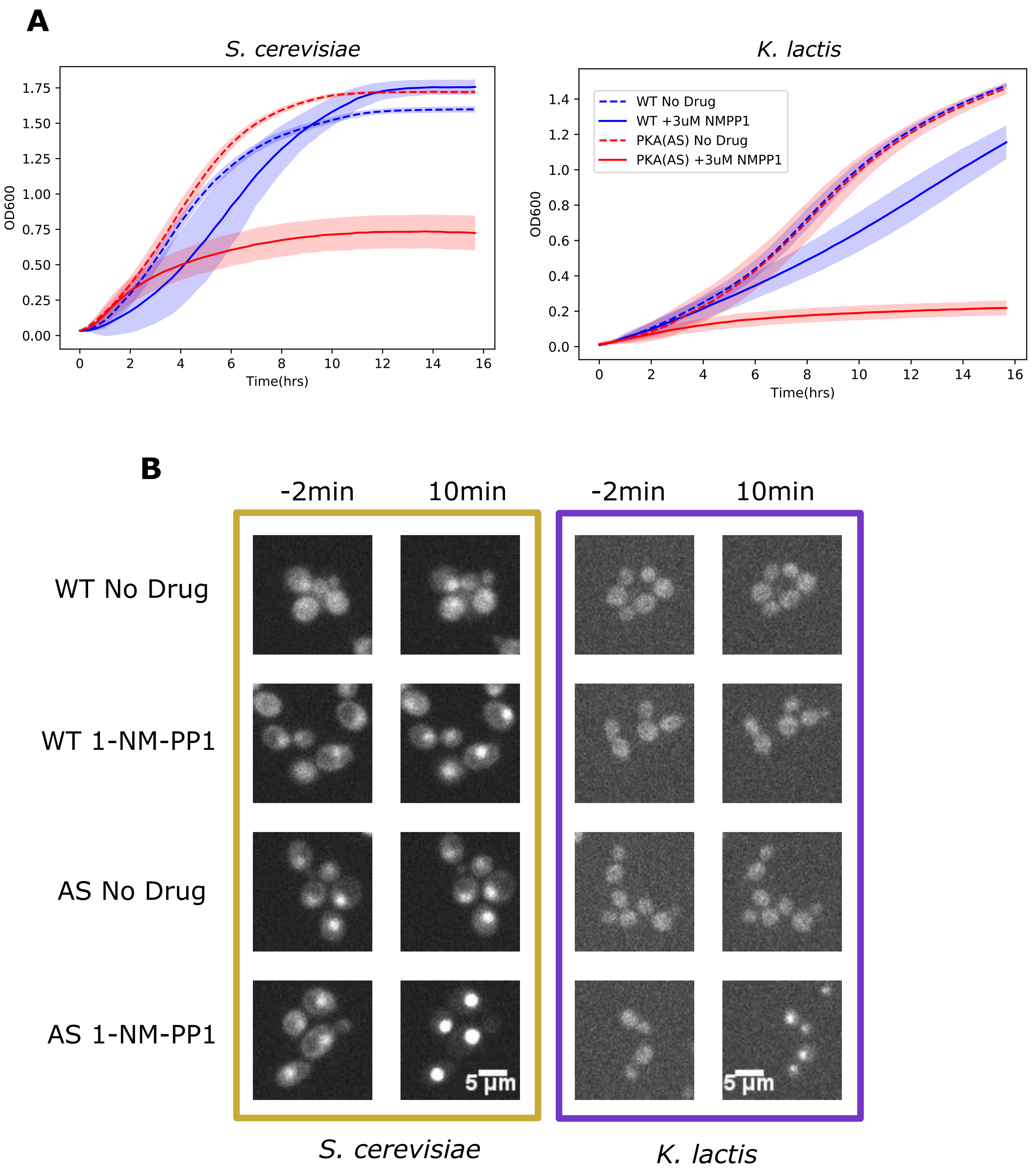
A
S. cerevisiae
K. lactis
OD600
Time(hrs)
WT No Drug
WT +3uM NMPP1
PKA(AS) No Drug
PKA(AS) +3uM NMPP1
B
-2min
10min
-2min
10min
WT No Drug
WT 1-NM-PP1
AS No Drug
AS 1-NM-PP1
5 µm
5 µm
S. cerevisiae
K. lactis

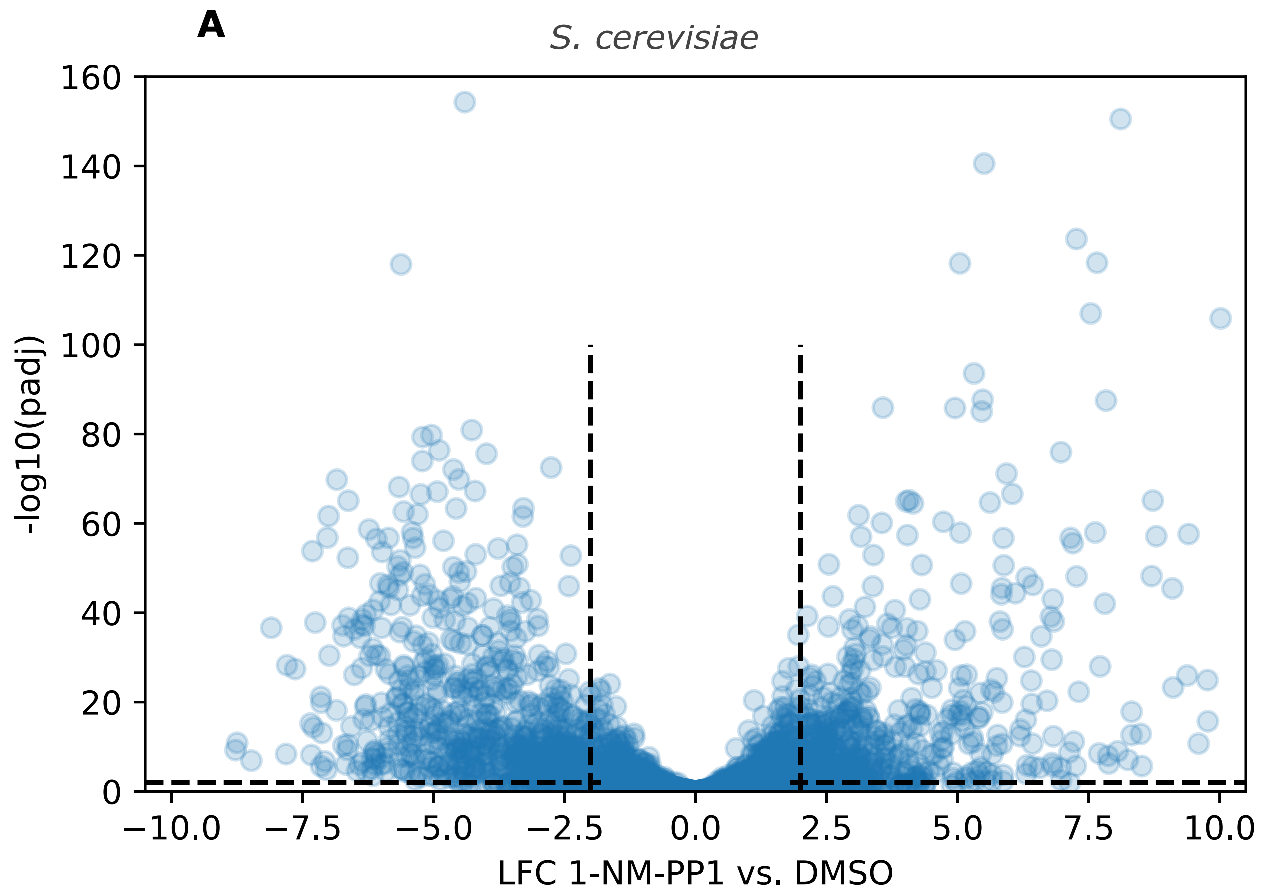
A
S. cerevisiae
160
140
120
100
80
60
40
20
0
-log10(padj)
−10.0
−7.5
−5.0
−2.5
0.0
2.5
5.0
7.5
10.0
LFC 1-NM-PP1 vs. DMSO

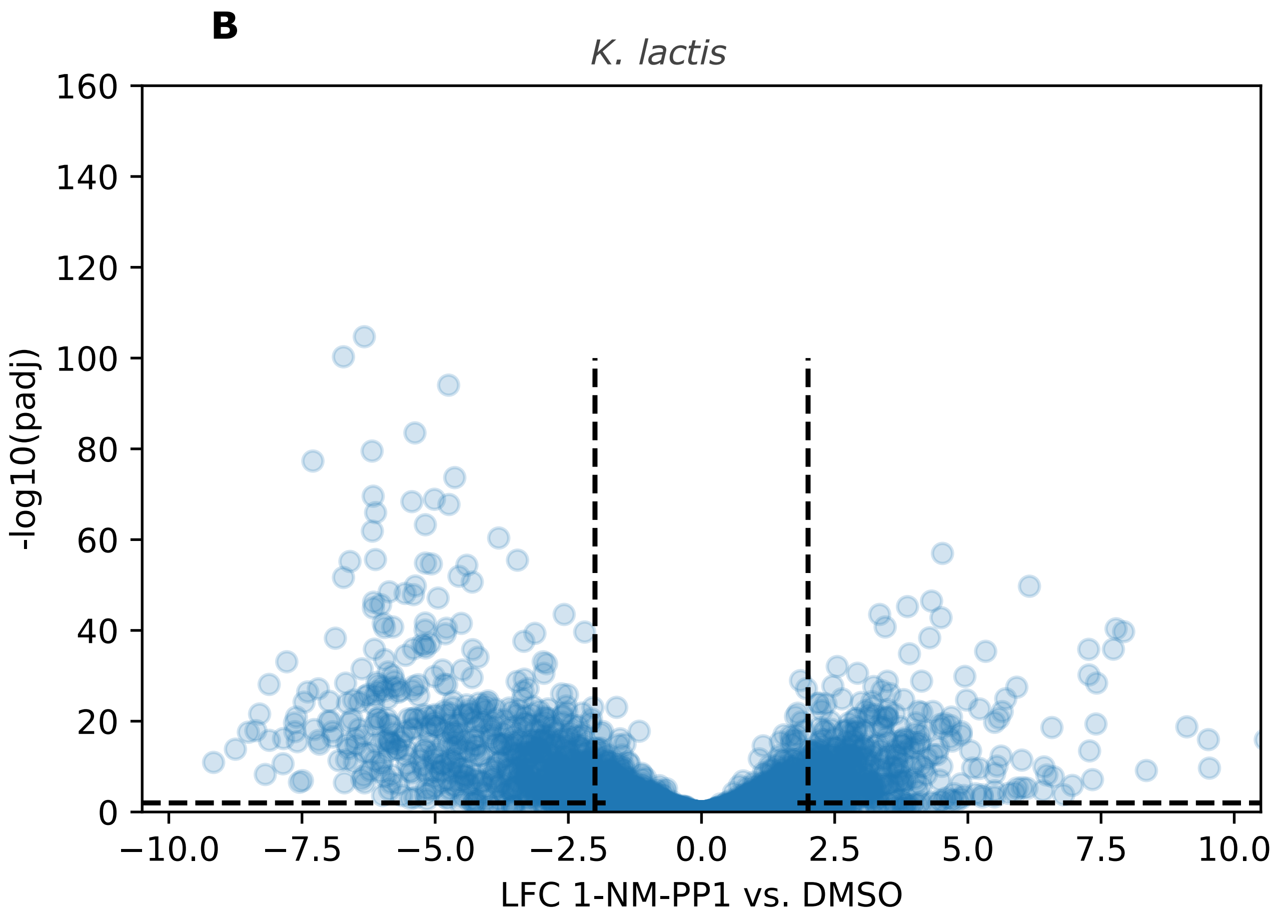
B
K. lactis
160
140
120
100
80
60
40
20
0
-log10(padj)
−10.0
−7.5
−5.0
−2.5
0.0
2.5
5.0
7.5
10.0
LFC 1-NM-PP1 vs. DMSO

S3

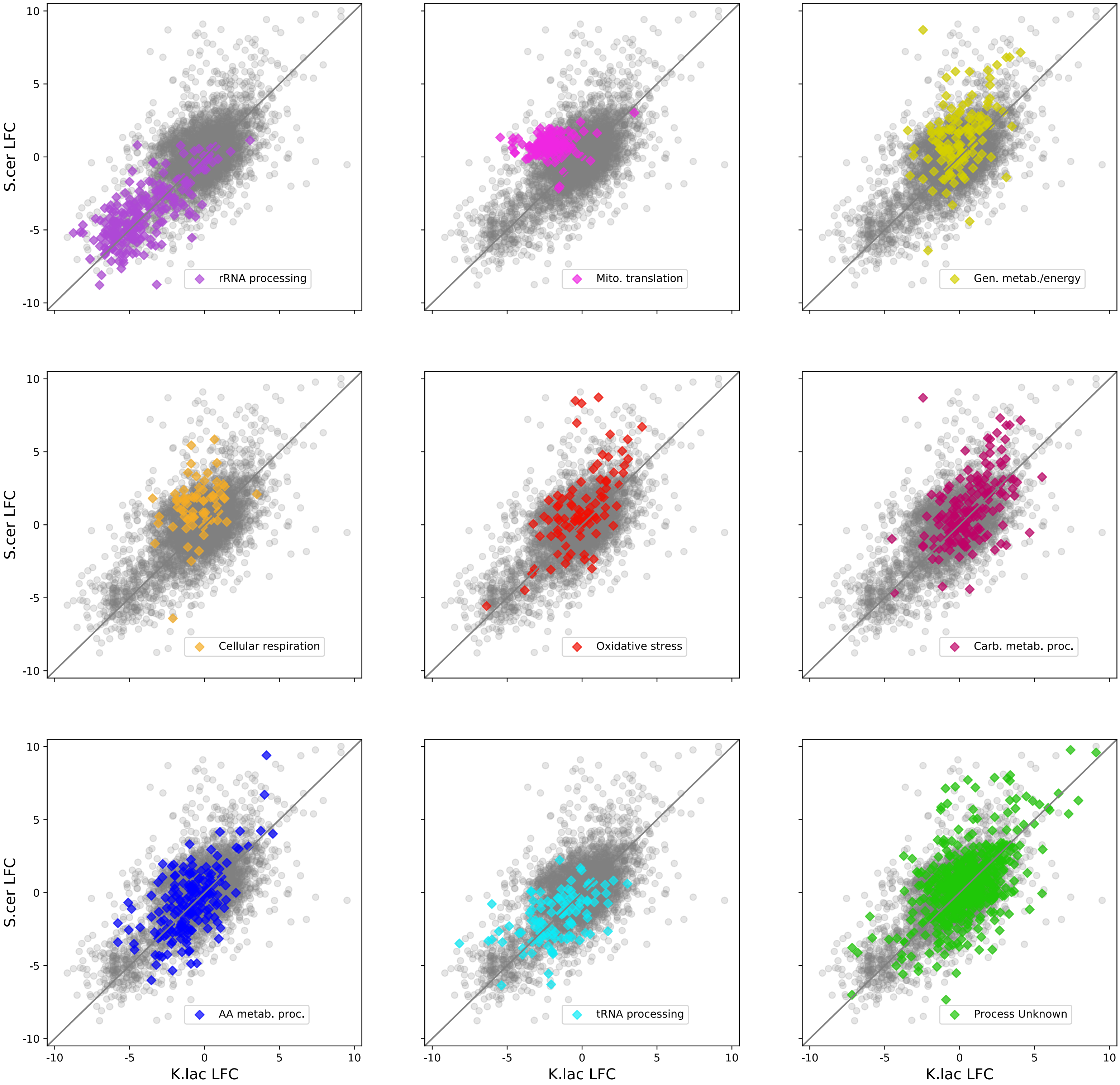

rRNA processing
Mito. translation
Gen. metab./energy
Cellular respiration
Oxidative stress
Carb. metab. proc.
AA metab. proc.
tRNA processing
Process Unknown
S.cer LFC
K.lac LFC
10
5
0
-5
-10

S4

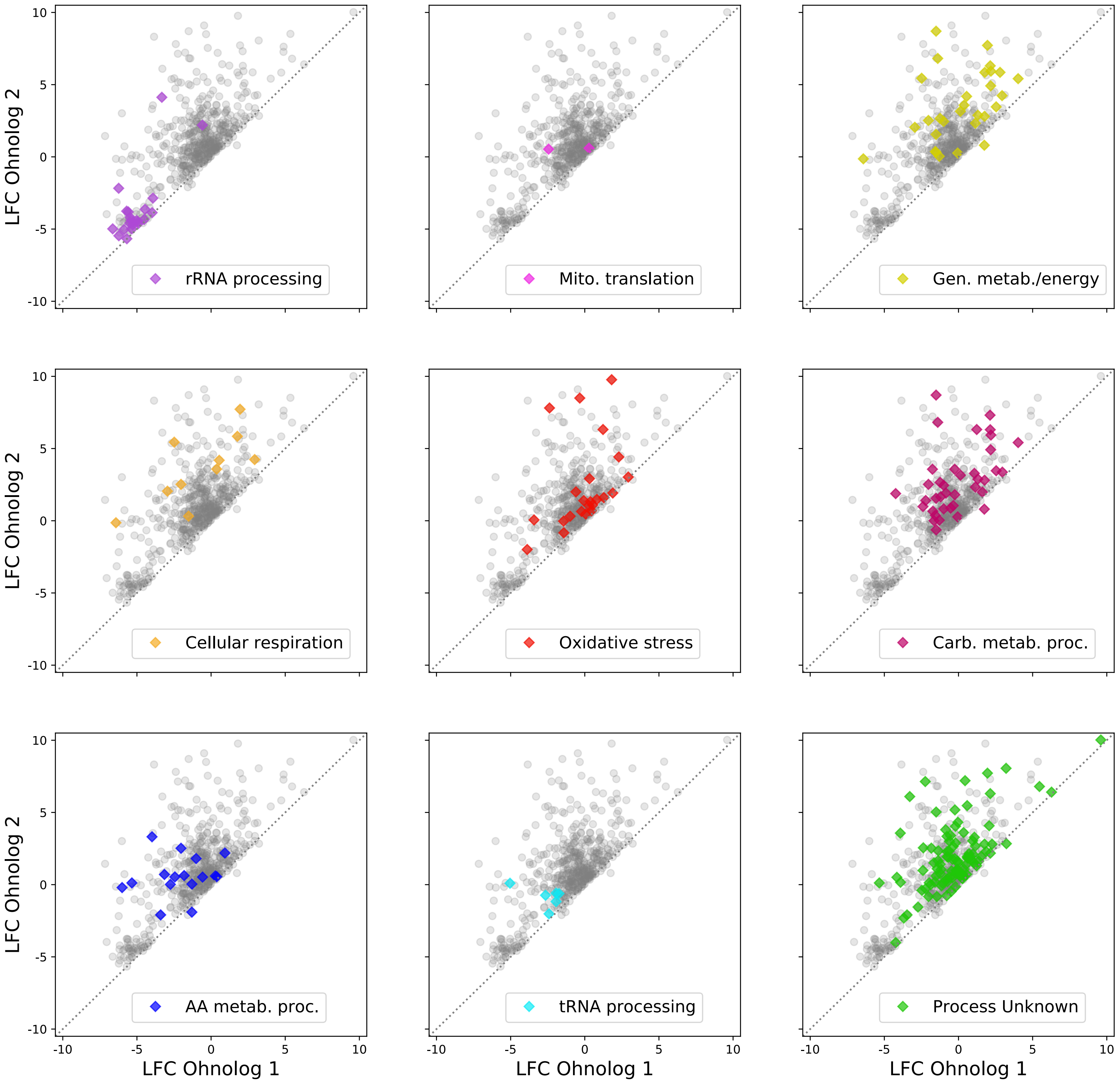

rRNA processing
Mito. translation
Gen. metab./energy
Cellular respiration
Oxidative stress
Carb. metab. proc.
AA metab. proc.
tRNA processing
Process Unknown
LFC Ohnolog 2
LFC Ohnolog 1

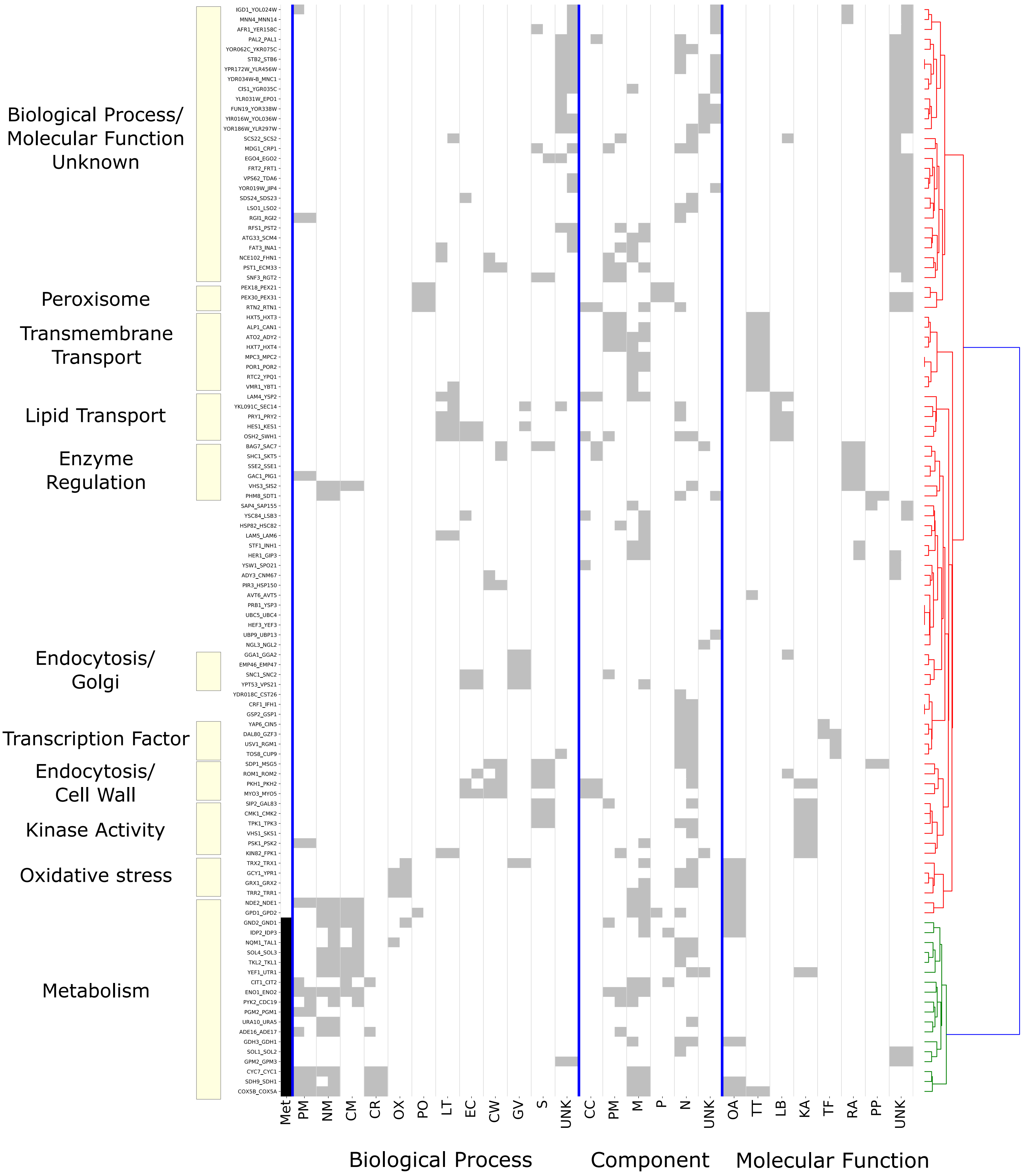

Biological Process/ Molecular Function Unknown
Peroxisome
Transmembrane Transport
Lipid Transport
Enzyme Regulation
Endocytosis/ Golgi
Transcription Factor
Endocytosis/ Cell Wall
Kinase Activity
Oxidative stress
Metabolism
IGD1_YOL024W
MNN4_MNN14
AFR1_YER158C
PAL2_PAL1
YOR062C_YKR075C
STB2_STB6
YPR172W_YLR456W
YDR034W-B_MNC1
CIS1_YGR035C
YLR031W_EPO1
FUN19_YOR338W
YIR016W_YOL036W
YOR186W_YLR297W
SCS22_SCS2
MDG1_CRP1
EGO4_EGO2
FRT2_FRT1
VPS62_TDA6
YOR019W_JIP4
SDS24_SDS23
LSO1_LSO2
RGI1_RGI2
RFS1_PST2
ATG33_SCM4
FAT3_INA1
NCE102_FHN1
PST1_ECM33
SNF3_RGT2
PEX18_PEX21
PEX30_PEX31
RTN2_RTN1
HXT5_HXT3
ALP1_CAN1
ATO2_ADY2
HXT7_HXT4
MPC3_MPC2
POR1_POR2
RTC2_YPQ1
VMR1_YBT1
LAM4_YSP2
YKL091C_SEC14
PRY1_PRY2
HES1_KES1
OSH2_SWH1
BAG7_SAC7
SHC1_SKT5
SSE2_SSE1
GAC1_PIG1
VHS3_SIS2
PHM8_SDT1
SAP4_SAP155
YSC84_LSB3
HSP82_HSC82
LAM5_LAM6
STF1_INH1
HER1_GIP3
YSW1_SPO21
ADY3_CNM67
PIR3_HSP150
AVT6_AVT5
PRB1_YSP3
UBC5_UBC4
HEF3_YEF3
UBP9_UBP13
NGL3_NGL2
GGA1_GGA2
EMP46_EMP47
SNC1_SNC2
YPT53_VPS21
YDR018C_CST26
CRF1_IFH1
GSP2_GSP1
YAP6_CIN5
DAL80_GZF3
USV1_RGM1
TOS8_CUP9
SDP1_MSG5
ROM1_ROM2
PKH1_PKH2
MYO3_MYO5
SIP2_GAL83
CMK1_CMK2
TPK1_TPK3
VHS1_SKS1
PSK1_PSK2
KIN82_FPK1
TRX2_TRX1
GCY1_YPR1
GRX1_GRX2
TRR2_TRR1
NDE2_NDE1
GPD1_GPD2
GND2_GND1
IDP2_IDP3
NQM1_TAL1
SOL4_SOL3
TKL2_TKL1
YEF1_UTR1
CIT1_CIT2
ENO1_ENO2
PYK2_CDC19
PGM2_PGM1
URA10_URA5
ADE16_ADE17
GDH3_GDH1
SOL1_SOL2
GPM2_GPM3
CYC7_CYC1
SDH9_SDH1
COX5B_COX5A
Met
PM
NM
CM
CR
OX
PO
LT
EC
CW
GV
S
UNK
CC
PM
M
P
N
UNK
OA
TT
LB
KA
TF
RA
PP
UNK
Biological Process
Component
Molecular Function

S6

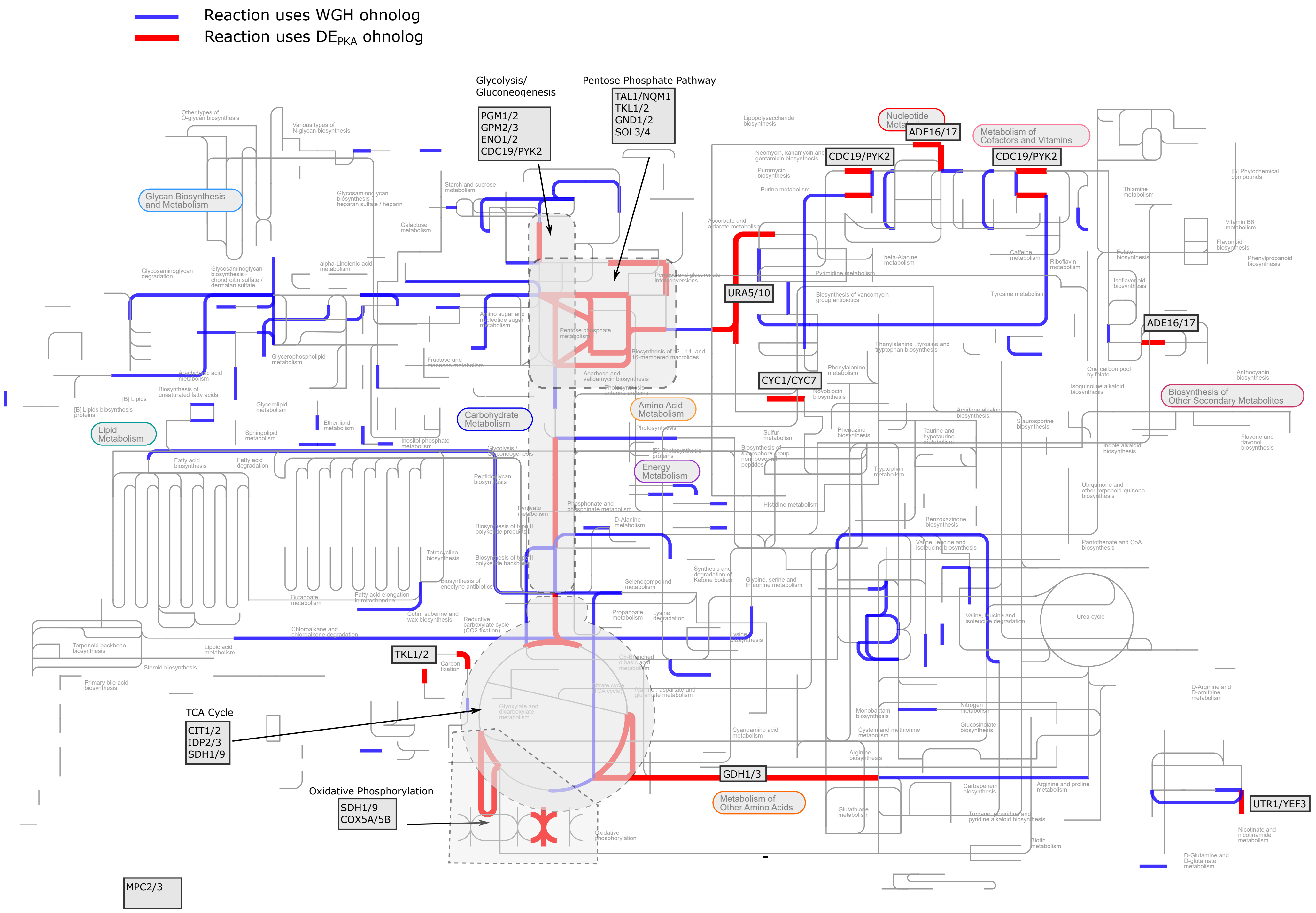
Reaction uses WGH ohnolog
Reaction uses DE$_{PKA}$ ohnolog
Glycolysis/
Gluconeogenesis
PGM1/2
GPM2/3
ENO1/2
CDC19/PYK2
Pentose Phosphate Pathway
TAL1/NQM1
TKL1/2
GND1/2
SOL3/4
Nucleotide
Metabolism
ADE16/17
Metabolism of
Cofactors and Vitamins
CDC19/PYK2
CDC19/PYK2
Glycan Biosynthesis
and Metabolism
URA5/10
ADE16/17
CYC1/CYC7
Amino Acid
Metabolism
Biosynthesis of
Other Secondary Metabolites
Carbohydrate
Metabolism
Lipid
Metabolism
Energy
Metabolism
TKL1/2
TCA Cycle
CIT1/2
IDP2/3
SDH1/9
Oxidative Phosphorylation
SDH1/9
COX5A/5B
GDH1/3
Metabolism of
Other Amino Acids
UTR1/YEF3
MPC2/3

S7

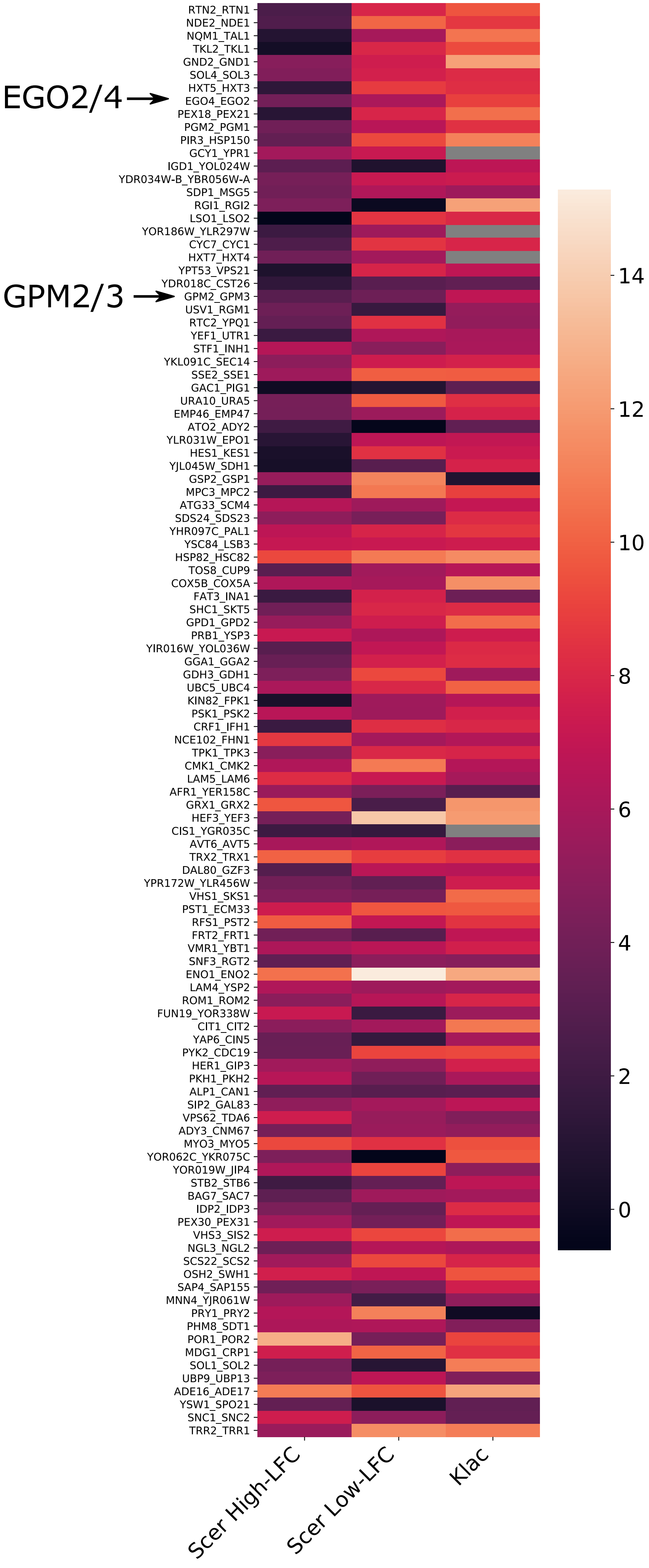
EGO2/4
GPM2/3
RTN2_RTN1
NDE2_NDE1
NQM1_TAL1
TKL2_TKL1
GND2_GND1
SOL4_SOL3
HXT5_HXT3
EGO4_EGO2
PEX18_PEX21
PGM2_PGM1
PIR3_HSP150
GCY1_YPR1
IGD1_YOL024W
YDR034W-B_YBR056W-A
SDP1_MSG5
RGI1_RGI2
LSO1_LSO2
YOR186W_YLR297W
CYC7_CYC1
HXT7_HXT4
YPT53_VPS21
YDR018C_CST26
GPM2_GPM3
USV1_RGM1
RTC2_YPQ1
YEF1_UTR1
STF1_INH1
YKL091C_SEC14
SSE2_SSE1
GAC1_PIG1
URA10_URA5
EMP46_EMP47
ATO2_ADY2
YLR031W_EPO1
HES1_KES1
YJL045W_SDH1
GSP2_GSP1
MPC3_MPC2
ATG33_SCM4
SDS24_SDS23
YHR097C_PAL1
YSC84_LSB3
HSP82_HSC82
TOS8_CUP9
COX5B_COX5A
FAT3_INA1
SHC1_SKT5
GPD1_GPD2
PRB1_YSP3
YIR016W_YOL036W
GGA1_GGA2
GDH3_GDH1
UBC5_UBC4
KIN82_FPK1
PSK1_PSK2
CRF1_IFH1
NCE102_FHN1
TPK1_TPK3
CMK1_CMK2
LAM5_LAM6
AFR1_YER158C
GRX1_GRX2
HEF3_YEF3
CIS1_YGR035C
AVT6_AVT5
TRX2_TRX1
DAL80_GZF3
YPR172W_YLR456W
VHS1_SKS1
PST1_ECM33
RFS1_PST2
FRT2_FRT1
VMR1_YBT1
SNF3_RGT2
ENO1_ENO2
LAM4_YSP2
ROM1_ROM2
FUN19_YOR338W
CIT1_CIT2
YAP6_CIN5
PYK2_CDC19
HER1_GIP3
PKH1_PKH2
ALP1_CAN1
SIP2_GAL83
VPS62_TDA6
ADY3_CNM67
MYO3_MYO5
YOR062C_YKR075C
YOR019W_JIP4
STB2_STB6
BAG7_SAC7
IDP2_IDP3
PEX30_PEX31
VHS3_SIS2
NGL3_NGL2
SCS22_SCS2
OSH2_SWH1
SAP4_SAP155
MNN4_YJR061W
PRY1_PRY2
PHM8_SDT1
POR1_POR2
MDG1_CRP1
SOL1_SOL2
UBP9_UBP13
ADE16_ADE17
YSW1_SPO21
SNC1_SNC2
TRR2_TRR1
Scer High-LFC
Scer Low-LFC
Klac
14
12
10
8
6
4
2
0

LFC correlation between PKA inhhibition and stress conditions.
Stress data from Roy et al. 2013 and Thompson et al. 2013.

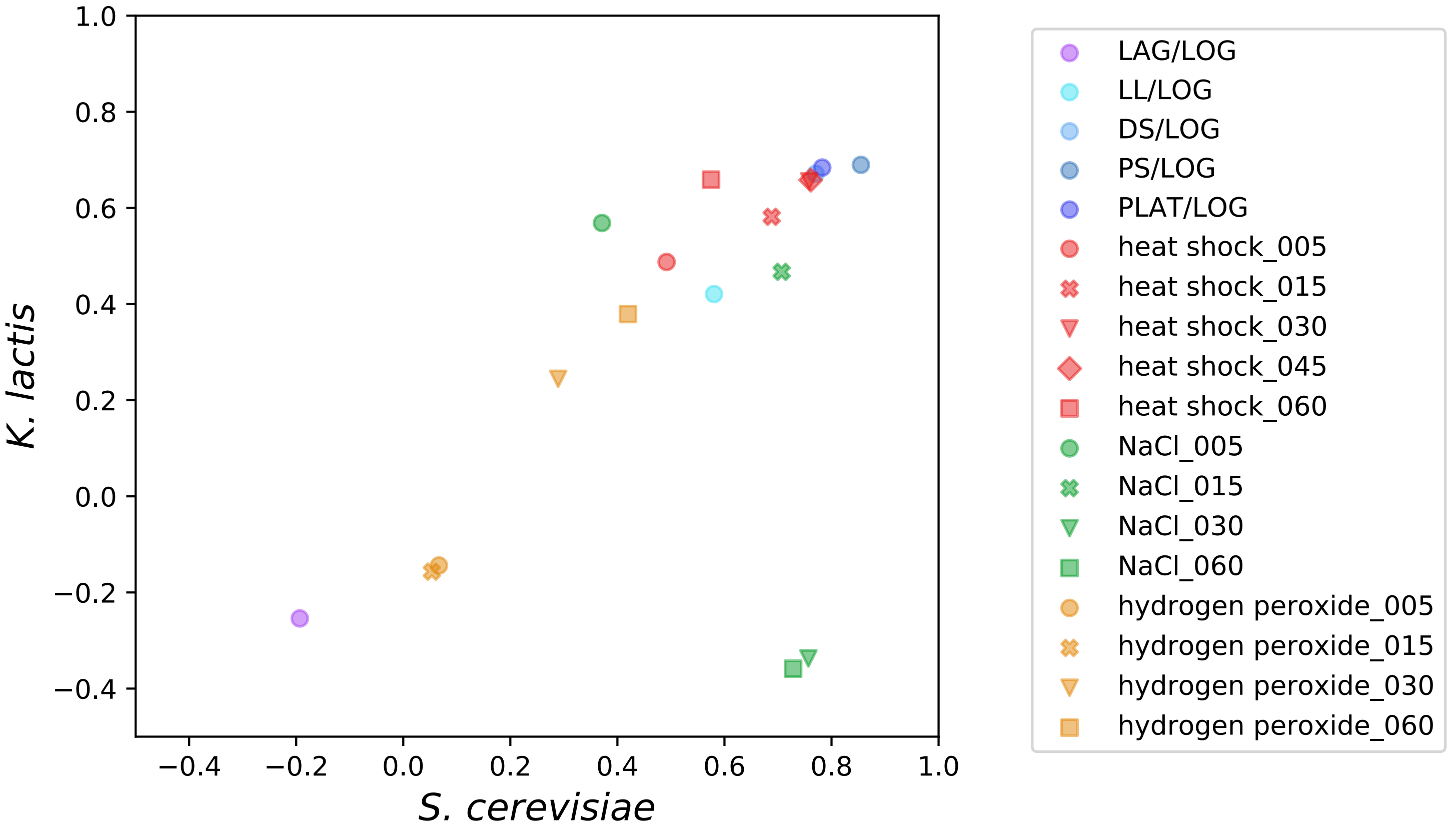

S9

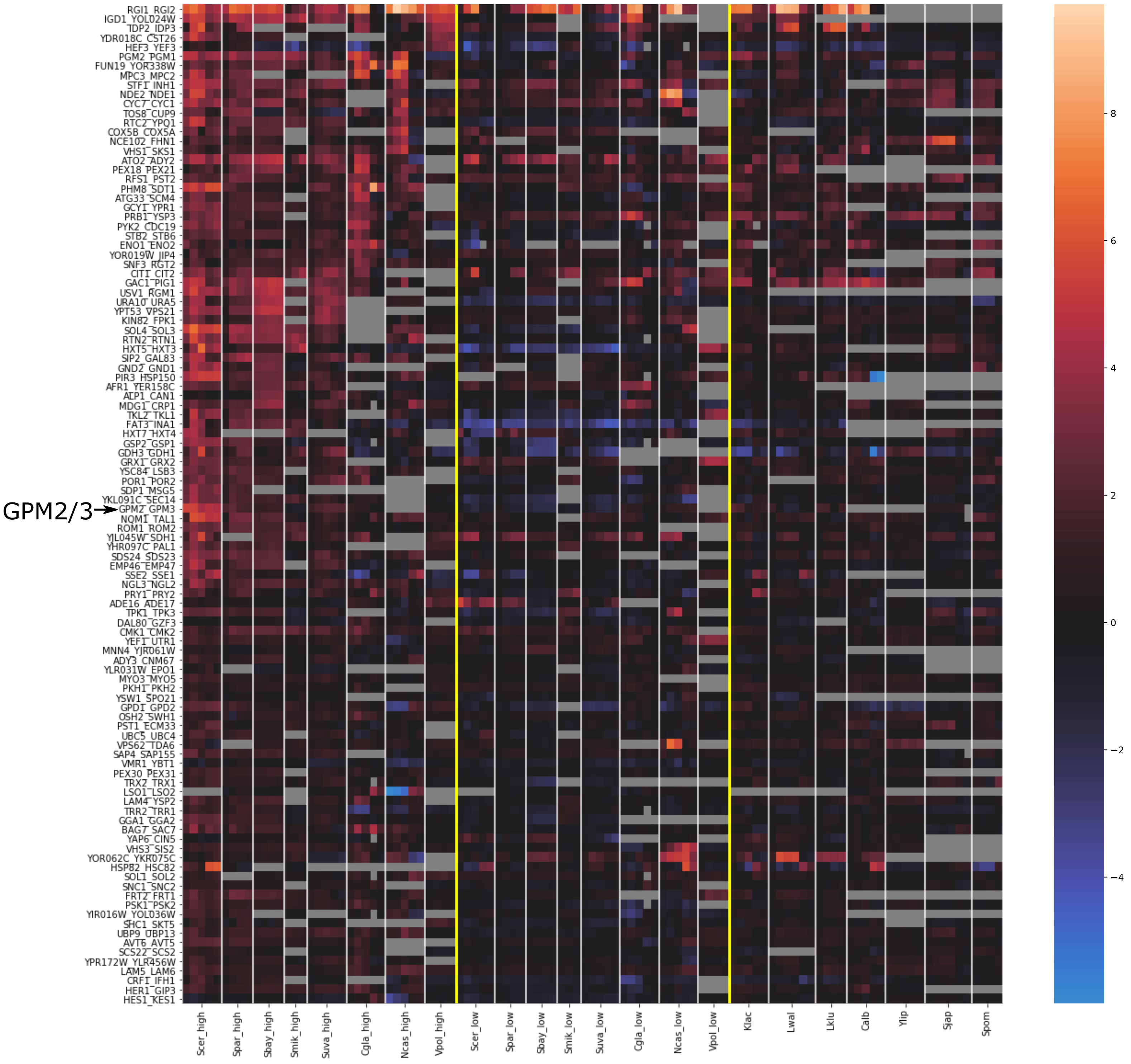
RGI1_RGI2
IGD1_YOL024W
IDP2_IDP3
YDR018C_CST26
HEF3_YEF3
PGM2_PGM1
FUN19_YOR338W
MPC3_MPC2
STF1_INH1
NDE2_NDE1
CYC7_CYC1
TOS8_CUP9
RTC2_YPQ1
COX5B_COX5A
NCE102_FHN1
VHS1_SKS1
ATO2_ADY2
PEX18_PEX21
RFS1_PST2
PHM8_SDT1
ATG33_SCM4
GCY1_YPR1
PRB1_YSP3
PYK2_CDC19
STB2_STB6
ENO1_ENO2
YOR019W_JIP4
SNF3_RGT2
CIT1_CIT2
GAC1_PIG1
USV1_RGM1
URA10_URA5
YPT53_VPS21
KIN82_FPK1
SOL4_SOL3
RTN2_RTN1
HXT5_HXT3
SIP2_GAL83
GND2_GND1
PIR3_HSP150
AFR1_YER158C
ALP1_CAN1
MDG1_CRP1
TKL2_TKL1
FAT3_INA1
HXT7_HXT4
GSP2_GSP1
GDH3_GDH1
GRX1_GRX2
YSC84_LSB3
POR1_POR2
SDP1_MSG5
YKL091C_SEC14
GPM2/3
GPM2_GPM3
NQM1_TAL1
ROM1_ROM2
YJL045W_SDH1
YHR097C_PAL1
SDS24_SDS23
EMP46_EMP47
SSE2_SSE1
NGL3_NGL2
PRY1_PRY2
ADE16_ADE17
TPK1_TPK3
DAL80_GZF3
CMK1_CMK2
YEF1_UTR1
MNN4_YJR061W
ADY3_CNM67
YLR031W_EPO1
MYO3_MYO5
PKH1_PKH2
YSW1_SPO21
GPD1_GPD2
OSH2_SWH1
PST1_ECM33
UBC5_UBC4
VPS62_TDA6
SAP4_SAP155
VMR1_YBT1
PEX30_PEX31
TRX2_TRX1
LSO1_LSO2
LAM4_YSP2
TRR2_TRR1
GGA1_GGA2
BAG7_SAC7
YAP6_CIN5
VHS3_SIS2
YOR062C_YKR075C
HSP82_HSC82
SOL1_SOL2
SNC1_SNC2
FRT2_FRT1
PSK1_PSK2
YIR016W_YOL036W
SHC1_SKT5
UBP9_UBP13
AVT6_AVT5
SCS22_SCS2
YPR172W_YLR456W
LAM5_LAM6
CRF1_IFH1
HER1_GIP3
HES1_KES1
Scer_high
Spar_high
Sbay_high
Smik_high
Suva_high
Cgla_high
Ncas_high
Vpol_high
Scer_low
Spar_low
Sbay_low
Smik_low
Suva_low
Cgla_low
Ncas_low
Vpol_low
Klac
Lwal
Lklu
Calb
Ylip
Sjap
Spom
8
6
4
2
0
−2
−4

A

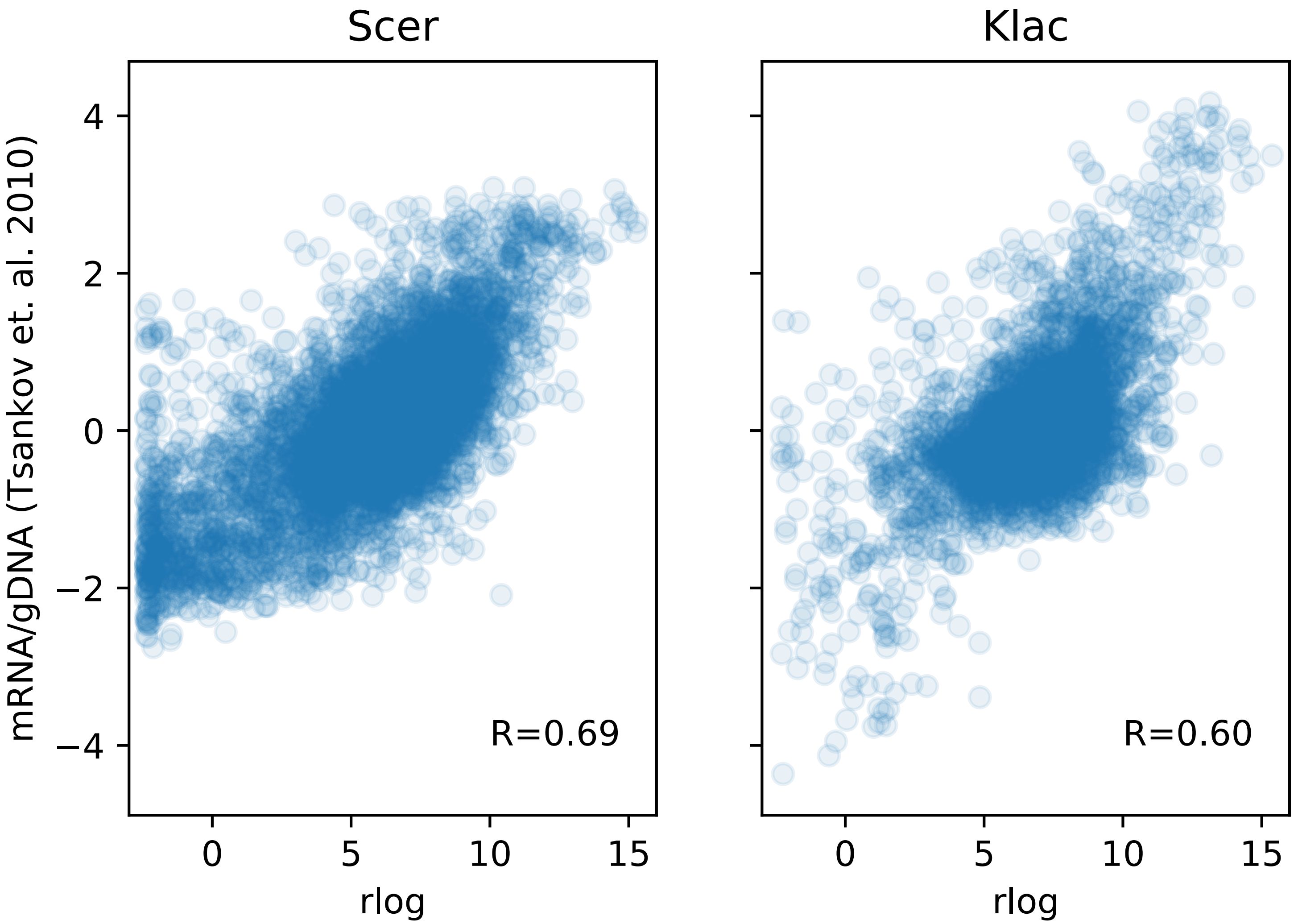
Scer
Klac
mRNA/gDNA (Tsankov et. al. 2010)
R=0.69
R=0.60
rlog
rlog
4
2
0
−2
−4
0
5
10
15

B

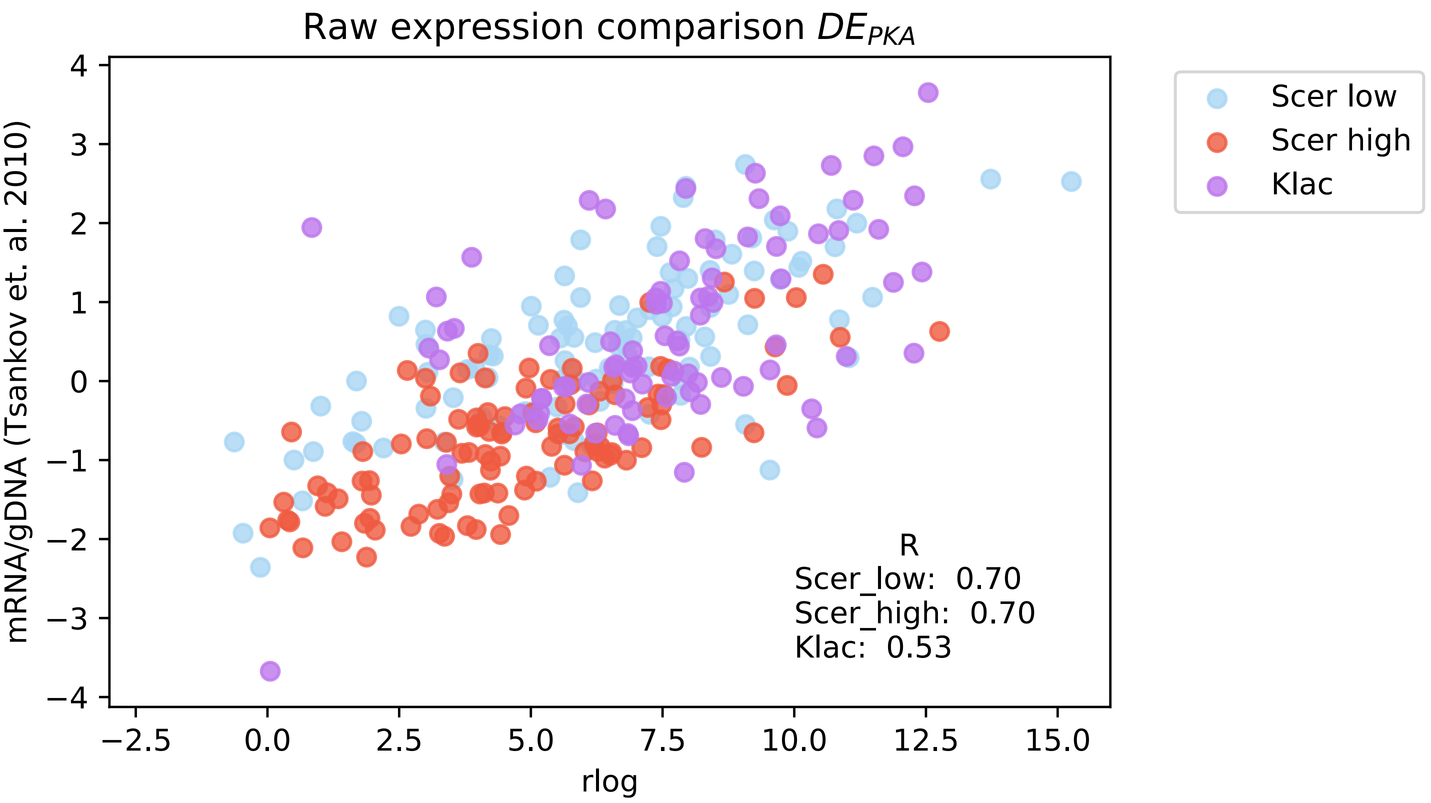
Raw expression comparison DE_{PKA}
mRNA/gDNA (Tsankov et. al. 2010)
rlog
Scer low
Scer high
Klac
R
Scer_low: 0.70
Scer_high: 0.70
Klac: 0.53
−2.5
0.0
2.5
5.0
7.5
10.0
12.5
15.0

S11

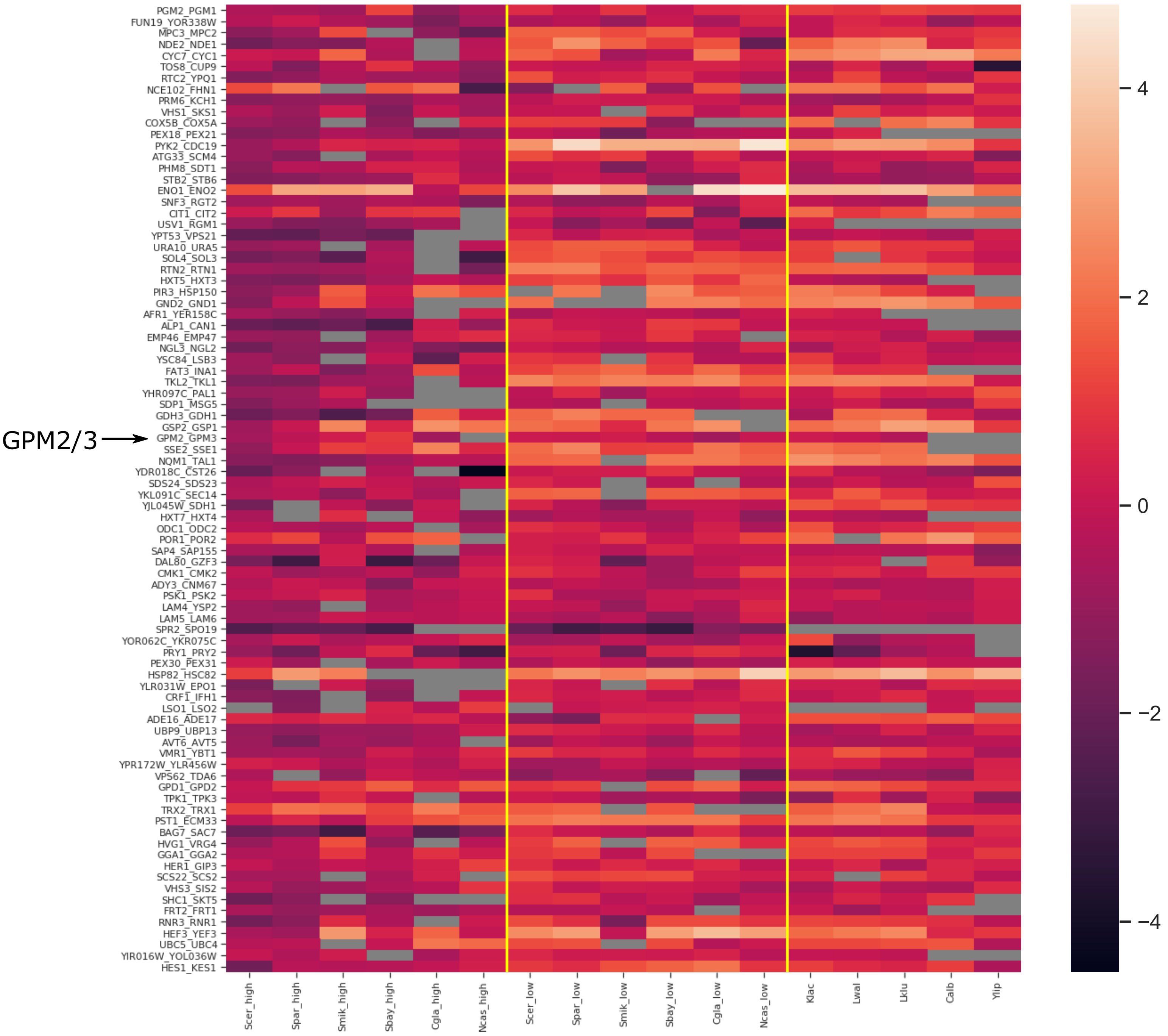

GPM2/3
PGM2_PGM1
FUN19_YOR338W
MPC3_MPC2
NDE2_NDE1
CYC7_CYC1
TOS8_CUP9
RTC2_YPQ1
NCE102_FHN1
PRM6_KCH1
VHS1_SKS1
COX5B_COX5A
PEX18_PEX21
PYK2_CDC19
ATG33_SCM4
PHM8_SDT1
STB2_STB6
ENO1_ENO2
SNF3_RGT2
CIT1_CIT2
USV1_RGM1
YPT53_VPS21
URA10_URA5
SOL4_SOL3
RTN2_RTN1
HXT5_HXT3
PIR3_HSP150
GND2_GND1
AFR1_YER158C
ALP1_CAN1
EMP46_EMP47
NGL3_NGL2
YSC84_LSB3
FAT3_INA1
TKL2_TKL1
YHR097C_PAL1
SDP1_MSG5
GDH3_GDH1
GSP2_GSP1
GPM2_GPM3
SSE2_SSE1
NQM1_TAL1
YDR018C_CST26
SDS24_SDS23
YKL091C_SEC14
YJL045W_SDH1
HXT7_HXT4
ODC1_ODC2
POR1_POR2
SAP4_SAP155
DAL80_GZF3
CMK1_CMK2
ADY3_CNM67
PSK1_PSK2
LAM4_YSP2
LAM5_LAM6
SPR2_SPO19
YOR062C_YKR075C
PRY1_PRY2
PEX30_PEX31
HSP82_HSC82
YLR031W_EPO1
CRF1_IFH1
LSO1_LSO2
ADE16_ADE17
UBP9_UBP13
AVT6_AVT5
VMR1_YBT1
YPR172W_YLR456W
VPS62_TDA6
GPD1_GPD2
TPK1_TPK3
TRX2_TRX1
PST1_ECM33
BAG7_SAC7
HVG1_VRG4
GGA1_GGA2
HER1_GIP3
SCS22_SCS2
VHS3_SIS2
SHC1_SKT5
FRT2_FRT1
RNR3_RNR1
HEF3_YEF3
UBC5_UBC4
YIR016W_YOL036W
HES1_KES1
Scer_high
Spar_high
Smik_high
Sbay_high
Cgla_high
Ncas_high
Scer_low
Spar_low
Smik_low
Sbay_low
Cgla_low
Ncas_low
Klac
Lwal
Lklu
Calb
Ylip
4
2
0
−2
−4

S12

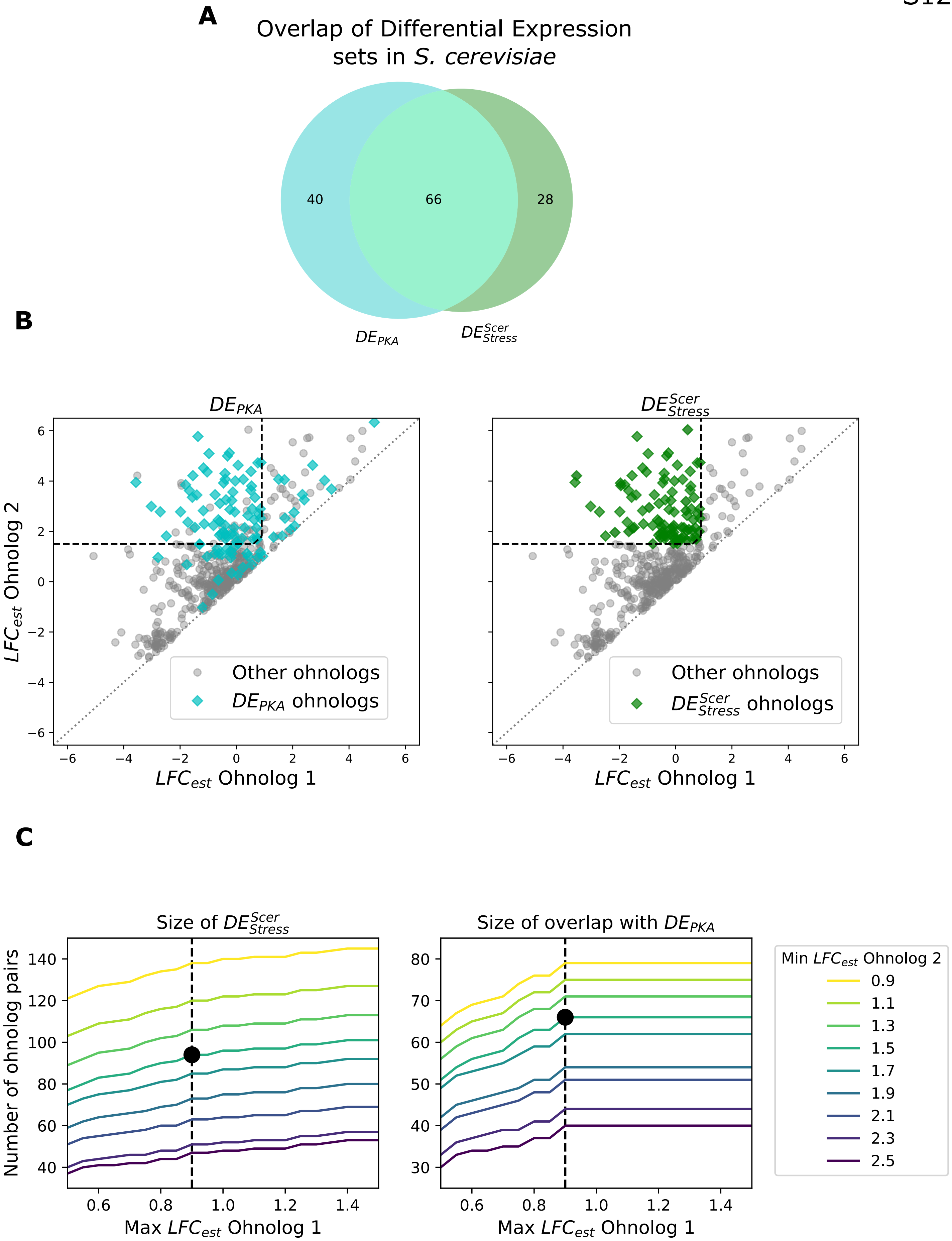
A
Overlap of Differential Expression sets in S. cerevisiae
40
66
28
DE_{PKA}
DE^{Scer}_{Stress}
B
DE_{PKA}
DE^{Scer}_{Stress}
LFC_{est} Ohnolog 2
LFC_{est} Ohnolog 1
Other ohnologs
DE_{PKA} ohnologs
DE^{Scer}_{Stress} ohnologs
C
Size of DE^{Scer}_{Stress}
Size of overlap with DE_{PKA}
Number of ohnolog pairs
Max LFC_{est} Ohnolog 1
Min LFC_{est} Ohnolog 2
0.9
1.1
1.3
1.5
1.7
1.9
2.1
2.3
2.5

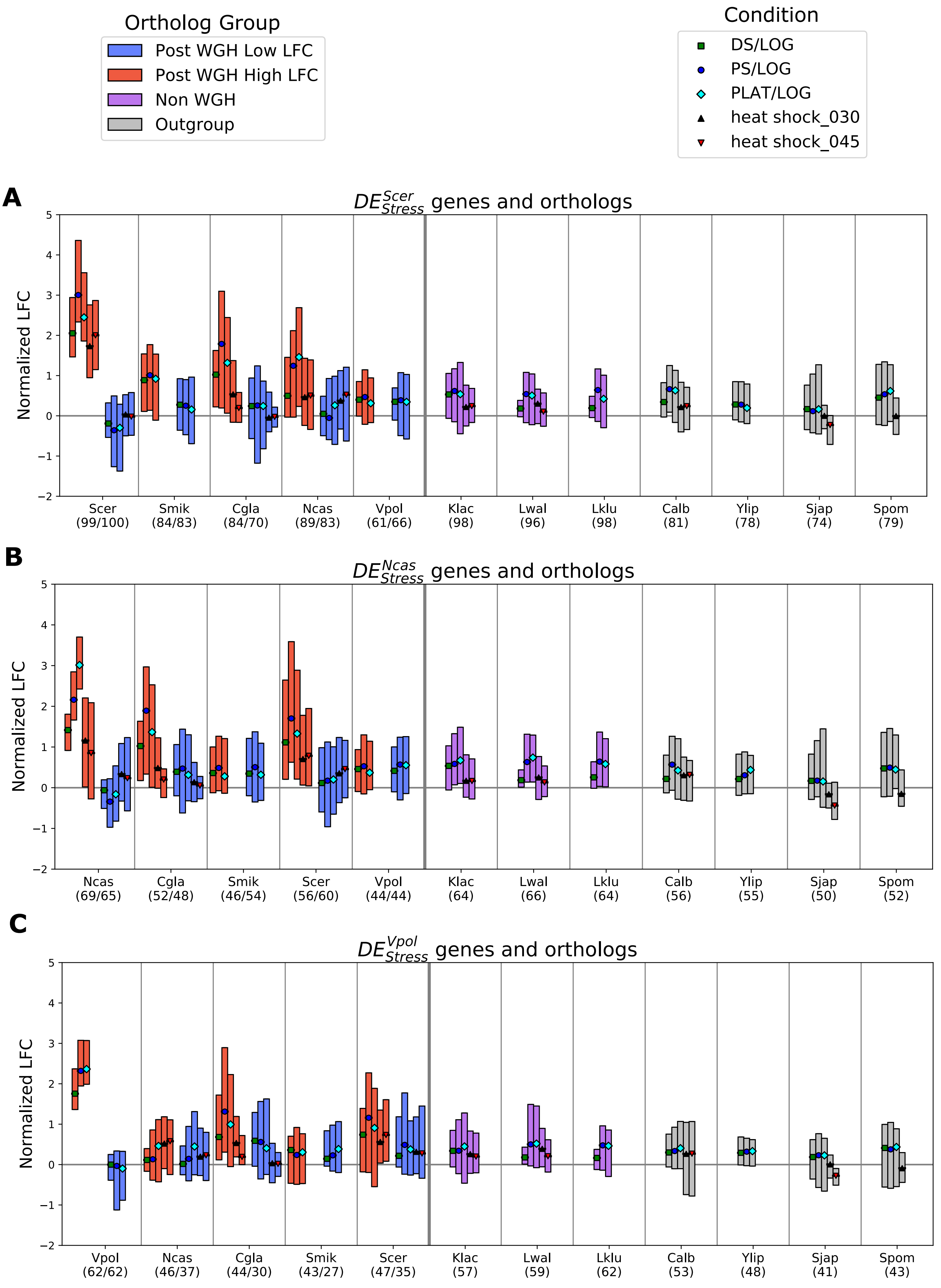

Data from Thompson et al. 2013 and Roy et al. 2013.

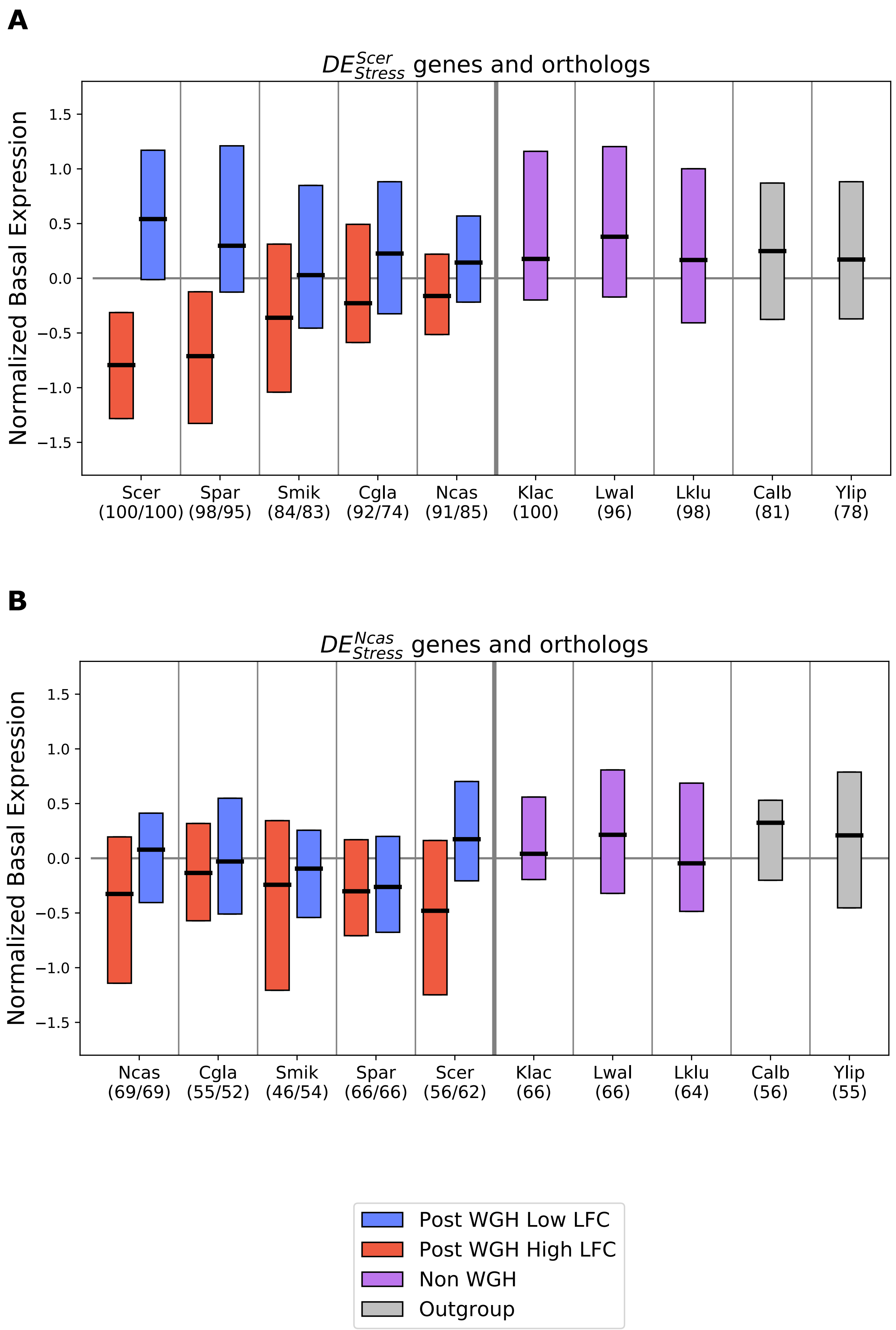
A
DE^{Scer}_{Stress} genes and orthologs
Normalized Basal Expression
1.5
1.0
0.5
0.0
−0.5
−1.0
−1.5
Scer (100/100)
Spar (98/95)
Smik (84/83)
Cgla (92/74)
Ncas (91/85)
Klac (100)
Lwal (96)
Lklu (98)
Calb (81)
Ylip (78)
B
DE^{Ncas}_{Stress} genes and orthologs
Normalized Basal Expression
1.5
1.0
0.5
0.0
−0.5
−1.0
−1.5
Ncas (69/69)
Cgla (55/52)
Smik (46/54)
Spar (66/66)
Scer (56/62)
Klac (66)
Lwal (66)
Lklu (64)
Calb (56)
Ylip (55)
Post WGH Low LFC
Post WGH High LFC
Non WGH
Outgroup

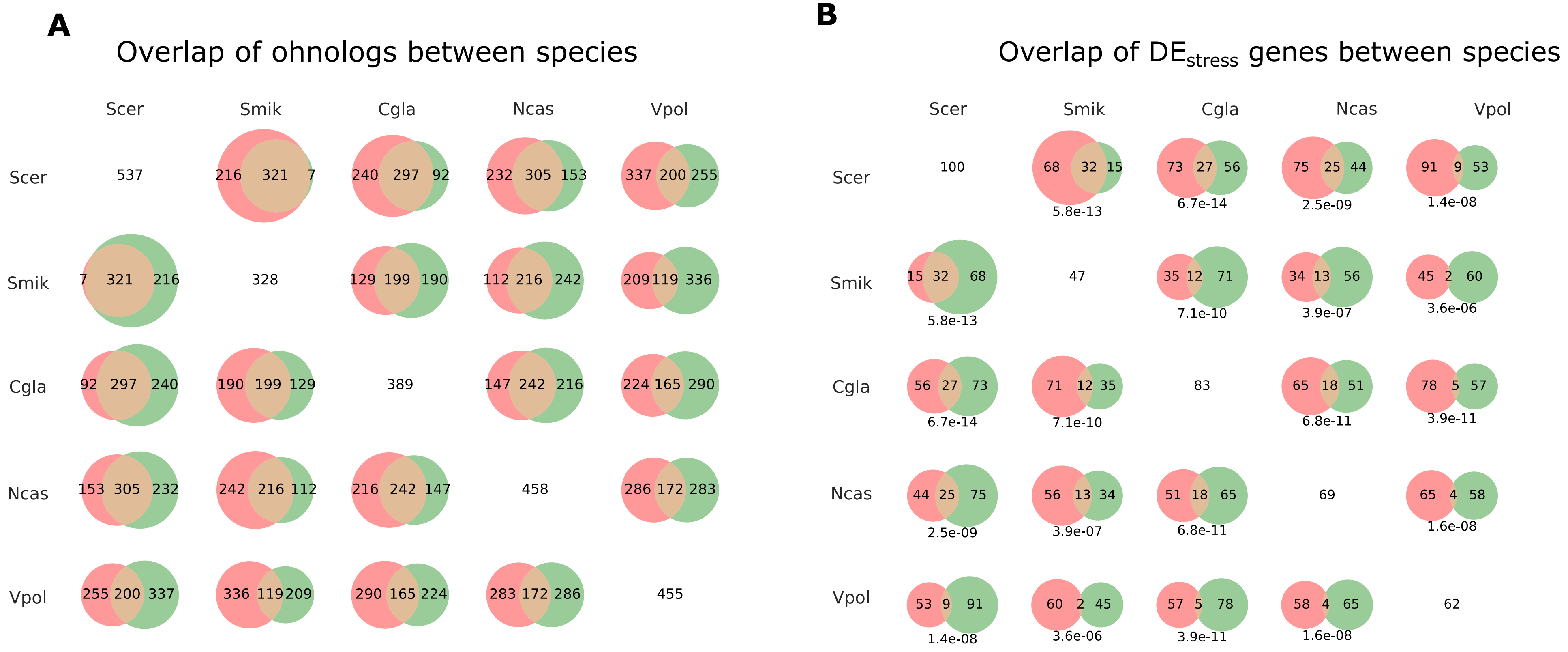
A
Overlap of ohnologs between species
Scer Smik Cgla Ncas Vpol
Scer: 537 | 216 321 7 | 240 297 92 | 232 305 153 | 337 200 255
Smik: 7 321 216 | 328 | 129 199 190 | 112 216 242 | 209 119 336
Cgla: 92 297 240 | 190 199 129 | 389 | 147 242 216 | 224 165 290
Ncas: 153 305 232 | 242 216 112 | 216 242 147 | 458 | 286 172 283
Vpol: 255 200 337 | 336 119 209 | 290 165 224 | 283 172 286 | 455
B
Overlap of DE$_{stress}$ genes between species
Scer Smik Cgla Ncas Vpol
Scer: 100 | 68 32 15 5.8e-13 | 73 27 56 6.7e-14 | 75 25 44 2.5e-09 | 91 9 53 1.4e-08
Smik: 15 32 68 5.8e-13 | 47 | 35 12 71 7.1e-10 | 34 13 56 3.9e-07 | 45 2 60 3.6e-06
Cgla: 56 27 73 6.7e-14 | 71 12 35 7.1e-10 | 83 | 65 18 51 6.8e-11 | 78 5 57 3.9e-11
Ncas: 44 25 75 2.5e-09 | 56 13 34 3.9e-07 | 51 18 65 6.8e-11 | 69 | 65 4 58 1.6e-08
Vpol: 53 9 91 1.4e-08 | 60 2 45 3.6e-06 | 57 5 78 3.9e-11 | 58 4 65 1.6e-08 | 62

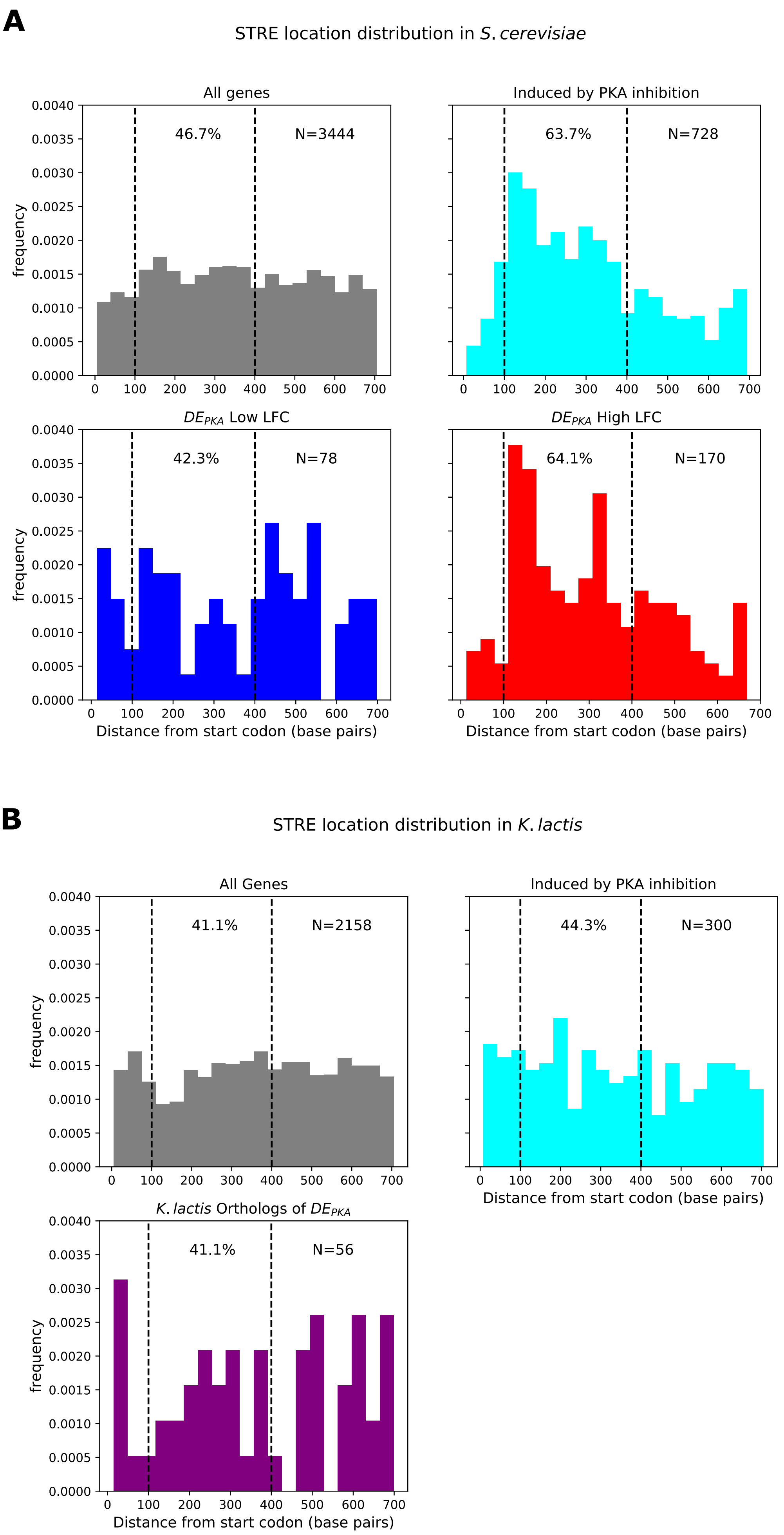
A
STRE location distribution in S. cerevisiae
All genes
46.7%
N=3444
Induced by PKA inhibition
63.7%
N=728
DE_{PKA} Low LFC
42.3%
N=78
DE_{PKA} High LFC
64.1%
N=170
frequency
Distance from start codon (base pairs)
0.0000
0.0005
0.0010
0.0015
0.0020
0.0025
0.0030
0.0035
0.0040
0 100 200 300 400 500 600 700
B
STRE location distribution in K. lactis
All Genes
41.1%
N=2158
Induced by PKA inhibition
44.3%
N=300
K. lactis Orthologs of DE_{PKA}
41.1%
N=56
frequency
Distance from start codon (base pairs)

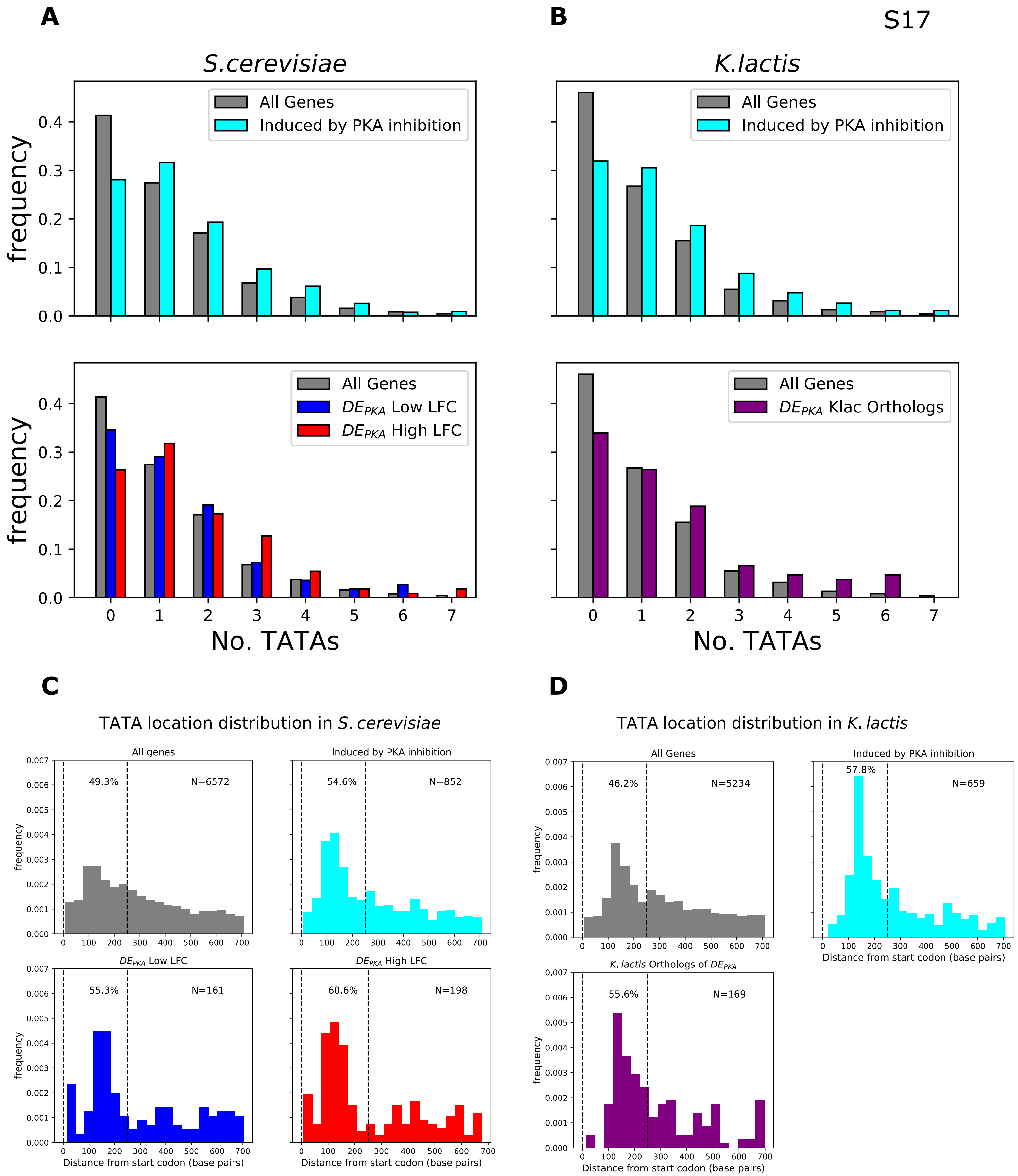
A
B
S.cerevisiae
K.lactis
All Genes
Induced by PKA inhibition
frequency
All Genes
DE_PKA Low LFC
DE_PKA High LFC
All Genes
DE_PKA Klac Orthologs
No. TATAs
C
D
TATA location distribution in S. cerevisiae
TATA location distribution in K. lactis
All genes
49.3%
N=6572
Induced by PKA inhibition
54.6%
N=852
DE_PKA Low LFC
55.3%
N=161
DE_PKA High LFC
60.6%
N=198
All Genes
46.2%
N=5234
Induced by PKA inhibition
57.8%
N=659
K. lactis Orthologs of DE_PKA
55.6%
N=169
frequency
Distance from start codon (base pairs)

A

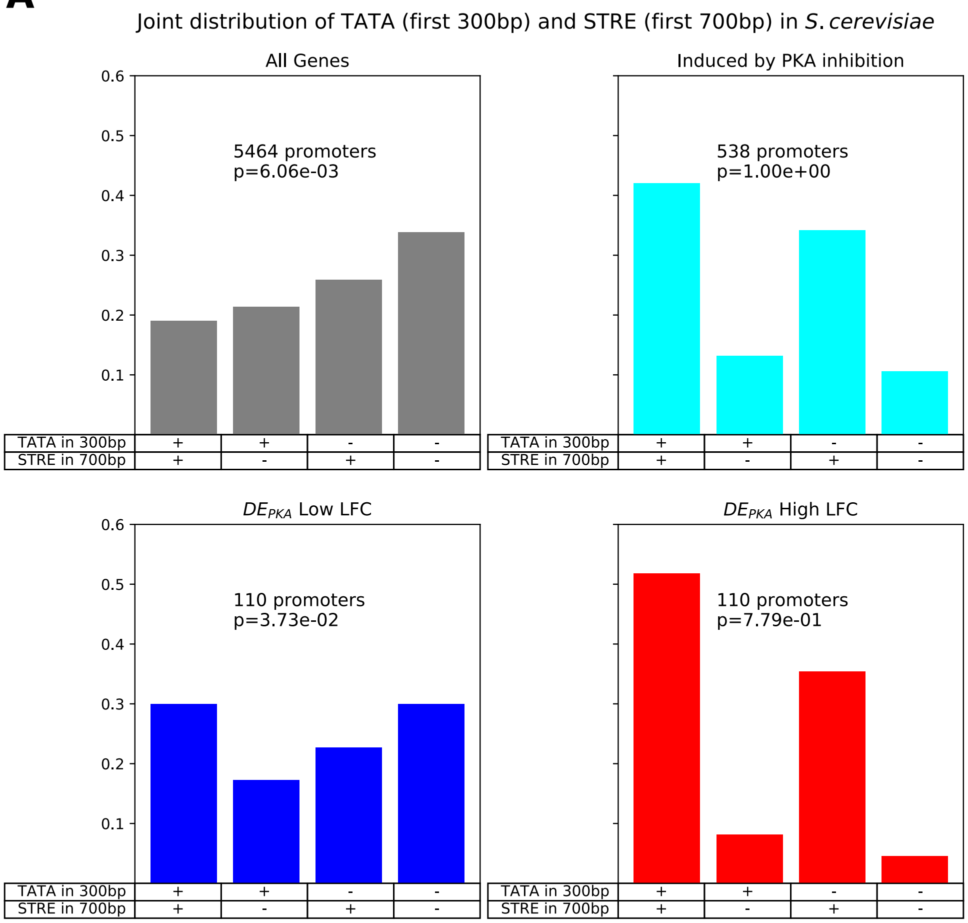
Joint distribution of TATA (first 300bp) and STRE (first 700bp) in *S. cerevisiae*
All Genes
5464 promoters
p=6.06e-03
Induced by PKA inhibition
538 promoters
p=1.00e+00
DE_{PKA} Low LFC
110 promoters
p=3.73e-02
DE_{PKA} High LFC
110 promoters
p=7.79e-01
0.6
0.5
0.4
0.3
0.2
0.1
TATA in 300bp + + - -
STRE in 700bp + - + -

B

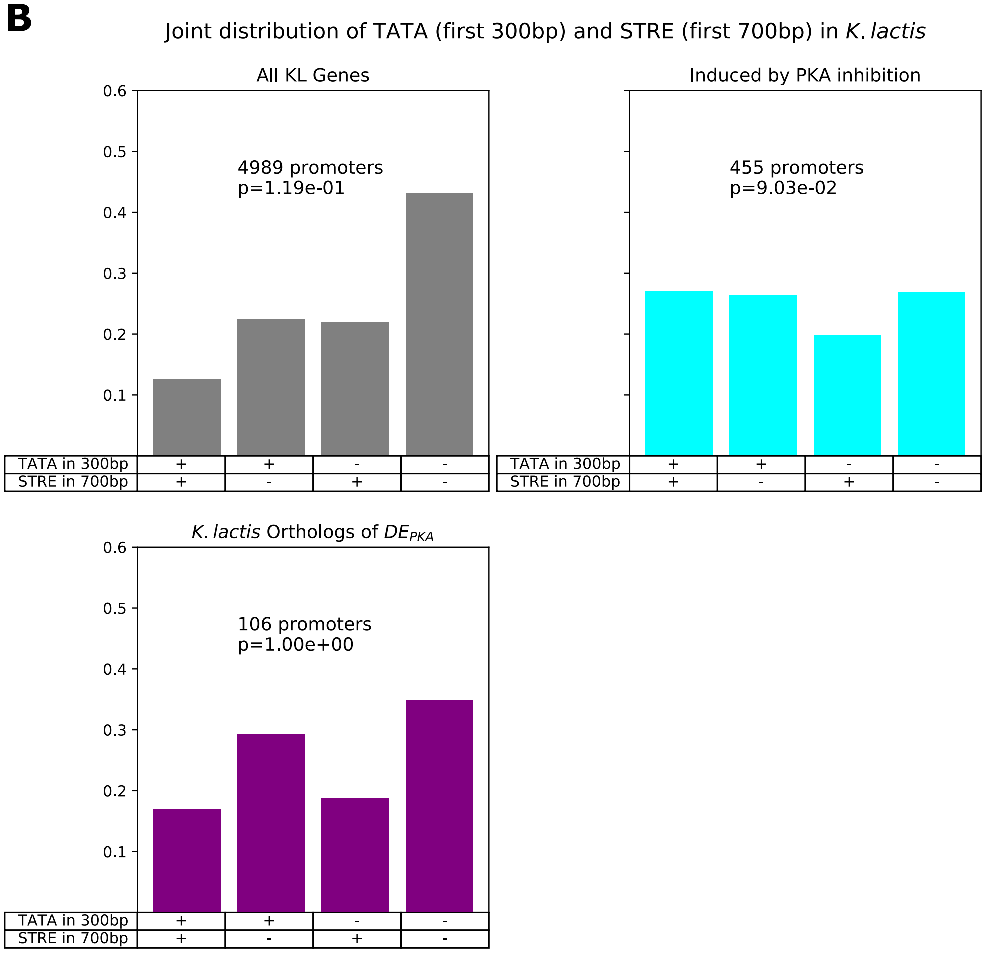
Joint distribution of TATA (first 300bp) and STRE (first 700bp) in *K. lactis*
All KL Genes
4989 promoters
p=1.19e-01
Induced by PKA inhibition
455 promoters
p=9.03e-02
K. lactis Orthologs of DE_{PKA}
106 promoters
p=1.00e+00
0.6
0.5
0.4
0.3
0.2
0.1
TATA in 300bp + + - -
STRE in 700bp + - + -

S19

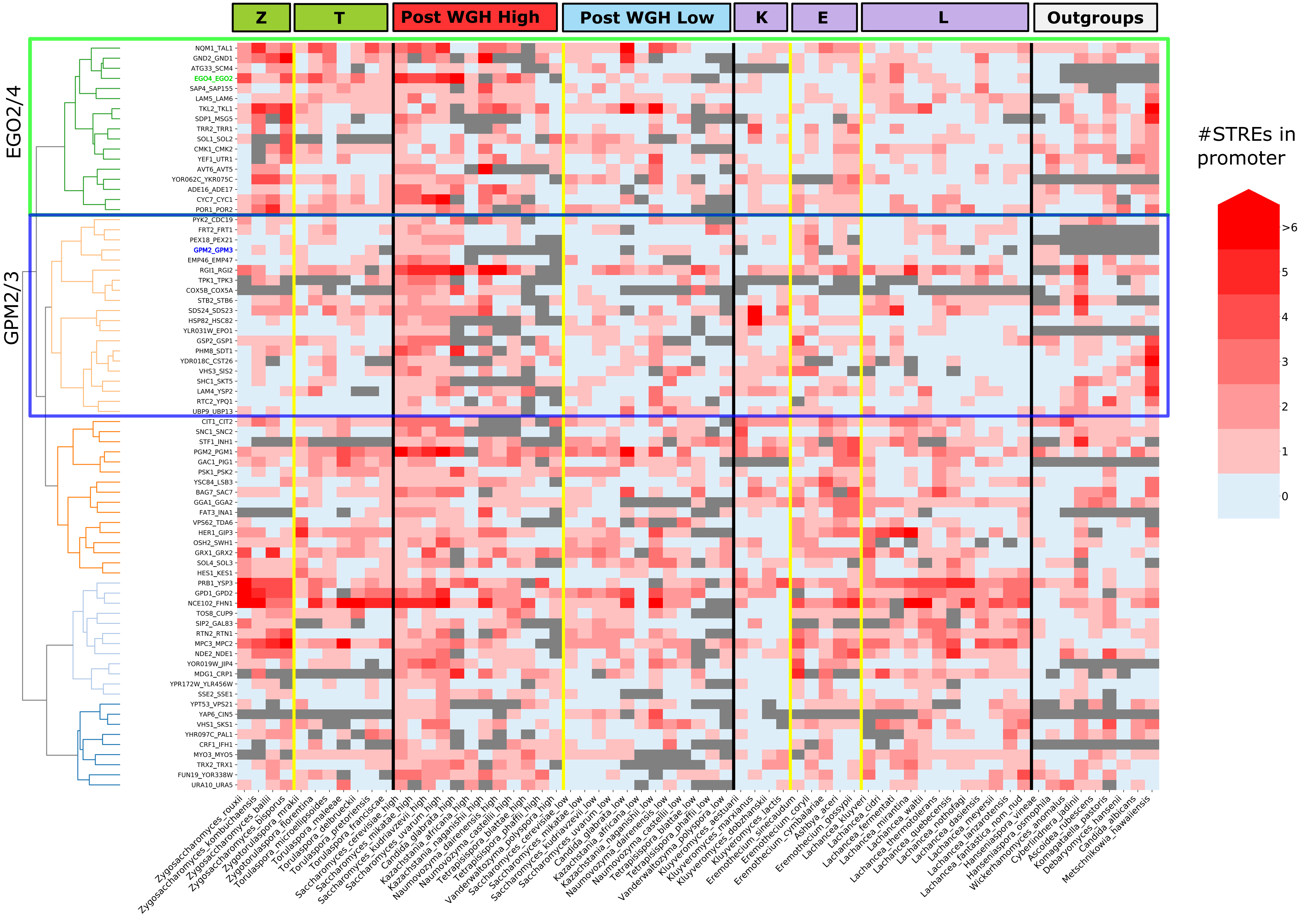

Z
T
Post WGH High
Post WGH Low
K
E
L
Outgroups
EGO2/4
GPM2/3
#STREs in promoter
>6
5
4
3
2
1
0
NQM1_TAL1
GND2_GND1
ATG33_SCM4
EGO4_EGO2
SAP4_SAP155
LAM5_LAM6
TKL2_TKL1
SDP1_MSG5
TRR2_TRR1
SOL1_SOL2
CMK1_CMK2
YEF1_UTR1
AVT6_AVT5
YOR062C_YKR075C
ADE16_ADE17
CYC7_CYC1
POR1_POR2
PYK2_CDC19
FRT2_FRT1
PEX18_PEX21
GPM2_GPM3
EMP46_EMP47
RGI1_RGI2
TPK1_TPK3
COX5B_COX5A
STB2_STB6
SDS24_SDS23
HSP82_HSC82
YLR031W_EPO1
GSP2_GSP1
PHM8_SDT1
YDR018C_CST26
VHS3_SIS2
SHC1_SKT5
LAM4_YSP2
RTC2_YPQ1
UBP9_UBP13
CIT1_CIT2
SNC1_SNC2
STF1_INH1
PGM2_PGM1
GAC1_PIG1
PSK1_PSK2
YSC84_LSB3
BAG7_SAC7
GGA1_GGA2
FAT3_INA1
VPS62_TDA6
HER1_GIP3
OSH2_SWH1
GRX1_GRX2
SOL4_SOL3
HES1_KES1
PRB1_YSP3
GPD1_GPD2
NCE102_FHN1
TOS8_CUP9
SIP2_GAL83
RTN2_RTN1
MPC3_MPC2
NDE2_NDE1
YOR019W_JIP4
MDG1_CRP1
YPR172W_YLR456W
SSE2_SSE1
YPT53_VPS21
YAP6_CIN5
VHS1_SKS1
YHR097C_PAL1
CRF1_IFH1
MYO3_MYO5
TRX2_TRX1
FUN19_YOR338W
URA10_URA5
Zygosaccharomyces_rouxii
Zygosaccharomyces_kombuchaensis
Zygosaccharomyces_bailii
Zygosaccharomyces_bisporus
Zygotorulaspora_mrakii
Zygotorulaspora_florentina
Torulaspora_microellipsoides
Torulaspora_maleeae
Torulaspora_delbrueckii
Torulaspora_pretoriensis
Torulaspora_franciscae
Saccharomyces_cerevisiae_high
Saccharomyces_mikatae_high
Saccharomyces_kudriavzevii_high
Saccharomyces_uvarum_high
Candida_glabrata_high
Kazachstania_africana_high
Kazachstania_naganishii_high
Naumovozyma_dairenensis_high
Naumovozyma_castellii_high
Tetrapisispora_blattae_high
Tetrapisispora_phaffii_high
Vanderwaltozyma_polyspora_high
Saccharomyces_cerevisiae_low
Saccharomyces_mikatae_low
Saccharomyces_kudriavzevii_low
Saccharomyces_uvarum_low
Candida_glabrata_low
Kazachstania_africana_low
Kazachstania_naganishii_low
Naumovozyma_dairenensis_low
Naumovozyma_castellii_low
Tetrapisispora_blattae_low
Tetrapisispora_phaffii_low
Vanderwaltozyma_polyspora_low
Kluyveromyces_aestuarii
Kluyveromyces_marxianus
Kluyveromyces_dobzhanskii
Kluyveromyces_lactis
Eremothecium_sinecaudum
Eremothecium_coryli
Eremothecium_cymbalariae
Ashbya_aceri
Eremothecium_gossypii
Lachancea_kluyveri
Lachancea_cidri
Lachancea_fermentati
Lachancea_mirantina
Lachancea_waltii
Lachancea_thermotolerans
Lachancea_quebecensis
Lachancea_nothofagi
Lachancea_dasiensis
Lachancea_meyersii
Lachancea_lanzarotensis
Lachancea_fantastica_nom_nud
Hanseniaspora_vineae
Hanseniaspora_osmophila
Wickerhamomyces_anomalus
Cyberlindnera_jadinii
Ascoidea_rubescens
Komagataella_pastoris
Debaryomyces_hansenii
Candida_albicans
Metschnikowia_hawaiiensis

S20

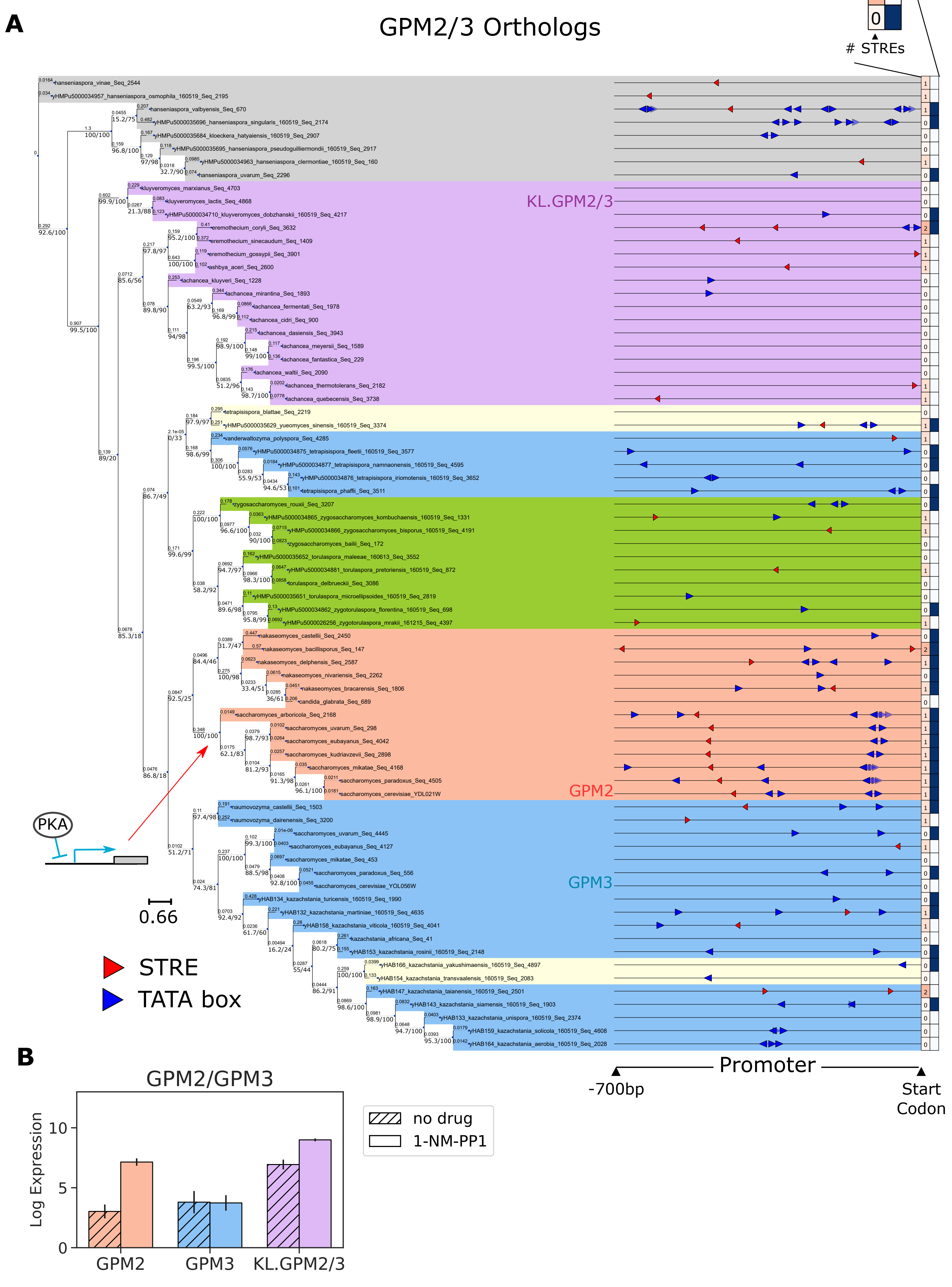
A
GPM2/3 Orthologs
TATA box in
First 300 bp
STREs
KL.GPM2/3
GPM2
GPM3
PKA
0.66
STRE
TATA box
Promoter
-700bp
Start
Codon
B
GPM2/GPM3
Log Expression
no drug
1-NM-PP1
GPM2
GPM3
KL.GPM2/3

S21

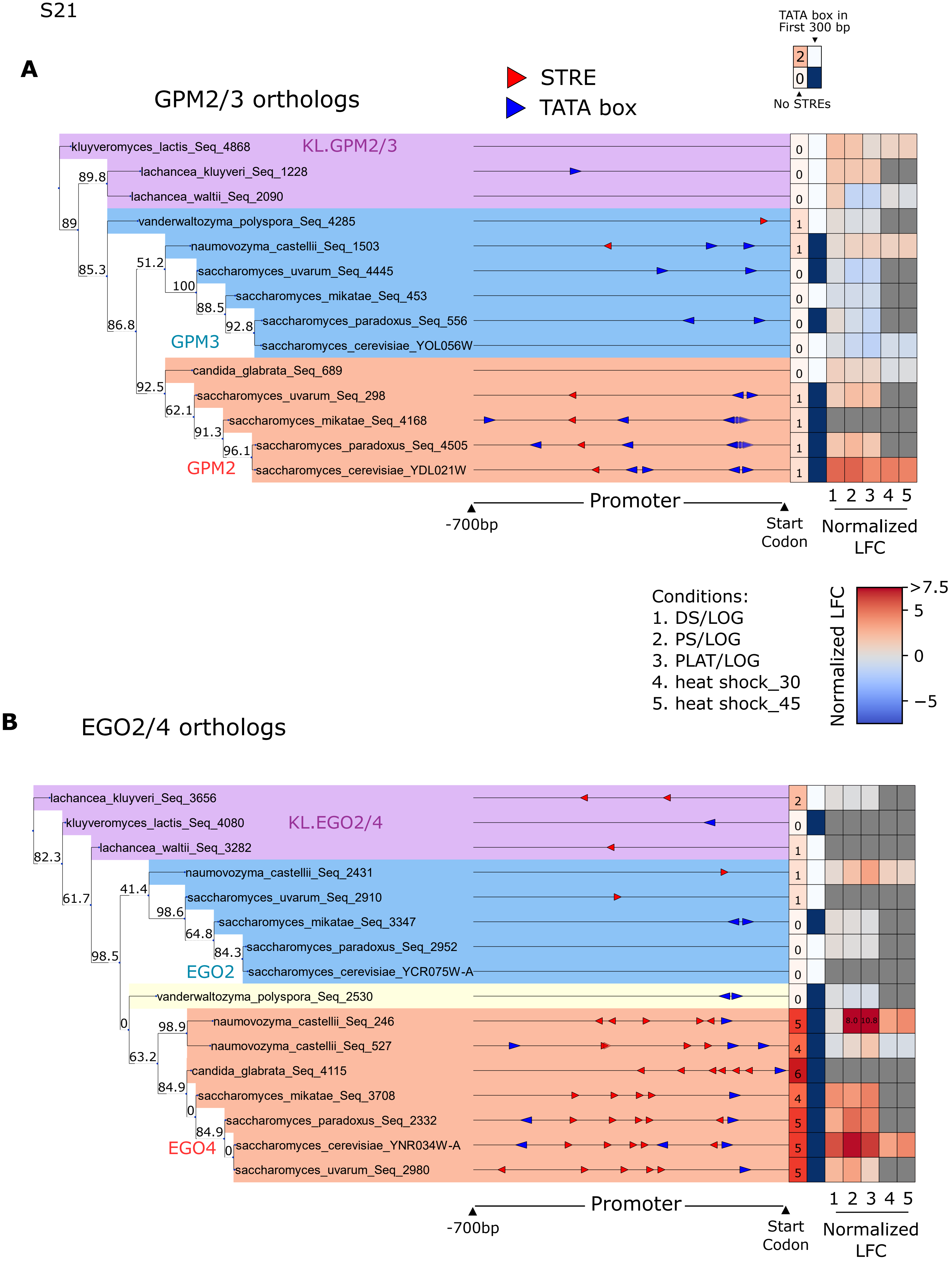
A
GPM2/3 orthologs
STRE
TATA box
TATA box in First 300 bp
No STREs
kluyveromyces_lactis_Seq_4868
lachancea_kluyveri_Seq_1228
lachancea_waltii_Seq_2090
vanderwaltozyma_polyspora_Seq_4285
naumovozyma_castellii_Seq_1503
saccharomyces_uvarum_Seq_4445
saccharomyces_mikatae_Seq_453
saccharomyces_paradoxus_Seq_556
saccharomyces_cerevisiae_YOL056W
candida_glabrata_Seq_689
saccharomyces_uvarum_Seq_298
saccharomyces_mikatae_Seq_4168
saccharomyces_paradoxus_Seq_4505
saccharomyces_cerevisiae_YDL021W
KL.GPM2/3
GPM3
GPM2
Promoter
-700bp
Start Codon
1 2 3 4 5
Normalized LFC
Conditions:
1. DS/LOG
2. PS/LOG
3. PLAT/LOG
4. heat shock_30
5. heat shock_45
Normalized LFC
>7.5
5
0
−5
B
EGO2/4 orthologs
lachancea_kluyveri_Seq_3656
kluyveromyces_lactis_Seq_4080
lachancea_waltii_Seq_3282
naumovozyma_castellii_Seq_2431
saccharomyces_uvarum_Seq_2910
saccharomyces_mikatae_Seq_3347
saccharomyces_paradoxus_Seq_2952
saccharomyces_cerevisiae_YCR075W-A
vanderwaltozyma_polyspora_Seq_2530
naumovozyma_castellii_Seq_246
naumovozyma_castellii_Seq_527
candida_glabrata_Seq_4115
saccharomyces_mikatae_Seq_3708
saccharomyces_paradoxus_Seq_2332
saccharomyces_cerevisiae_YNR034W-A
saccharomyces_uvarum_Seq_2980
KL.EGO2/4
EGO2
EGO4
8.0
10.8
Promoter
-700bp
Start Codon
1 2 3 4 5
Normalized LFC

S22

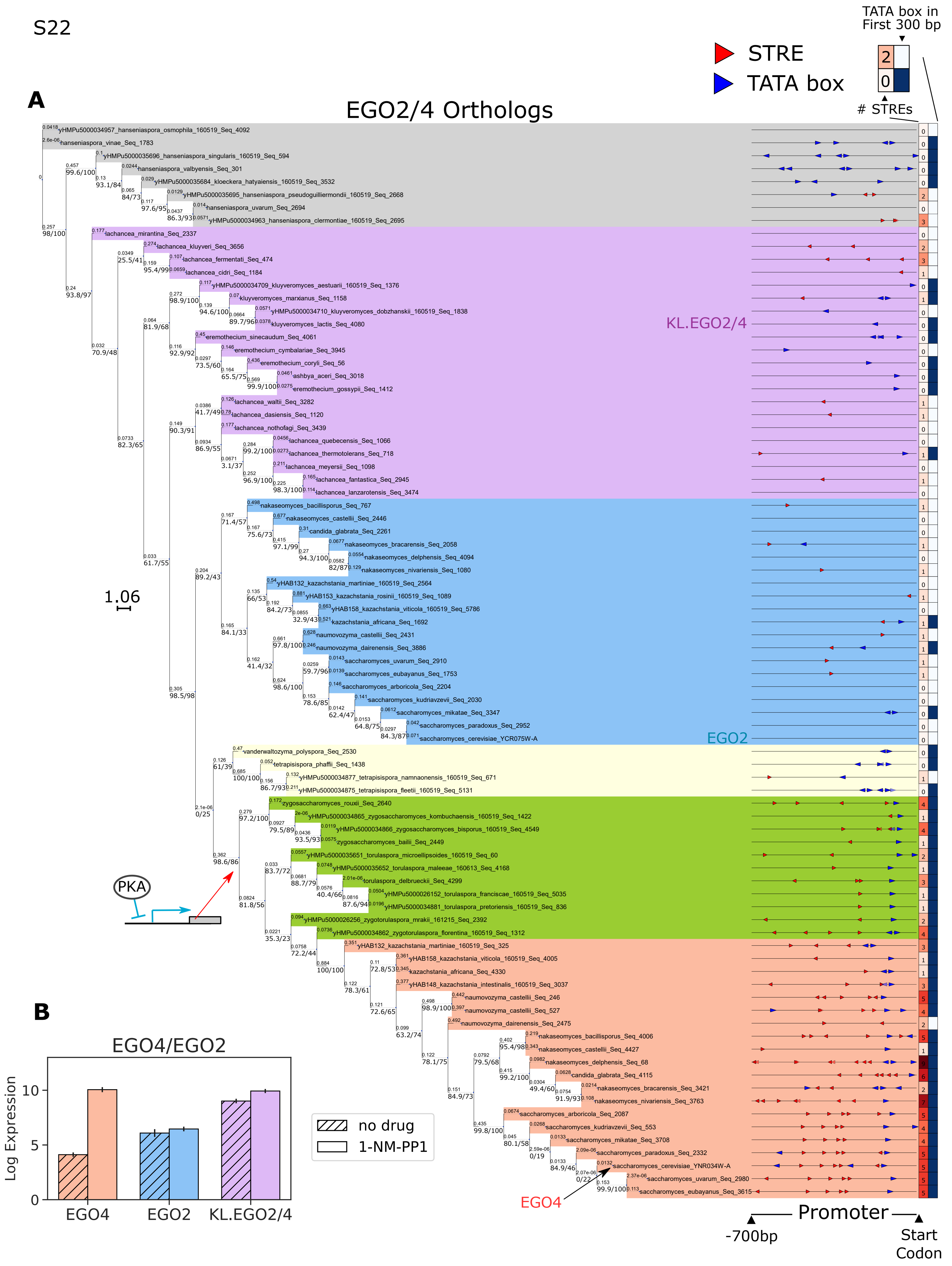

A
EGO2/4 Orthologs
STRE
TATA box
TATA box in First 300 bp
STREs
KL.EGO2/4
EGO2
EGO4
PKA
1.06
yHMPu5000034957_hanseniaspora_osmophila_160519_Seq_4092
hanseniaspora_vinae_Seq_1783
yHMPu5000035696_hanseniaspora_singularis_160519_Seq_594
hanseniaspora_valbyensis_Seq_301
yHMPu5000035684_kloeckera_hatyaiensis_160519_Seq_3532
yHMPu5000035695_hanseniaspora_pseudoguilliermondii_160519_Seq_2668
hanseniaspora_uvarum_Seq_2694
yHMPu5000034963_hanseniaspora_clermontiae_160519_Seq_2695
lachancea_mirantina_Seq_2337
lachancea_kluyveri_Seq_3656
lachancea_fermentati_Seq_474
lachancea_cidri_Seq_1184
yHMPu5000034709_kluyveromyces_aestuarii_160519_Seq_1376
kluyveromyces_marxianus_Seq_1158
yHMPu5000034710_kluyveromyces_dobzhanskii_160519_Seq_1838
kluyveromyces_lactis_Seq_4080
eremothecium_sinecaudum_Seq_4061
eremothecium_cymbalariae_Seq_3945
eremothecium_coryli_Seq_56
ashbya_aceri_Seq_3018
eremothecium_gossypii_Seq_1412
lachancea_waltii_Seq_3282
lachancea_dasiensis_Seq_1120
lachancea_nothofagi_Seq_3439
lachancea_quebecensis_Seq_1066
lachancea_thermotolerans_Seq_718
lachancea_meyersii_Seq_1098
lachancea_fantastica_Seq_2945
lachancea_lanzarotensis_Seq_3474
nakaseomyces_bacillisporus_Seq_767
nakaseomyces_castellii_Seq_2446
candida_glabrata_Seq_2261
nakaseomyces_bracarensis_Seq_2058
nakaseomyces_delphensis_Seq_4094
nakaseomyces_nivariensis_Seq_1080
yHAB132_kazachstania_martiniae_160519_Seq_2564
yHAB153_kazachstania_rosinii_160519_Seq_1089
yHAB158_kazachstania_viticola_160519_Seq_5786
kazachstania_africana_Seq_1692
naumovozyma_castellii_Seq_2431
naumovozyma_dairenensis_Seq_3886
saccharomyces_uvarum_Seq_2910
saccharomyces_eubayanus_Seq_1753
saccharomyces_arboricola_Seq_2204
saccharomyces_kudriavzevii_Seq_2030
saccharomyces_mikatae_Seq_3347
saccharomyces_paradoxus_Seq_2952
saccharomyces_cerevisiae_YCR075W-A
vanderwaltozyma_polyspora_Seq_2530
tetrapisispora_phaffii_Seq_1438
yHMPu5000034877_tetrapisispora_namnaonensis_160519_Seq_671
yHMPu5000034875_tetrapisispora_fleetii_160519_Seq_5131
zygosaccharomyces_rouxii_Seq_2640
yHMPu5000034865_zygosaccharomyces_kombuchaensis_160519_Seq_1422
yHMPu5000034866_zygosaccharomyces_bisporus_160519_Seq_4549
zygosaccharomyces_bailii_Seq_2449
yHMPu5000035651_torulaspora_microellipsoides_160519_Seq_60
yHMPu5000035652_torulaspora_maleeae_160613_Seq_4168
torulaspora_delbrueckii_Seq_4299
yHMPu5000026152_torulaspora_franciscae_160519_Seq_5035
yHMPu5000034881_torulaspora_pretoriensis_160519_Seq_836
yHMPu5000026256_zygotorulaspora_mrakii_161215_Seq_2392
yHMPu5000034862_zygotorulaspora_florentina_160519_Seq_1312
yHAB132_kazachstania_martiniae_160519_Seq_325
yHAB158_kazachstania_viticola_160519_Seq_4005
kazachstania_africana_Seq_4330
yHAB148_kazachstania_intestinalis_160519_Seq_3037
naumovozyma_castellii_Seq_246
naumovozyma_castellii_Seq_527
naumovozyma_dairenensis_Seq_2475
nakaseomyces_bacillisporus_Seq_4006
nakaseomyces_castellii_Seq_4427
nakaseomyces_delphensis_Seq_68
candida_glabrata_Seq_4115
nakaseomyces_bracarensis_Seq_3421
nakaseomyces_nivariensis_Seq_3763
saccharomyces_arboricola_Seq_2087
saccharomyces_kudriavzevii_Seq_553
saccharomyces_mikatae_Seq_3708
saccharomyces_paradoxus_Seq_2332
saccharomyces_cerevisiae_YNR034W-A
saccharomyces_uvarum_Seq_2980
saccharomyces_eubayanus_Seq_3615
Promoter
-700bp
Start Codon
B
EGO4/EGO2
Log Expression
0
5
10
EGO4
EGO2
KL.EGO2/4
no drug
1-NM-PP1

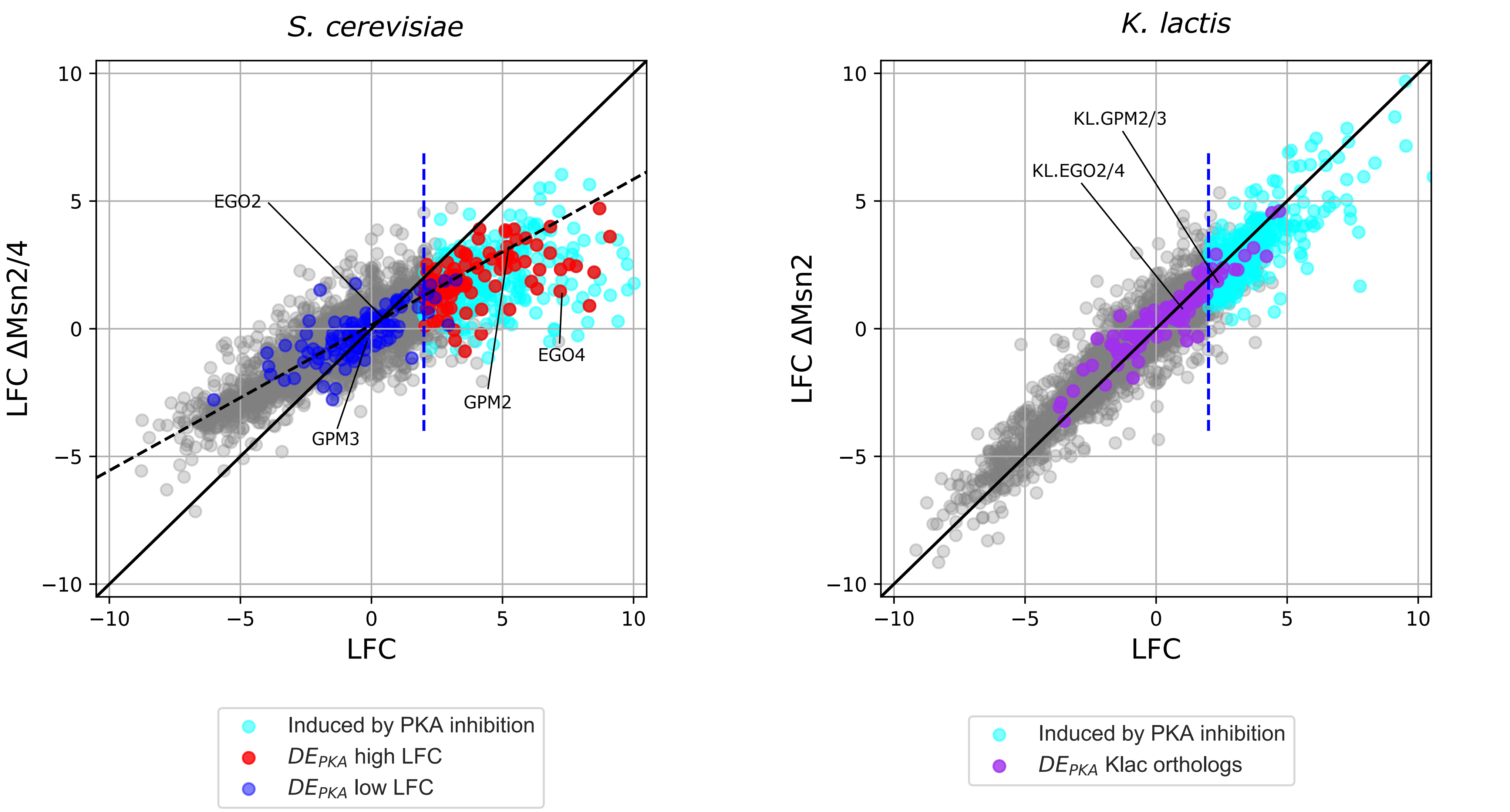
S. cerevisiae
K. lactis
LFC ΔMsn2/4
LFC ΔMsn2
LFC
EGO2
EGO4
GPM2
GPM3
KL.GPM2/3
KL.EGO2/4
Induced by PKA inhibition
DE_PKA high LFC
DE_PKA low LFC
DE_PKA Klac orthologs
