# Supplementary material for: Paralogs in the PKA Regulon Traveled Different Evolutionary Routes to Divergent Expression in Budding Yeast
Source: Front Fungal Biol. 2021 Apr 27;2:642336. doi: 10.3389/ffunb.2021.642336 (PMC10512328; doi:10.3389/ffunb.2021.642336)
Supplement: Supplementary file 2 [file Data_Sheet_2.docx]

Supplementary Material

# Supplemental Information

## Extended GO term analysis of DE_PKA_ genes.

To gain more insight into the functional characteristics of the DE_PKA_ genes, we conducted GO term analysis using the GO-slim terms for *S. cerevisiae* (Figure S5, Table S4). Although most GO annotations were shared between the paralogs, we analyzed the DE_PKA_ low-LFC and the PKA-responsive DE_PKA_ high-LFC genes separately.

With respect to Biological Process GO terms, the the DE_PKA_ low-LFC genes were enriched with a 5% FDR for several GO terms related to metabolism (‘nucleobase-containing small molecule metabolic process’, p=3.0e-7; ‘generation of precursor metabolites and energy, p=8.0e-4; and ‘cofactor metabolic process’, p=8.8e-4) and for ‘lipid transport’ (p=1.5e-5) when compared to all genes in *S. cerevisiae* for which we had RNA-seq data in. In the DE_PKA_ high-LFC genes the same terms were enriched, except of ‘cofactor metabololic process’ (p=9.1e-3) which was slightly below our FDR threshold, and with the addition of ‘endocytosis’ (p=9.5e-4). Much of this enrichment is not unique to the DE_PKA_ genes, but is shared with all ohnologs; all of these terms except ‘cofactor metabolic process’ were also enriched in ohnologs compared to all genes. When compared to just the ohnologs, just ‘nucleobase-containing small molecule metabolic process’ (p=1.8e-4) was enriched in DE_PKA_ low-LFC genes, although it was only slightly below our FDR threshold in the DE_PKA_ high-LFC genes (p=8.2e-3). Both low-LFC and high-LFC DE_PKA_ genes were de-enriched for ‘cytoplasmic translation’ (p=1.3e-5 and 1.1e-5) relative to all ohnologs, as expected given the abundance of ribosomal paralog pairs that are both repressed under PKA inhibition (Fig S4).

In order to get better resolution on the involvement of DE_PKA_ genes in metabolism, we projected all DE_PKA_ genes on to the metabolic map of budding yeast using iPath3 (Darzi et al. 2018) (Fig S6). DE_PKA_ genes were concentrated in central carbon metabolism, including glycolysis/gluconeogenesis, the Pentose Phosphate Pathway (PPP), the TCA cycle, and oxidative phosphorylation, consistent with observations of differential expression of many of these same ohnolog pairs in response to growth to saturation which developed following the WGH (Thompson et al. 2013). This included differential regulation of the Glutamate Dehydrogenases (GDH1/3) which tailor the conversion of α-ketoglutarate to glutamate to fermentative (GDH1) or respiratory (GDH3) conditions (Campero‐Basaldua et al. 2017).

The Pentose Phosphate pathway in particular contained a large proportion of DE_PKA_ ohnolog pairs. of the 12 enzymes in the pathway (Stincone et al. 2015), 8 were DE_PKA_ genes (GND1/2, SOL3/4, TKL1/2, and TAL1/NQM1). The PPP plays a role in preventing oxidative stress during respiratory conditions (Grüning et al. 2011), a function that is also shared by several other paralog pairs in DE_PKA_, including TRX1/2, GRX1/2, GCY1/YPR1, and TRR1/2. The peroxisome also plays an important role in maintaining redox homeostasis in the cell and three paralog pairs from DE_PKA_ were annotated with the ‘peroxisomal organization’ go term (PEX18/PEX21, PEX30/PEX31, and RTN1/2). The PPP also generates D-ribose 5-phosphate, a key upstream metabolite in nucleotide biosynthesis, and so it is interesting that DE_PKA_ also contains crucial enzymes in the purine and pyrimidine biosynthesis pathways (URA5/10 and ADE15/17).

Other DE_PKA_ genes were not metabolic enzymes, but were related to metabolic pathways. This included several whose molecular function annotation included ‘transmembrane transport activity’. In this set there were transporters responsible for glucose import (HXT3/5 and HXT4/7), pyruvate flux from glycolysis to the mitochondria for the TCA cycle (MPC2/3), mitochondrial ion transport (POR1/POR2), acetate and ammonia export(ADY2/ATO2), amino acid import (CAN1/ALP1), intracellular amino acid transport to the vacuole (YPQ1/RTC2), and more.

Several regulatory proteins are also included in DE_PKA_ including kinases, phosphatases, transcription factors, regulators of enzyme activity and others. Many of these are important in regulating metabolism, including TPK1/3, two of three isoforms of the PKA catalytic subunits. This list also includes GAL83/SIP2, two of the three beta subunits of the carbon-source responsive SNF1 kinase complex; USV1/RGM1, two C2H2 zinc finger transcription factors whose DNA binding specificity overlaps with that of MSN2/4 (Siggers et al. 2014); RGT2/SNF3 which are transmembrane proteins which sense and regulate glucose transport; and INH1/STF1 which inhibit ATP synthase.

Ohnolog pairs annotated with the term ‘endocytosis’ were enriched in DE_PKA_ high genes, and ohnolog pairs annotated with this term often shared the terms ‘golgi vesicle transport’ or ‘lipid transport’, indicating that reconfiguring the cell’s ability to exchange nutrients from the environment and respond to external stimuli is an important part of responding to PKA inhibition.

Although 16.2% and 28.7% of DE_PKA_ genes do not have Biological Process and Molecular Function annotations respectively, this is not significantly different than the 16.1% and 30.0% of genes lacking annotations in all genes for which we had RNA-seq data.

## Extended analysis of promoter sequences.

To identify bioinformatic signals associated with the promoters of all genes activated by PKA inhibition in *S. cerevisiae,* we used the DREME algorithm (see Materials and Methods) which identifies short ungapped motifs that are enriched in comparison to a background set of promoters in (Bailey 2011). Comparing the promoters of all genes activated under PKA inhibition in *S. cerevisiae* against the promoters of all *S. cerevisiae* genes, we identified a motif that strongly resembled the Stress Response Element (STRE, CCCCT) (Fig 4A), the binding sequence for Msn2 and Msn4 (Görner et al. 2002; Smith 1998). Four of the other five motifs enriched in the promoters of genes activated by PKA inhibition were similar to the STRE or the Post Diauxic Shift element (PDS) motif (T(A/T)AGGGAT) which is itself structurally similar to the STRE (Pedruzzi et al. 2000), while others resembled the TATA-box (TATA(A/T)A(A/T)(A/G)) (E-value 1.3e-3) which is known to be enriched in stress responsive promoters (Basehoar, Zanton, and Pugh 2004) .

Next, we asked how the number and locations of STRE and TATA-box motifs correlated with a gene being responsive to PKA inhibition. Promoters of genes activated by PKA had a larger probability of containing one or more STREs relative to all promoters in *S. cerevisiae* (76.2% vs. 44.8%, p-value 1.8e-17). They also had a notable increase in the average number of STREs per promoter (1.35 vs. 0.63) (Fig 4B). Furthermore, the location of the STREs in the promoters of the genes induced by PKA inhibition had a unimodal distribution with 63.7% of STRE sites found between 100 and 400 base pairs, as opposed to an expectation of 42.9% from a uniform distribution and a 46.7% value when the distribution of STRE locations is compiled for all promoters in the genome (Fig S16A).

The promoters of PKA targets were also enriched for the TATA-box (71.2% with 1 or more TATA box v.s. 58.5% in the promoters of all genes, p-value 2.1e-3) (Fig S17A). We observed a similar clustering of binding sites close to the start codon for TATA box motifs as we saw for STRE motifs with (54.6% of TATA-box motifs found in the first 250 base pairs in the promoters of PKA targets versus 49.3% for the promoters of all genes) (Fig S17C). Finally, a TATA box within the first 300 base pairs is more likely to occur together with an STRE motif in promoters of genes activated by PKA inhibition than in all genes (42.0% of promoters vs. 19.0%), as expected from the increased percentages of both STREs and TATA boxes in genes activated by PKA inhibition (Fig S18A).

For *K. lactis*, the top hit for promoters of genes activated by PKA inhibition was a motif whose Position Specific Scoring Matrix (PSSM) would be satisfied by an STRE but was closer to a PDS (E-value 1.3e-7) (Fig 4A). Furthermore, the bioinformatic signal for the number of STREs and their location was weaker in *K. lactis* than in *S. cerevisiae* (46.8% of promoters with 1 or more STRE in the promoter in PKA activated genes vs. 34.5% in all genes, p=1.5e-4) (Fig 4B). In *K. lactis*, as in *S. cerevisiae*, the promoters of genes activated by PKA were enriched for TATA boxes (68.1% with 1 or more TATA box vs. 53.8% in all genes, p=9.1e-4) (Fig S17B). Location clustering in the promoters of genes activated by PKA inhibition for STRE motifs was not apparent in K. lactis (Fig S16B), but it was for the TATA box (Fig S17D)

The number of TATA-boxes in the promoters of the DE_PKA_ high-LFC ohnologs were also increased relative to those of the promoters of all genes. However, unlike for the STRE, this enrichment was also present for DE_PKA_ low-LFC ohnologs and the *K. lactis* orthologs of the DE_PKA_ genes (Fig S17A,B). Based on that observation, we reasoned that, at least in the context of the DE_PKA_ genes and their orthologs, the TATA box was not linked strongly enough to induction following PKA inhibition and was likely to be an ambiguous evolutionary signal. Therefore, we focused instead on the presence of STREs as a bioinformatic proxy for gene induction in response to PKA inhibition.

## Details on Yeast Transformation Methods

Auxotrophy for URA in the *K. lactis* WT strain (yBMH128) was made by subjecting the strain yLB13a to counterselection on 5-FOA and confirmed by sequencing.

The *K. lactis* PKA-AS strains (yBMH132, yBMH078), containing the M139G and M222G mutations for KL.TPK2 and KL.TPK3 respectively, were constructed using a single plasmid CRISPR strategy based on (Ryan and Cate 2014). Cas9 and sgRNA expression constructs were combined using Gibson Assembly on a backbone with a *K. lactis* autonomously replicating sequence that allows plasmid replication in a variety of budding yeast species (Liachko and Dunham 2014). The guide targeting sequence was changed using Gibson assembly to combine PCR products containing a new guide sequence with the digested backbone. Donor constructs had at least 300 bp of homology upstream and downstream from the point mutation, as well as a heterology block consisting of synonymous mutations in the location of the sgRNA target to prevent re-cutting by the Cas9/sgRNA complex as described in (Horwitz et al. 2015). The donor cassette was printed by SGI-DNA, inc. and integrated into a PUCGA 1.0 backbone.

The transformation for the CRISPR/Cas9 point mutations for the *K.lactis* PKA-AS strain used for RNA-seq and growth experiments (yBMH132) was performed using a standard Lithium Acetate protocol designed for transformations in *S. cerevisiae* based on (Lee et al. 2015) with the following adjustments. 4ml of cells were used for each transformation. The DNA mix contained 5µg Donor DNA PCR amplified and column purified from the Donor DNA plasmid, and 1µg guide plasmid. Colonies were picked after 3-4 days incubation at 30°C.

The transformation for the CRISPR/Cas9 point mutations for the *K.lactis* PKA-AS strain used for the KL.Msn2 nuclear localization experiment (yBMH078) was performed using the CRISPR/Cas9 transformation protocol for *S. cerevisiae* from (Ryan and Cate 2014) with the following adjustments. 7.5ml of cells/transformation at OD600 of 0.8 were used to prepare competent cells. Competent cells were washed twice in LATE buffer (100mM Lithium Acetate, 10mM Tris-HCL ph8.0, 0.1mM EDTA ph8.0) prior to resuspending in equal parts LATE buffer (with no PEG 2000) and 50% glycerol and freezing at at -80°C. Cells were washed in 1xTE buffer prior to plating on SD-URA and incubated at 37°C for 12-24 hours (instead of 48 hours) followed by 2-3 days at 30°C.

The CRISPR deletion cassettes for *S. cerevisiae* Msn2/4 deletions were constructed using the plasmids and golden gate protocol from (“Quick and easy CRISPR engineering in Saccharomyces cerevisiae · Benchling,” n.d.) which incorporates in vivo homologous recombination to complete the Cas9/sgRNA expression plasmid per (Horwitz et al. 2015). A similar set of plasmids was constructed to replace the backbone of the integration vector with the Pan-ARS backbone for use in *K.lactis* using golden gate cloning. Donor DNA for these constructs was constructed using a golden gate strategy to insert the donor sequence into the YTK095 backbone (Lee et al. 2015). The donor sequence was designed to have 60bp homology for *S. cerevisiae* and 300bp homology for *K. lactis* to delete the SC.Msn2/4 or KL.Msn2 proteins respectively. Unlike a deletion cassette strategy, we did not use selection markers and therefore deleted 250bp of the promoter of each protein targeted for deletion in addition to removing their coding sequences in order to prevent spurious expression from an active endogenous promoter. Donor inserts were built using 3 sets of annealed oligos for *S. cerevisiae* or ordered as GeneBlocks (IDT) for *K.lactis.*

Transformations for the CRISPR gene deletions of Msn2/4 in *S. cerevisiae* (yBMH168, yBMH170) and KL.Msn2 in *K. lactis* (yBMH201) were performed using the same standard Lithium Acetate protocol as for yBMH132. The DNA mix for *S. cerevisiae* contained 20ng of BsmBI digested and column purified Cas9/sgRNA expression vector, 40ng of EcoRV digested and column purified sgRNA insertion vector for each mutation (Msn2 and Msn4), and 400ng PCR amplified and column purified donor DNA for each mutation. The DNA mix for *K.lactis* contained 100ng BsaI digested and column purified Cas9/sgRNA expression vector, 200ng of EcoRV digested and column purified sgRNA insertion vector, and 2µg PCR amplified and column purified donor DNA for each mutation.

Following verification of CRISPR point mutations and deletions by sequencing, the Cas9-sgRNA plasmids and expression vectors were removed using counterselection on 5-FOA.

Plasmids for Msn2(C649S) and KL.Msn2(C623S) fluorescent reporters were constructed using restriction digestion and ligation of PCR products. The point mutations that ablate DNA binding for these transcription factors were made using quick change mutagenesis. In addition to containing an mCherry fluorescent reporter for their endogenous Msn2 transcription factors, each strain carried a Venus fluorescent reporter for the Msn2 transcription factor from the opposite species which was not analyzed for this study.

Transformations for the *S. cerevisiae* Msn2 nuclear localization strains (yEW051 and yEW052) were done using a similar lithium acetate protocol as for yBMH132 except for the following variations. An initial amount of 2ml of cells at OD600 of 0.6 were used, the pellet was washed and resuspended in LATE buffer and 30 µl of cell resuspension was combined with 2µl salmon sperm DNA, 120µl 50% PEG-3350, 30µl LITE and 2-5µg digested integration plasmid in 10µl water. Following heat shock cells were washed with 10mM Tris-HCL ph8.0, 0.1mM EDTA ph8.0 (TE) buffer and plated on selective media.

Transformations for the *K. lactis* Msn2 Nuclear localization markers were performed using an electroporation procedure based on that described in (Kooistra and Steensma 2003) with the following variations. Initially 50ml of OD 0.8 cells were used, wash and DTT buffer volumes were halved, and the volume of final resuspension in electroporation buffer was 240µl. For each transformation, 60µl resuspended cells, originating from about 12.5ml OD 0.8 cell suspension, were mixed with 5µl ssDNA, and 10-20µl DNA mix prior to electroporation, recovery, and plating. DNA mix consisted of 1.5µg cut and column purified integration plasmids in water.

# Supplementary References

Bailey, Timothy L. 2011. ‘DREME: Motif Discovery in Transcription Factor ChIP-Seq Data’. *Bioinformatics (Oxford, England)* 27 (12): 1653–59. https://doi.org/10.1093/bioinformatics/btr261.

Basehoar, Andrew D, Sara J Zanton, and B. Franklin Pugh. 2004. ‘Identification and Distinct Regulation of Yeast TATA Box-Containing Genes’. *Cell* 116 (5): 699–709. https://doi.org/10.1016/S0092-8674(04)00205-3.

Byrne, Kevin P., and Kenneth H. Wolfe. 2005. ‘The Yeast Gene Order Browser: Combining Curated Homology and Syntenic Context Reveals Gene Fate in Polyploid Species’. *Genome Research* 15 (10): 1456–61. https://doi.org/10.1101/gr.3672305.

Campero‐Basaldua, Carlos, Héctor Quezada, Lina Riego‐Ruíz, Dariel Márquez, Erendira Rojas, James González, Mohammed El‐Hafidi, and Alicia González. 2017. ‘Diversification of the Kinetic Properties of Yeast NADP-Glutamate-Dehydrogenase Isozymes Proceeds Independently of Their Evolutionary Origin’. *MicrobiologyOpen* 6 (2): e00419. https://doi.org/10.1002/mbo3.419.

Darzi, Youssef, Ivica Letunic, Peer Bork, and Takuji Yamada. 2018. ‘IPath3.0: Interactive Pathways Explorer V3’. *Nucleic Acids Research* 46 (W1): W510–13. https://doi.org/10.1093/nar/gky299.

Görner, Wolfram, Erich Durchschlag, Julia Wolf, Elizabeth L. Brown, Gustav Ammerer, Helmut Ruis, and Christoph Schüller. 2002. ‘Acute Glucose Starvation Activates the Nuclear Localization Signal of a Stress-Specific Yeast Transcription Factor’. *The EMBO Journal* 21 (1–2): 135–44. https://doi.org/10.1093/emboj/21.1.135.

Grüning, Nana-Maria, Mark Rinnerthaler, Katharina Bluemlein, Michael Mülleder, Mirjam M.C. Wamelink, Hans Lehrach, Cornelis Jakobs, Michael Breitenbach, and Markus Ralser. 2011. ‘Pyruvate Kinase Triggers a Metabolic Feedback Loop That Controls Redox Metabolism in Respiring Cells’. *Cell Metabolism* 14 (3): 415–27. https://doi.org/10.1016/j.cmet.2011.06.017.

Horwitz, Andrew A., Jessica M. Walter, Max G. Schubert, Stephanie H. Kung, Kristy Hawkins, Darren M. Platt, Aaron D. Hernday, et al. 2015. ‘Efficient Multiplexed Integration of Synergistic Alleles and Metabolic Pathways in Yeasts via CRISPR-Cas’. *Cell Systems* 1 (1): 88–96. https://doi.org/10.1016/j.cels.2015.02.001.

Kooistra, Rolf A., and H. Yde Steensma. 2003. ‘Transformation of Kluyveromyces Lactis’. In *Non-Conventional Yeasts in Genetics, Biochemistry and Biotechnology: Practical Protocols*, edited by Klaus Wolf, Karin Breunig, and Gerold Barth, 169–74. Springer Lab Manuals. Berlin, Heidelberg: Springer Berlin Heidelberg. https://doi.org/10.1007/978-3-642-55758-3_26.

Lee, Michael E., William C. DeLoache, Bernardo Cervantes, and John E. Dueber. 2015. ‘A Highly Characterized Yeast Toolkit for Modular, Multipart Assembly’. *ACS Synthetic Biology* 4 (9): 975–86. https://doi.org/10.1021/sb500366v.

Liachko, Ivan, and Maitreya J. Dunham. 2014. ‘An Autonomously Replicating Sequence for Use in a Wide Range of Budding Yeasts’. *FEMS Yeast Research* 14 (2): 364–67. https://doi.org/10.1111/1567-1364.12123.

Pedruzzi, Ivo, Niels Bürckert, Pascal Egger, and Claudio De Virgilio. 2000. ‘Saccharomyces Cerevisiae Ras/CAMP Pathway Controls Post‐diauxic Shift Element‐dependent Transcription through the Zinc Finger Protein Gis1’. *The EMBO Journal* 19 (11): 2569–79. https://doi.org/10.1093/emboj/19.11.2569.

Roy, Sushmita, Ilan Wapinski, Jenna Pfiffner, Courtney French, Amanda Socha, Jay Konieczka, Naomi Habib, Manolis Kellis, Dawn Thompson, and Aviv Regev. 2013. ‘Arboretum: Reconstruction and Analysis of the Evolutionary History of Condition-Specific Transcriptional Modules’. *Genome Research* 23 (6): 1039–50. https://doi.org/10.1101/gr.146233.112.

Ryan, Owen W., and Jamie H. D. Cate. 2014. ‘Multiplex Engineering of Industrial Yeast Genomes Using CRISPRm’. *Methods in Enzymology* 546: 473–89. https://doi.org/10.1016/B978-0-12-801185-0.00023-4.

Scannell, Devin R., A. Carolin Frank, Gavin C. Conant, Kevin P. Byrne, Megan Woolfit, and Kenneth H. Wolfe. 2007. ‘Independent Sorting-out of Thousands of Duplicated Gene Pairs in Two Yeast Species Descended from a Whole-Genome Duplication’. *Proceedings of the National Academy of Sciences of the United States of America* 104 (20): 8397–8402. https://doi.org/10.1073/pnas.0608218104.

*SGD Project*. 2018. https://downloads.yeastgenome.org/curation/literature/go_slim_mapping.tab.

Shen, Xing-Xing, Dana A. Opulente, Jacek Kominek, Xiaofan Zhou, Jacob L. Steenwyk, Kelly V. Buh, Max A. B. Haase, et al. 2018. ‘Tempo and Mode of Genome Evolution in the Budding Yeast Subphylum’. *Cell* 175 (6): 1533-1545.e20. https://doi.org/10.1016/j.cell.2018.10.023.

Siggers, Trevor, Jessica Reddy, Brian Barron, and Martha L. Bulyk. 2014. ‘Diversification of Transcription Factor Paralogs via Noncanonical Modularity in C2H2 Zinc Finger DNA Binding’. *Molecular Cell* 55 (4): 640–48. https://doi.org/10.1016/j.molcel.2014.06.019.

Smith, A. 1998. ‘Yeast PKA Represses Msn2p/Msn4p-Dependent Gene Expression to Regulate Growth, Stress Response and Glycogen Accumulation’. *The EMBO Journal* 17 (13): 3556–64. https://doi.org/10.1093/emboj/17.13.3556.

Stincone, Anna, Alessandro Prigione, Thorsten Cramer, Mirjam M. C. Wamelink, Kate Campbell, Eric Cheung, Viridiana Olin-Sandoval, et al. 2015. ‘The Return of Metabolism: Biochemistry and Physiology of the Pentose Phosphate Pathway’. *Biological Reviews of the Cambridge Philosophical Society* 90 (3): 927–63. https://doi.org/10.1111/brv.12140.

Thompson, Dawn A., Sushmita Roy, Michelle Chan, Mark P. Styczynsky, Jenna Pfiffner, Courtney French, Amanda Socha, et al. 2013. ‘Evolutionary Principles of Modular Gene Regulation in Yeasts’. *ELife* 2 (June): e00603. https://doi.org/10.7554/eLife.00603.

Tsankov, Alexander M., Dawn Anne Thompson, Amanda Socha, Aviv Regev, and Oliver J. Rando. 2010. ‘The Role of Nucleosome Positioning in the Evolution of Gene Regulation’. *PLoS Biology* 8 (7): e1000414. https://doi.org/10.1371/journal.pbio.1000414.

# Supplementary Table Captions

**Table S1**: **Go term enrichment for PKA targets.** GO term enrichment was calculated from the GO Slim Dataset downloaded from SGD on April 12^th^, 2018 (*SGD Project* 2018) and p-values were calculated using Fisher's exact test against a background of either all genes in *S. cerevisiae* (for the gene sets containing only genes activated or repressed in *S. cerevisiae*) or all genes in *S. cerevisiae* that contain a *K. lactis* ortholog (for gene sets defined by activation or repression in *K. lactis*). Except where indicated, all terms which met an FDR threshold of 5% using the BH procedure are shown*.*

**Table S2:** **Enrichment of ohnologs in genes activated or repressed by PKA inhibition in *S. cerevisiae*.** Enrichment and de-enrichment for ohnologs in *S. cerevisiae* gene sets defined by activation or repression following PKA-inhibition by the gene or by its ortholog *in K. lactis*. P-values were calculated using Fisher's exact test as in Table S1 against a background of either all genes in *S. cerevisiae* (for the gene sets containing only genes activated or repressed in *S. cerevisiae*) or all genes in *S. cerevisiae* that contain a *K. lactis* ortholog (for gene sets defined by activation or repression in *K. lactis).* Enrichment was calculated with an alternative hypothesis that the percentage of ohnologs in the given set was greater than in the background set. Calculations for de-enrichment used an alternative hypothesis that the percentage of ohnologs was less than in the background set. Adjusted p-values are calculated using the BH procedure and significance was assessed using an FDR of 5%.

**Table S3**: **DE_PKA_ list.** Gene descriptions from SGD and GO-slim terms broken down by Biological Process, Molecular Function and Cellular Component for DE_PKA_ ohnolog pairs.

**Table S4:** **GO enrichment of DE_PKA_ genes and ohnologs.** GO enrichment and de-enrichment is calculated for each of the GO-slim aspects for ohnologs vs. all genes, DE_PKA_ high and low genes vs either all genes or ohnologs. Results for each comparison are stored in separate tabs. All terms with p-value less than 0.1 are listed, and the column detect_FDR reports whether a term was significant with an FDR threshold of 5%.

**Table S5:** **List of ohnologs pairs with RNA seq data following PKA inhibition.**  Ohnologs and related data are sorted such that ohnolog 1 (blue shading) has lower LFC in response to PKA inhibition and ohnolog 2 (red shading) has higher LFC, except where the more extreme LFC was not significant according to a 1% FDR while the less extreme LFC was. Rows are sorted from highest to lowest LFC for the ohnolog 2. Purple shading indicates data pertaining to the shared *K. lactis* orthologs. Column Descriptions: DE PKA: ohnolog pair is differentially expressed in response to PKA activation with one member activated and the other not activated. Ohnolog pairs in this group are members of DE_PKA_, defined in the text; LFC categories are strong_rep (LFC<-2.0, padj<0.01), weak_rep (-2.0<LFC<-1.0, padj<0.01), weak_act (1.0<LFC<2.0, padj<0.01), strong_act (LFC>2.0 at padj<0.01), or no_change (all others); AA%id : percent identity between both ohnologs in *S. cerevisiae*; Length Ratio: ratio of shortest/longest number of amino acids between ohnologs in *S. cerevisiae*; LFC, padj, rlog control, rlog 1-NM-PP1: data from RNA seq experiments (see Methods).

**Table S6: List of strains**

**Table S7: List of plasmids**

**Table S8: Syntenic ohnolog assignment for example proteins.** The YGOB webtool was queried with a window of +/-8 genes to obtain syntenic orthologs for YGOB sequences. For post-WGH species from (Shen et al. 2018), surrounding genes were extracted and assigned orthology based on orthogroup assignments from that work. Orthology to *S. cerevisiae* and *K. lactis* genes from YGOB was used to assign a gene to a given column and then syntenic orthologs were manually assigned based on similarity to the syntenic groups for YGOB species assigned by the YGOB webtool.

# Supplementary Figure Captions

**Figure S1**: **PKA inhibition inhibits growth and causes Msn2 nuclear localization in *S. cerevisiae* and *K. lactis*.**  (A) WT and PKA-AS *S. cerevisiae* and *K. lactis* strains were grown in YPD in the presence or absence of 3um 1-NM-PP1 and OD600 was measured every 20min. Standard Deviation of at least 4 technical replicates is shown. (B) Selected microscopy images from data in Fig1B. WT and AS strains were grown in SDC and imaged either 2 minutes before or 10 minutes after adding control media or 4uM 1-NM-PP1.

**Figure S2:** **Thresholds defining genes activated and repressed by PKA inhibition in *S. cerevisiae* and *K. lactis*.** Volcano plot showing Log Fold Change (LFC) comparing RNA sequencing data collected from strains in which PKA-AS was inhibited with 3uM 1-NMPP1 versus DMSO controls plotted against the negative base10 log transform of the adjusted p-value for genes in (A) *S. cerevisiae* and (B) *K. lactis.* Targets of activation or repression in each species were defined as all genes that had LFC greater than 2.0 or less than -2.0 respectively and were significant with a False Discovery Rate of 1% (-log10(padj)<2.0).

**Figure S3:** **LFC in each species for selected GO-slim terms enriched in genes activated or repressed by PKA inhibition in *S. cerevisiae, K. lactis,* or both species.** LFC of *S. cerevisiae* (y-axis) and *K. lactis* (x-axis) comparing PKA inhibition with 3uM 1-NM-PP1 versus a DMSO control. Values are only shown for genes that were expressed and had orthologs in both species. Colored datapoints indicate all genes whose *S. cerevisiae* ortholog is a member of indicated GO-slim terms from Table S1. All other genes are colored grey.

**Figure S4**: **LFC of ohnolog pairs for selected GO-slim terms.** LFC of ohnolog pairs in *S. cerevisiae* sorted as in Figure 2A with ohnolog pairs associated with indicated GO-slim terms highlighted.

**Figure S5: Visualization of select GO-slim terms for DE_PKA_ genes.** The presence of selected GO-slim terms (listed on the x-axis) are indicated for each DE_PKA_ ohnolog pair (y-axis) in grey. There are two columns for each go term for the low-LFC ohnolog (left) and high-LFC ohnolog. The black squares in the column on the left indicates presence of the ohnolog pair in the *S. cerevisiae* metabolism map from iPath 3.0 (Darzi et al. 2018)*.* Ohnolog pairs are clustered based on shared presence of GO-terms, and in the *S. cerevisiae* metabolism map (Methods). GO-term abbreviations: Biological Process, PM: 'generation of precursor metabolites and energy', NM: 'nucleobase-containing small molecule metabolic process', CM: 'cofactor metabolic process’, CR: 'cellular respiration', OX: 'response to oxidative stress', PO: 'peroxisome organization', LT: 'lipid transport', EC: 'endocytosis', CW: 'cell wall organization or biogenesis', GV: 'Golgi vesicle transport', S: 'signaling', UNK: biological process unknown. Cellular Component, CC: 'cell cortex', PM: 'plasma membrane', M: 'mitochondrion', P: ‘peroxisome’, N: ‘nucleus’, UNK: cellular component unknown. Molecular Function, OA: 'oxidoreductase activity', TT: 'transmembrane transporter activity', LB: ‘lipid binding', KA: ‘kinase activity', TF: 'nucleic acid binding transcription factor activity', RA: 'enzyme regulator activity', PP: 'phosphatase activity', UNK: molecular function unknown.

**Figure S6: DE_PKA_ genes are involved in central carbon metabolism.** Metabolic reactions performed by DE_PKA_ ohnologs are highlighted in red on a background of the *S. cerevisiae* metabolic network from iPath 3.0 (Darzi et al. 2018). Reactions performed by other WGH ohnologs are highlighted in blue.

**Figure S7: Basal expression (rlog) for DE_PKA_ low and high LFC ohnologs and their shared orthologs in K. lactis.** Average rlog data from PKA-AS strains with no drug during exponential growth are shown. Gray boxes indicate missing orthologs in K. lactis.

**Figure S8: The stress conditions Diauxic Shift (DS), Post Diauxic Shift (PS), Plateau (PLAT) from Thompson et al. 2013, and heat shock at 30 and 45 minutes from Roy et al. 2013 are most closely correlated with PKA inhibition in both *K. lactis* and *S. cerevisiae*.** Pearson’s correlation coefficient between normalized LFC for the indicated condition from (Thompson et al. 2013) or (Roy et al. 2013) and our PKA inhibition data is plotted for *S. cerevisiae* (x-axis) and *K.Lactis* (y-axis).

**Figure S9: Induction of DE_PKA_ orthologs in response to stresses related to PKA inhibition.** Normalized LFC values from the PKA-related stress conditions from Figure S6 are shown for the orthologs of each paralog pair in DE_PKA_. Yellow lines separate non-WGH species (on the right) and post-WGH species (two groups on the left). Syntenic orthologs of high-LFC ohnologs are on the left, and syntenic orthologs of low-LFC ohnologs are in the center. Where there are three columns, the conditions are 'DS/LOG', 'PS/LOG', and 'PLAT/LOG' from (Thompson et al. 2013) and where there are five bars, the conditions are those three conditions plus 'heat shock_030' and 'heat shock_045' from (Roy et al. 2013). *S. pombii* had the three growth conditions and ‘heat shock_30’. The rows are sorted such that the average LFC across conditions in the syntenic orthologs of the high-LFC ohnolog is more conserved towards the top and less conserved towards the bottom. The ohnolog pairs (YDR034W-B, YBR056W-A), (YCL048W-A, YDR524C-B), (CIS1, YGR035C), (EGO4, EGO2), and (YOR186W, YLR297W) in DE_PKA_ did not have sufficient data in the microarray experiments to be included in the analysis.

**Figure S10: Basal expression from RNA-seq experiments is correlated with basal expression data from** (Tsankov et al. 2010)**.** (A) rlog data from a PKA-AS under exponential growth in YPD with no drug is shown on the x-axis, and normalized basal expression from (Tsankov et al. 2010) (See Materials and Methods for details on normalization) is shown on the y-axis for all genes in *S. cerevisiae* (left panel) and *K. lactis* (right panel). (B) The same data as in (A) for Low-LFC (blue) and High-LFC (red) DE_PKA_ ohnologs from *S. cerevisiae* and their shared *K. lactis* orthologs (purple) are shown.

**Figure S11: Basal expression of DE_PKA_ orthologs for 11 budding yeast species.** Normalized expression data (see Materials and Methods) for each species is shown from (Tsankov et al. 2010). Rows and columns are ordered as in Fig S6.

**Figure S12: Ohnolog pairs that are differentially expressed in response to PKA-related stress conditions in *S. cerevisiae* (**${DE}_{Stress}^{Scer}$) **overlaps significantly with DE_PKA_.**  We can define ohnolog pairs that have one member activated by PKA-related stress conditions (Fig S7) and which have differential expression in a similar sense as defined for DE_PKA_, which we denote for a particular species as ${DE}_{Stress}^{Species}$ (See Materials and Methods). (A) Overlap between ${DE}_{PKA}$ ohnolog pairs (which are defined in *S. cerevisiae)* and ${DE}_{Stress}^{Scer}$. ${DE}_{Stress}$ohnolog pairs were defined in each species based on the average normalized LFC across the 3-5 PKA inhibition related conditions (LFC_est_). We first identified all ohnolog pairs in which one ohnolog was activated (LFC_est_>1.5), and the other was not (LFC_est_ < 0.9). We retained ohnolog pairs in which the difference in LFC_est_ between the activated and non-activated ohnolog was greater than 0.9. The overlap between ${DE}_{PKA}$ and ${DE}_{Stress}^{Scer}$ (66 shared ortholog pairs) is significantly more than would be expected by chance (p=2.28E-32, Fisher’s exact test) (B) Plot of LFC_est_ for all ohnolog pairs after sorting ohnologs such that LFC_est_ for Ohnolog 1 is less than LFC_est_ for Ohnolog 1. On the left DE_PKA_ is highlighted and on the right ${DE}_{Stress}^{Scer}$ is highlighted. Dashed lines indicate the thresholds that define ${DE}_{Stress}^{Scer}.$ (C) The dependence of the size of ${DE}_{Stress}^{Scer}$(left) and its overlap with DE_PKA_ (right) on the parameter that define ${DE}_{Stress}^{Scer}$ are shown. The minimum difference in LFC_est_ between ohnolog pairs is fixed at 0.9. The dashed line indicates the value for the maximum LFC_est_ of Ohnolog 1 we used (0.9) and the black dot identifies the contour for the minimum LFC_est_ for Ohnolog 2 that we used. We chose these thresholds to yield a ${DE}_{Stress}^{Scer}$ whose size was on par with DE_PKA_, and to obtain as large an overlap as possible.

**Figure S13: The conclusion that PKA induction is the derived phenotype is independent of the species in which** $\boldsymbol{DE}_{\boldsymbol{Stress}}\mathbf{o}$**hnolog pairs are defined.** Distribution of LFC values for PKA related stress conditions (as in Fig 3B) is shown, except focusing on (A) ${DE}_{Stress}^{Scer}$, (B) ${DE}_{Stress}^{Vpol}$, and (C) ${DE}_{Stress}^{Ncas}$. Boxplots show median and Q1-Q3 range for normalized LFC (see Materials and Methods) for gene expression data from (Roy et al. 2013; Thompson et al. 2013) for the indicated species for the stress conditions most correlated to PKA inhibition in *S. cerevisiae* and *K. lactis* from (Fig S5). Boxplots for data from ${DE}_{Stress}$ genes from (A) *S. cerevisiae*, (B) *V. polymorpha* and (C) *N. castelliii* are shown towards the left side of each panel and boxplots for data from the orthologs in indicated species of those ${DE}_{Stress}$ genes are shown to the right. Blue and red indicate low-LFC and high-LFC ohnologs (respectively) and their syntenic orthologs in post-WGH species. Purple and grey bars are for the shared orthologs in Non-WGH *Saccharomycetaceae* species and outgroups respectively. Numbers in parentheses indicate the number of retained orthologs. Syntenic ortholog assignment for post-WGH species is based on the YGOB database (Byrne and Wolfe 2005).

**Figure S14:** **The conclusion that high basal expression is the ancestral phenotype is independent of the species in which** $\boldsymbol{DE}_{\boldsymbol{Stress}}$ **ohnolog pairs are defined.** Distribution of basal expression values as in Fig 3C is shown, except focusing on (A) ${DE}_{Stress}^{Scer}$ and (B) ${DE}_{Stress}^{Ncas}$. Boxplots showing median and Q1-Q3 range of normalized raw expression data (see Materials and Methods) are shown from microarray experiments comparing mRNA under exponential growth conditions to genomic DNA from (Tsankov et al. 2010). Boxplots for data from ${DE}_{Stress}$ genes from (A) *S. cerevisiae* and (B) *N. castelliii* are shown towards the left side of each panel and boxplots for data from the orthologs in indicated species of those ${DE}_{Stress}$ genes are shown to the right. Blue and red indicate low-LFC and high-LFC ohnologs (respectively) and their syntenic orthologs in post-WGH species. Purple and grey bars are for the shared orthologs in non-WGH *Saccharomycetaceae* species and outgroups respectively. Syntenic ortholog assignment for post-WGH species is based on the YGOB database (Byrne and Wolfe 2005).

**Figure S15: Non-overlapping sets of genes are differentially expressed in response to stress in different post-WGH species.**

(A) Overlap of all ohnologs that have data in (Roy et al. 2013; Thompson et al. 2013) between species as determined from YGOB pillars (Byrne and Wolfe 2005). The number on the diagonal is the total number of ohnologs with data in that species. (B) Overlap between ${DE}_{Stress}$ sets for various pairs of species. Shown below each Venn diagram is the p-value for a Fisher’s exact test on the null hypothesis that this overlap is expected based on the overlap of all ohnologs with data (shown in (A)) for any two given species. The number on the diagonal is the total number of ohnolog pairs in ${DE}_{Stress}$for that species. There is little overlap between orthologs of the ${DE}_{Stress}$ sets defined for different species. This is partially a result of the fact that the set of ohnolog pairs that is retained decreases as the evolutionary distance between species increases for post-WGH species (Scannell et al. 2007). However, the overlap of differentially expressed ohnolog pairs is even smaller than would be expected given the total percentage of retained ohnologs between species. Thus, there is no reason to expect a priori that the same conservation patterns for LFC and basal expression would hold for differentially expressed ohnolog pairs defined by their expression in distantly related post-WGH species (e.g.${DE}_{Stress}^{Scer}$ v.s. ${DE}_{Stress}^{Vpol}$).

Species abbreviations: Scer = *Saccharomyces cerevisiae*, Spar = *Saccharomyces paradoxus*, Sbay = *Saccharomyces bayanus,* Smik = *Saccharomyces mikatae*, Suva = *Saccharomyces uvarum*, Cgla = *Candida glabrata*, Ncas = *Naumovozyma castellii,* Vpol = *Vanderwaltozyma polyspora.*

**Figure S16: The STRE is localized closer to the start codon in the promoters of targets of PKA inhibition in *S. cerevisiae*.** Distribution of STRE distance from start codon for indicated gene sets for (A) *S. cerevisiae* and (B) *K. lactis*. N is the total number of STREs found in the indicated set, vertical lines indicate 100 and 400 base pairs from the start codon, and percentages indicate the fraction of STREs between 100 and 400 base pairs from the start codon.

**Figure S17: The TATA box is enriched in the promoters of targets of PKA inhibition, as well as in orthologs of DE_PKA_ genes in *S. cerevisiae* and *K. lactis*.** (A) Distribution of number of TATA boxes in the promoters of indicated sets in *S. cerevisiae* and (B)in *K. lactis*. (C) Distribution of the distance of TATA boxes from the start codon in the promoters of indicated sets for *S. cerevisiae* and (D) *K. lactis*. N is the total number of STREs found in the indicated set, vertical lines indicate 0 and 250 base pairs from the start codon, and percentages indicate the fraction of STREs between 0 and 250 base pairs from the start codon.

**Figure S18: There is a high percentage of promoters with both TATA boxes and STREs in genes induced by PKA in *S. cerevisiae*, which is expected based on the enrichment for both motifs in that set.** Percentages of promoters in the indicated sets with one or more STRE in combination with one or more TATA boxes in the 300bases upstream of the start codon for (A) *S. cerevisiae* and (B) *K. lactis*.

**Figure S19: STRE counts in the promoters of orthologs of select DE_PKA_ genes.** The number of STREs in the promoters of the orthologs of the subset of DE_PKA_ genes considered for Fig 5 are shown. Grey indicates either no ortholog exists or no promoter was found in the dataset. Columns are different species and rows are clustered based on the number of STREs in non-WGH species (ZT branch, KLE branch, and outgroups). The dendrogram for the hierarchical clustering is shown to the left. Clusters highlighted in Fig 5C are indicated in blue and green boxes.

**Figure S20:** **GPM2/3 are an example of a differentially induced pair of ohnologs in which the STRE arose in the promoter of the DE_PKA_ high-LFC ohnolog following the WGH.** (A) Phylogenetic tree of all orthologs of GPM2/3 from the *Saccharomycetaceae* clade from (Shen et al. 2018) plotted alongside each gene’s promoter (700 bp upstream of the start codon) with STRE (red triangle) and TATA box (blue triangles) motifs highlighted. The arrow indicates the putative point at which the STRE was gained. The first column of boxes after each promoter represents the number of STREs and the second columns of boxes indicates whether there is a TATA box within 300 bases of the start codon. Phylogeny is determined from a multiple sequence alignment of the protein sequences (see Materials and Methods). Support values (bootstrap/alrt) are shown to the left and below each branch point, and branch lengths (amino acid substitutions/site) are shown above. Shading represents different groups of species; blue = Post-WGH, syntenic ortholog to low-LFC ohnolog; red = Post-WGH, syntenic orthologs to high-LFC ohnolog; yellow=Post-WGH, synteny not determined; green= ZT; light purple=KLE; dark purple=other Non-WGH; grey=outgroups. (B) Estimated regularized log expression (rlog) with and without 3µM 1-NM-PP1 in GPM2, GPM3, and their shared ortholog in *K. lactis*.

**Figure S21: The presence of STREs in the promoters of GPM2/3 and EGO2/4 orthologs tends to correspond to increased responsiveness to PKA-related stress conditions.** Phylogenetic trees of orthologs of (A) GPM2/3 and (B) EGO2/4 which were included in the gene expression datasets from (Thompson et al. 2013) and (Roy et al. 2013). In addition to plotting a diagram of the gene’s promoter with the location of STRE and TATA box motifs indicated as in Figures S20 and S22, gene expression data from the 5 indicated PKA-related stress conditions is plotted. Dark grey boxes indicate that no data for that gene were present for that condition. *N. castellii* contained two syntenic orthologs for EGO4, the high-LFC ohnolog of EGO2/4, indicating that there was a small-scale duplication of the gene in that lineage. Two conditions in *N. castellii* contained values greater than the maximum value for our color bar, so the values are included in the boxes for those genes. Bootstrap support values are shown to the left of each branch point. Shading represents different groups of species; blue = Post-WGH, syntenic ortholog to low-LFC ohnolog ; red = Post-WGH, syntenic orthologs to high-LFC ohnolog ; yellow=Post-WGH, synteny not determined; light purple=KLE.

**Figure S22: EGO2/4 are examples of a differentially induced ohnolog pair in which the STRE arose in the ZT branch prior to the WGH.** (A) Phylogenetic tree of all orthologs of EGO2/4 from the *Saccharomycetacea*e clade from (Shen et al. 2018) plotted alongside each gene’s promoter. Conventions and nomenclature are as in Fig S18. (B) Estimated regularized log expression (rlog) with and without 3µM 1-NM-PP1 in EGO2, EGO4, and their shared ortholog in *K. lactis*.

**Figure S23: The expression of many DE_PKA_ high LFC ohnologs depends on Msn2/4.** RNA seq data for PKA-AS strains with and without (A) Msn2/4 in *S. cerevisiae* or (B) their shared ortholog in *K. Lactis* in the presence of 3µM 1-NM-PP1 at 50 minutes. The solid black line is 1:1. The dashed blue line is at the LFC threshold of 2.0 for PKA activated genes. The dashed line in (A) is a regression line based on the genes with a negative LFC in WT cells to illustrate a general decrease in the response to PKA for both repressed and activated genes in ΔMsn2/4 cells. The line has a slope of 0.57, an intercept of 0.15, and an R value of 0.81 with the genes with negative LFC in WT cells.
